# The Transcription Axes ERK-Elk1, JNK-cJun, and JAK-STAT Promote Autophagy Activation and Proteasome Inhibitor Resistance in Prostate Cancer Cells

**DOI:** 10.3390/cimb47050352

**Published:** 2025-05-12

**Authors:** Georgios Kalampounias, Kalliopi Zafeiropoulou, Theodosia Androutsopoulou, Spyridon Alexis, Argiris Symeonidis, Panagiotis Katsoris

**Affiliations:** 1 Laboratory of Cell Biology, Division of Genetics, Cell and Developmental Biology, Department of Biology, School of Natural Sciences, University of Patras, 26504 Patras, Greece; gkalampounias@ac.upatras.gr (G.K.); kzafeirop@upatras.gr (K.Z.); tandroutsopoulou@ac.upatras.gr (T.A.); 2 Hematology Division, Faculty of Medicine, School of Health Sciences, University of Patras, 26504 Patras, Greece; spirosal1@hotmail.com (S.A.); argiris.symeonidis@yahoo.gr (A.S.)

**Keywords:** prostate cancer, proteasome-ubiquitin system, bortezomib resistance, oxidative stress, autophagy, cJun, STAT, Elk1

## Abstract

The rapid emergence of resistance limits the application of proteasome inhibitors against solid tumors, despite their effectiveness in the treatment of hematological malignancies. Resistant phenotypes are complex and multifaceted, and, thus, the mechanisms involved have not been adequately described. In this study, a Bortezomib-resistant prostate cancer cell line is created by using the PC-3 cell as a prostate carcinoma model of high metastatic potential. The main biochemical differences and adaptations exhibited by the resistant cells revolve around apoptosis evasion, autophagy induction (functioning as a ubiquitin-proteasome system substitute), expression of epithelial-to-mesenchymal transition markers, and increased aggressiveness. Broad-spectrum signaling pathway analyses also reveal an upregulation and activation of Nf-κB, STAT3, cJun, and Elk1 transcription factors in the resistant cells. Additionally, intracellular reactive oxygen species assays reveal a downregulation in resistant cells, which is theorized to be a consequence of metabolic changes, increased autophagic flux, and antioxidative enzyme action. These findings expand our understanding of proteasome inhibitor resistance and highlight key kinases and transcription factors as novel potential therapeutic targets. Effective inhibition of resistance-specific pathways could re-sensitize the cells to proteasome inhibitors, thus surpassing current therapeutic limitations.

## 1. Introduction

Prostate cancer (PCa) is the most frequent type of cancer diagnosed among men in the EU, accounting for about 25% of all annual cancer diagnoses. Even though most PCa forms start as androgen-dependent hyperplasia, due to mutations and selective pressure, the dependence on androgen receptor (AR) stimulation is gradually lost and evolves into androgen-independent prostate cancer, which is far more aggressive and is correlated with poor outcomes [1]. Prostate adenocarcinoma can evolve into metastatic (or advanced) prostate cancer, which is a severe form of malignancy that may affect the lymph nodes, lungs, or other organs and has a very poor prognosis [2]. Hormone therapy in the form of testosterone suppression is employed to shrink tumors and extend patient survival; however, once the cancer cells have been desensitized to AR stimulation and the cancer has evolved into metastatic castrate-resistant prostate cancer (mCRPC), hormone therapy is ineffective [2]. mCRPC affects about 10–20% of PCa patients within five years of follow-up after their initial therapy, and it is the most advanced and fatal form of PCa [3]. Current pharmaceutical approaches have limited efficacy against mCRPC and are often accompanied by a set of unbearable collateral effects that significantly impact treated patients. Many substances are screened as potential treatments, among which lies Bortezomib, a proteasome inhibitor (PI) that has saved thousands of lives from multiple myeloma, mantle cell lymphoma, and many other hematological malignancies [4]. The drug (either alone or in combination with other agents) has been tested against solid tumors (prostate cancer being one of them); however, the results were not satisfying enough to support PIs as a treatment [5]. Many articles attempted to provide a mechanistic explanation; nonetheless, up to this day, no definite answer has been given [6,7,8,9,10,11]. Nonetheless, molecular characterization of tumors and in vitro assays of cancer cells have revealed significant insight into PI mechanistic actions. Recently, Bortezomib has drawn renowned attention as a potential therapy for mCRPC patients with *PTEN* deleterious mutations [12].

PIs target the catalytic sites of the 26S proteasome, a protein multi-catalytic complex that actively participates in the cell’s homeostatic mechanisms by selectively degrading polyubiquitinated polypeptides In cancer cells, the proteasome is of paramount importance, and its increased activity and subunit accumulation are well-documented in various cancer types. It is believed that cancer cells, due to increased biosynthetic rates and miss-regulated control mechanisms, produce more miss-folded polypeptides that need to be recycled [13,14]. Additionally, proteasomal degradation regulates the turnover rates of proteins mediating cell cycle progression/arrest, mitochondrial function, and gene expression [15,16]. Cancer cells have been found to rely on the proteasome to “tune” this system, and this mechanism, namely the ubiquitin-proteasome system (UPS), is a very important pathway that, once targeted and sabotaged, can induce apoptosis and lead to cell death [17]. The activity of the proteasome inside the cell is of high importance for cell survival and, thus, is regulated at several levels, as summarized by Livneh et al. in 2016 [18]. Given its important role in cell homeostasis, many regulating molecules have been investigated as pharmaceutical targets over the years for cancer as a means to deregulate them, make the malignant cells more susceptible to stress-caused damage and apoptotic signals, and eventually lead to their apoptotic death. Among them, PIs constitute a drug class of proteasome subunits inhibitors, and have entered clinical practice as a therapy against the aforementioned hematological malignancies. Since their discovery, PIs have been tested on various cell lines, animal models, and even clinical studies; however, besides a group of hematological cancers, they are not very efficient against solid tumors as resistance develops [6,8,9,19,20,21,22,23,24].

The first PI with documented tumoricidal actions was Bortezomib, which acts by binding to β5 subunits, the main catalytic subunits of the 20S core particle that exhibit chymotrypsin-like (ChT-L) activity [25]. Patients who were treated with Bortezomib and relapsed due to resistance were found to have mutations in the *PSMB5* gene, the one coding for the β5 subunit, that significantly reduced the drug binding ability or caused increased expression [26,27,28,29,30,31]. However, mutations are not unique in resistance emergence and driving, since most of the relapsed patients did not have alterations regarding β5 structure or expression. The current hypothesis is that, besides genetic mutations affecting the subunits’ abundance and structure (thus rendering Bortezomib insufficient), changes in the signaling cascades that regulate apoptosis, autophagy, and oxidative stress also play a huge part in resistant cells [11,32,33,34,35,36]. Nf-κB was one of the first molecules to be found active in Bortezomib-resistant cells, implying a role in the emergence of that aggressive phenotype [37]. The STAT family is another class of transcription factors that have been found active in resistant clones, as well as the ERK1/2 signaling pathway that mostly regulates cell survival and proliferation [38,39,40,41,42,43]. Recent advances in Bortezomib resistance, focused on prostate cancer, report the activation of autophagy in the resistant cells and describe a regulation mechanism that substitutes the impaired UPS [11,44,45,46,47]. Additionally, key signaling molecules and oncogenes like Elk1, cJun, cSrc, and epithelial to mesenchymal (EMT) markers have not been adequately investigated up to this day. Therefore, given the complexity of resistance development and the multiple aspects of resistant phenotypes, the need for a new approach is of paramount importance.

The first PI with documented tumoricidal actions was Bortezomib, which acts by binding to β5 subunits, the main catalytic subunits of the 20S core particle that exhibit chymotrypsin-like (ChT-L) activity [25]. Patients who were treated with Bortezomib and relapsed due to resistance were found to have mutations in the *PSMB5* gene, the one coding for the β5 subunit, that significantly reduced the drug binding ability or caused increased expression [26,27,28,29,30,31]. However, mutations are not unique in resistance emergence and driving, since most of the relapsed patients did not have alterations regarding β5 structure or expression. The current hypothesis is that, besides genetic mutations affecting the subunits’ abundance and structure (thus rendering Bortezomib insufficient), changes in the signaling cascades that regulate apoptosis, autophagy, and oxidative stress also play a huge part in resistant cells [11,32,33,34,35,36]. Nf-κB was one of the first molecules to be found active in Bortezomib-resistant cells, implying a role in the emergence of that aggressive phenotype [37]. The STAT family is another class of transcription factors that have been found active in resistant clones, as well as the ERK1/2 signaling pathway that mostly regulates cell survival and proliferation [38,39,40,41,42,43]. Recent advances in Bortezomib resistance, focused on prostate cancer, report the activation of autophagy in the resistant cells and describe a regulation mechanism that substitutes the impaired UPS [11,44,45,46,47]. Additionally, key signaling molecules and oncogenes like Elk1, cJun, cSrc, and epithelial to mesenchymal (EMT) markers have not been adequately investigated up to this day. Therefore, given the complexity of resistance development and the multiple aspects of resistant phenotypes, the need for a new approach is of paramount importance.

The purpose of this study is to focus on a model of androgen-independent aggressive PCa and thoroughly study cell functions, signaling cascades, and transcription factors to come up with novel data regarding resistance development against PIs. For this purpose, PC-3 cells are used as a model of resistance-emergence, selected for their very specific set of characteristics [48]. PC-3 cells are derived from bone metastasis of a prostate adenocarcinoma patient and are considered a well-established model for PCa, known for their high metastatic potential (higher than those of other models, like DU-145 cells) [49]. Additionally, their abolition of androgen-dependence corresponds to aggressive types of cancer observed in advanced-stage patients. Previous data from our laboratory encouraged research on a new model that would have a more aggressive, poorly differentiated phenotype, as it would better simulate in vivo conditions. Moreover, PC-3 cells are homozygous for a deleterious *PTEN* mutation and express characteristics aligned with progenitor cells, exhibiting stemness markers [50,51]. PC-3 cells can allow for a safer extrapolation of conclusions concerning mechanisms exploited by various cancer types. Given the recent advances in drug resistance research and the better understanding of the signaling pathways we now have, we believe that focusing on the new molecular players in this chess game, unraveling and finally targeting their regulation mechanisms, could renew interest in proteasome inhibitor therapy, thus better arming us against aggressive cancer types and improving patient life quality.

## 2. Materials and Methods

### 2.1. Cell Culture

The PC3 (ATCC, Manassas, VT, USA) cell line was used as a human prostate carcinoma cell model. The cells were grown in RPMI 1640 medium supplemented with 10% fetal bovine serum (FBS), 100 units/mL penicillin, and 100 μg/mL streptomycin and maintained at 5% CO_2_ and 100% humidity at 37 °C. Cell culture media (RPMI 1640 with stable glutamine) and cell culture-related reagents (FBS, 0.25% trypsin solution in PBS, and penicillin/streptomycin) were purchased from Biowest (Nuaille, France). Cell culture dishes, microplates, and Transwell chambers were from Greiner Bio-One (Kremsmünster, Austria). Flow cytometry expendables and reagents were from BD Biosciences (Franklin Lakes, NJ, USA). The proteasome inhibitor Bortezomib was purchased from Janssen-Cilag International (Velcade^®^, Beerse, Belgium), Carfilzomib was from Amgen Inc. (Kyprolis^®^, Breda, The Netherlands), Doxorubicin was purchased from Pfizer Hellas (Adriblastina^®^, Athens, Greece) and Paclitaxel was from Ratiopharm (Pazenir, Ulm, Germany). A detailed list of reagents and consumables can be found in Appendix A (Table A1).

### 2.2. Proliferation Assays

Equal cell numbers were seeded inside 48-well culture plates and left to attach and grow for 24 h. After this interval, the medium was aspirated, and fresh medium with increasing concentrations of Bortezomib (0, 5, 10, 20, 50, 100, 150, and 200 nM concentration selection based on preliminary experiments and previously published papers) was added, and the cells were incubated for 72 h with the drug. Each Bortezomib concentration was administered in triplicate wells. Subsequently, the media were aspirated, and the adherent cells (alive) were fixed with 4% *v/v* formaldehyde in PBS for 15 min and then stained with 0.5% crystal violet in 25% methanol for 20 min. Following gentle rinses with water, the plates were left to air-dry, and the retained crystal violet was extracted using a 30% acetic acid aqueous solution. Afterward, the optical density at 595 nm was measured. The same procedure was followed to calculate the Carfilzomib IC_50_ (by using the same concentration range as with Bortezomib), the Doxorubicin IC_50_ (by incubating the cells with concentrations ranging from 0.3 to 3 μM; a range based on known IC_50_ values and preliminary experiments), and the Paclitaxel IC_50_ (by using the same concentration range as with Bortezomib; also based on known IC_50_ values and preliminary experiments).

### 2.3. Creation of the PC-3 RB40 Subline

To create a cell line resistant to the proteasome inhibitor Bortezomib, the procedure described by Zafeiropoulou et al. was followed with slight modifications [11]. The IC_50_ of non-resistant cells was calculated (designated as naïve PC-3 cells) following 72 h of Bortezomib incubation, and half of this concentration was added to cell culture dishes (with 75% confluency). The cells were left to grow under constant drug presence, and the medium was replaced every 72 h, constantly maintaining the same Bortezomib concentration (5 nM) for three passages (~14 days). Following adaptation to this concentration, the drug dose was changed to 10 nM and maintained for two passages (~14 days). The same procedure was repeated for the 15, 20, 25, and 30 nM milestones. Raising the inhibitors’ dose was not well-tolerated by the cells, and many days were required for the cells to divide. The cells needed circa three months to reach the 30 nM milestone; they were maintained for four weeks at this concentration, and after this interval, they were supplemented with 35 nM of the drug for six weeks. Finally, the dose was stabilized at 40 nM of Bortezomib, while cell growth remained impaired for another two months. After about five months of ever-increasing Bortezomib doses (from 0 to 40 nM) and two months of stable 40 nM Bortezomib presence, the cells were adapted to the drug dosage. At this point, assays concerning cell viability, migration, apoptosis, autophagy, intracellular signaling, and oxidative stress were performed. The resulting cell clone, resistant to the proteasome inhibitor, was named PC-3 RB40 (Resistant-Bortezomib 40 nM). Naïve PC-3 cells (of the same passage) were used as a control group. All cells were preserved in liquid hydrogen vapors and were thawed (1–2 passages before the assays) to prevent extended passaging. To screen for any differences between resistant and non-resistant cells, as well as detect deviations from the parental clone due to multiple cell generations, cells (resistant and non-resistant) of similar passages (passage difference ≤ 3) were used throughout all assays.

### 2.4. Flow Cytometry

The FACS Calibur (BD Biosciences, Franklin Lakes, NJ, USA) was used to assess cell viability, cell cycle progression, apoptosis, lysosomal activity, and intracellular reactive oxygen species levels. The cells were incubated for specific time intervals in a Bortezomib-containing medium, and, after that, they were collected by trypsinization and centrifugation. The cell number was estimated using a Neubauer hemocytometer (Corning, Corning, NY, USA), and equal numbers of cells were used for the analyses. To assess apoptosis, cells were incubated in Bortezomib-containing medium for 12, 24, and 48 h (based on preliminary assays) and then harvested as previously described. Afterward, they were stained with propidium iodide (PI) and Annexin V-FITC (BD Biosciences, Franklin Lakes, NJ, USA) for 15 min at room temperature in the dark [52]. To measure the lysosomal activity, cells were stained with LysoTracker™ Red DND-99 (Invitrogen™ (Thermo Fisher Scientific), Waltham, MA, USA) diluted in serum-free RPMI 1640 medium at 37 °C for 30 min in the dark. To measure ROS, cells were stained with H_2_DCFDA (Invitrogen™ (Thermo Fisher Scientific), Waltham, MA, USA) at 37 °C for 30 min following an already described procedure [53,54]. To analyze only the viable cells, the cell viability kit LIVE/DEAD (Invitrogen™ (Thermo Fisher Scientific), Waltham, MA, USA) was used in all stainings, and the cells were appropriately gated. Each flow cytometry experiment was conducted in triplicate. The subsequent analysis was performed with the FlowJo V10 software (BD Biosciences, Franklin Lakes, NJ, USA).

### 2.5. Western Blots

Cells were cultured without drugs/inhibitors for 24 h (a time point based on previous publications and preliminary experiments), and then incubated with the designated concentrations of Bortezomib or Doxorubicin for varying times. Subsequently, they were washed twice with an ice-cold PBS solution and lyzed using RIPA buffer (Thermo Scientific™ (Thermo Fisher Scientific), Waltham, MA, USA). The extracts were aliquoted and kept at −24 °C until the analysis. Total proteins were determined using the Bradford assay. Equal amounts of total proteins were mixed with Laemmli’s Sample Buffer 2× solution containing 5% β-ME, and the samples were denatured at 95 °C for 10 min. Proteins were separated using 12% polyacrylamide gels and transferred to an Immobilon-P membrane (Merck Millipore (Merck), Burlington, MA, USA) for 30 min using Towbin’s transfer buffer in a semi-dry transfer system as described in Zafeiropoulou et al. [11]. The membrane was blocked in TBS containing 5% skimmed milk and 0.1% Tween-20 for 1 h at 37 °C. Membranes were then incubated with primary antibodies (Table A2) diluted in blocking solution overnight at 4 °C, under continuous agitation. All antibodies were either from Cell Signaling Technology (Danvers, MA, USA) (annotated as ‘CST’ in Table A2) or Santa Cruz Biotechnology (Dallas, TX, USA) (annotated as ‘sc’ in Table A2).

The blot was then incubated with the appropriate secondary antibodies (Anti-rabbit IgG Antibody, CST#7074, or Anti-mouse IgG antibody, CST# 7076) (both diluted 1:2000) coupled to horseradish peroxidase, and bands were detected using the SuperSignal™ West Femto Maximum Sensitivity Substrate Thermo Scientific™ (Thermo Fisher Scientific, Waltham, MA, USA), according to the manufacturer’s instructions. Where indicated, blots were stripped in buffer containing 62.5 mM Tris HCl pH 6.8, 2% SDS, and 100 mM 2-mercaptoethanol for 30 min at 50 °C and reprobed with primary antibodies. The blots were developed on Super RX-N medical X-Ray film (Fujifilm Hellas, Patras, Greece), and the films were scanned to analyze the band size and intensity. Quantitative estimation of the detected protein was performed through analysis of digital images using the ImageJ built-in ‘Gels’ tool. Each experiment was conducted three times and average intensities were extracted. Besides the Bradford assay, which was used to calculate the loading volumes, the gels and membranes were stained with Coomassie Brilliant Blue (Sigma-Aldrich (Merck), Darmstadt, Germany) after the analysis to validate the equal protein quantities. β-actin was used as a reference protein since its accumulation did not exhibit fluctuation between naïve and resistant cells.

### 2.6. Scratch Assay

The scratch test/wound healing assay was used to assess the wound healing rate of both naïve and resistant cells. Cells were seeded inside 6-well cell culture plates and left to form a monolayer. Using a sterilized P200 pipette tip (Greiner Bio-One, Kremsmünster, Austria), cruciform scratches were done, and the culture medium was aspirated. The cells were rinsed gently with a warm PBS solution (37 °C), and, subsequently, a fresh medium containing increasing concentrations of Bortezomib (0, 20, 40, and 80 nM) was added. Each Bortezomib dose was monitored in triplicate. Photographs were taken using a microscope-mounted camera (Nikon, Tokyo, Japan). The microplate was photographed at key intervals of 0, 24, 48, and 72 h. The wound healing rate was calculated using the Wound Healing plugin for ImageJ [55].

### 2.7. Transwell/Boyden Chambers

Migration and chemotaxis were assessed in Transwell/Boyden chambers with 8 μm filter pores [56]. A serum-containing medium was added to the lower compartment (with or without Bortezomib), and 2 × 10^4^ cells (suspended in serum-free RPMI 1640) were added to the insert. The cells were left to migrate for 24 h, and then the filters were fixed using a 4% *v/v* formaldehyde in PBS solution. Cells from the upper side of the filters were scraped, and the remaining (migrated) cells were stained using a 0.5% crystal violet solution. The filters were photographed under a microscope, and the total cells on the filters were counted using the Cell Counter built-in tool for ImageJ (v.1.54m).

### 2.8. Statistical Analysis

All statistical analyses were performed using IBM^®^ SPSS^®^ (version 29.0.0) (IBM^®^, Armonk, NY, USA). Plots were created using Prism 8 (GraphPad, La Jolla, CF, USA). Normality of the data was checked using Shapiro–Wilks test. The proliferation assays were analyzed using Prism built-in non-linear regression equations to calculate the IC_50_ values. Comparisons between the IC_50_ were performed using Fisher’s exact test (F-test). Cell cycle analysis was performed using FlowJo to plot histograms and extract the number of cells belonging to each phase/subset. Subsequent analyses were performed by comparing each sample with the control (untreated naïve PC-3 cells) using chi-square tests. Apoptosis analysis was performed by statistically comparing the cells of each quartile in the Annexin V-FITC/PI density plots. Statistical analysis was performed using chi-square tests. Autophagy and oxidative stress analysis was performed using one-way Analysis of Variance (ANOVA) and Tukey’s multiple comparisons tests. Statistical analysis of time-course ROS data was analyzed using two-way ANOVA. The wound healing rate was estimated using the average rate of three independent experiments as calculated using FIJI (version 2.9.0) [57]. Statistical analysis was performed using two-way ANOVA. Migration assays were analyzed using one-way ANOVA and multiple comparisons. Following scanning, Western blot (film) data were collected using FIJI software, and were subsequently analyzed using one-way ANOVA as described in previous publications [58]. Protein quantification data were plotted in heatmaps and bar charts using Prism 8.

## 3. Results

### 3.1. Creation of the PC-3 RB40 Resistant Clone and Assessement of Their Viability, Cell Cycle Progression, and Apoptosis

#### 3.1.1. Cytotoxicity Assays and IC_50_ Determination

**Figure A1:**
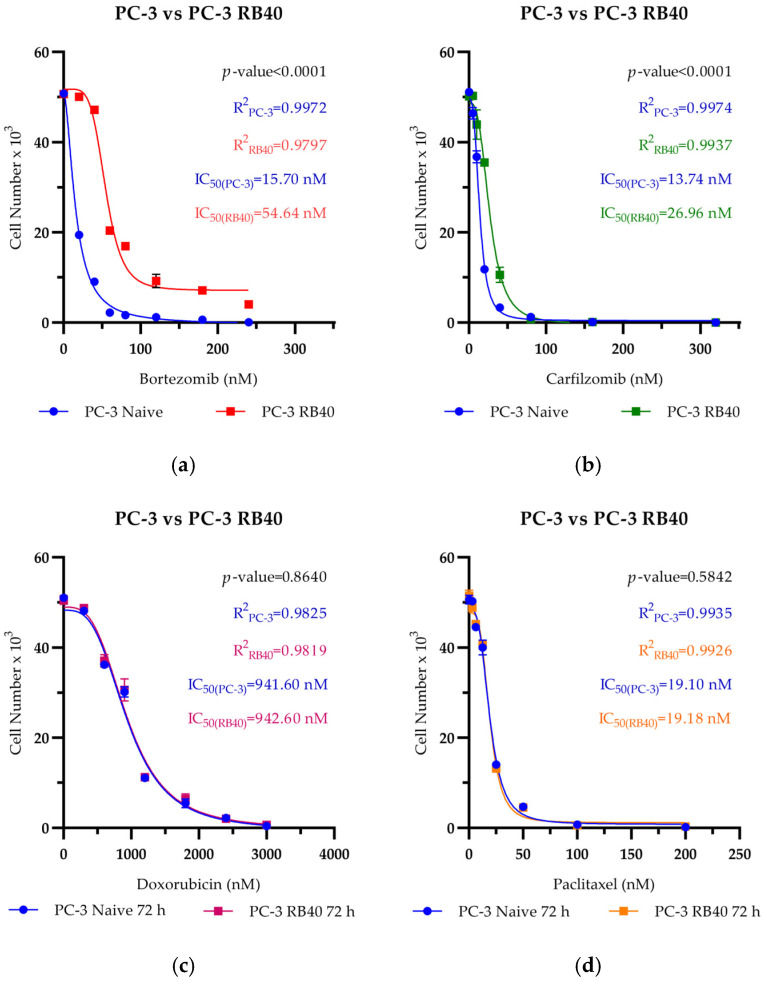
Representative viability assays of (**a**) Bortezomib; (**b**) Carfilzomib; (**c**) Doxorubicin; and (**d**) Paclitaxel. Both naïve PC-3 and PC-3 RB40 cells were incubated with the drugs for 72 h. Each dot represents the average of three experimental values, and the error bars represent the standard error of the mean (SEM). The fitting lines were graphed in Prism 8 using the built-in model for IC_50_ determination. Blue lines represent naïve PC-3 cells, the red line corresponds to PC-3 RB40 cells treated with Bortezomib, the green line represents PC-3 RB40 cells treated with Carfilzomib, the magenta line stands for PC-3 RB40 cells treated with Doxorubicin, and the orange line corresponds to cells treated with Paclitaxel.

After long-term exposure to increasing concentrations of Bortezomib, the PC-3 cell line gradually developed resistance to the drug (Figure S1a–d). During the dose escalation, the cell morphology was altered, and the cells were used to create long protrusions instead of maintaining the typical PC-3 morphology. This phenomenon was evident in naïve cells and also persisted in resistant cells during the first three weeks of stable Bortezomib administration (40 nM). This was theorized as a sign of poor adaptation and stress, and the signaling assays were not conducted until cell morphology was restored. During this interval, proliferation assays were performed, and the IC_50_ of the resistant cells was just above the stable medium concentration of 40 nM (Table 1) (Figure S1a,d). Following a total of 32 weeks, the cell morphology was restored, and the resistant cell clone had elevated its resistance capacity from 40 nM to about 55 nM, as shown by their IC_50_ values (Figure S1e). Since the 40 nM dose was the lowest concentration where no naïve PC-3 cells survived after 72 h of treatment while the resistant cells were adequately adapted, it was established as the main context for signal transduction, apoptosis, and stress assays. This cell clone of PC-3 was named PC-3 RB40 and was constantly cultured in RPMI 1640 medium supplemented with 40 nM Bortezomib (Figure A1a). During all this interval, the cells were also being assessed for cross-resistance against the second-generation proteasome inhibitor Carfilzomib (Figure A1b and Figure S2a,b). Additionally, resistance against the chemotherapeutics Doxorubicin (Figure S3a,b) and Paclitaxel (Figure S4a,b) was assessed, both of which are tumoricidal agents known to induce apoptosis. Normally, PC-3 cells are susceptible to both Doxorubicin (which has a 72-h IC_50_ value of ~940 nM) and Paclitaxel (which has a 72-h IC_50_ value of 19 nM) (Table 1) (Figure A1c,d). Our experiments showed that the RB40 cell clone had the same IC_50_ as the naïve cells, indicating that no multidrug resistance mechanism had emerged (Figure A1c). The observed resistance was specific to proteasome inhibitors (Table 1). Besides Bortezomib, which is a first-generation PI, the RB40 clone also exhibited cross-resistance to Carfilzomib (a second-generation inhibitor); however, the IC_50_ value was significantly lower (Figure A1b). These results were confirmatory of a previous study conducted in our laboratory, in which, DU-145 cells were employed as a model for Bortezomib resistance. The resulting phenotype was a PI-specific resistant cell line (Bortezomib and Carfilzomib), while resistance against anthracyclines was not documented [11].

#### 3.1.2. Cell Cycle Analysis

**Figure 1:**
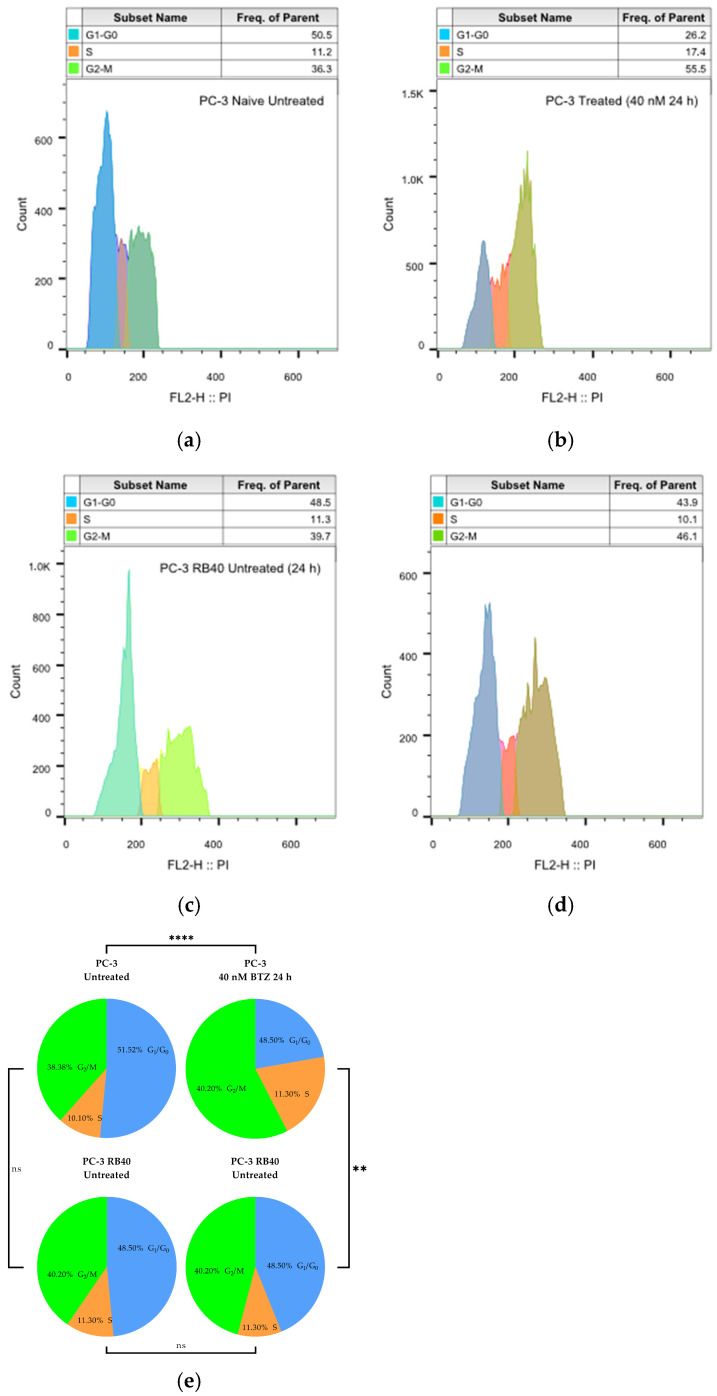
Cell cycle analysis of PC-3/PC-3 RB40 cells, following treatment with Bortezomib for 24 h. Representative samples of the following: (**a**) untreated PC-3 cells (experimental control of baseline cell cycle progression); (**b**) PC-3 cells treated with 40 nM of Bortezomib; (**c**) untreated PC-3 RB40 cells; and (**d**) PC-3 RB40 cells treated with 40 nM of Bortezomib (normal culture conditions for this cell line). (**a**–**d**) Blue histograms correspond to cells in the G_0_ and G_1_ phases. Orange histograms correspond to cells in the S phase. Green histograms correspond to cells in the G_2_ and M phases. Different shades of the colors are used to distinguish between the different samples. (**e**) Chi-square tests were performed between all samples. The term “ns” corresponds to non-significant; ** corresponds to a *p*-value = 0.001; and **** corresponds to a *p*-value < 0.0001).

To validate the resistant cells’ adaptation to the proteasome inhibitor-rich medium, we assessed their cell cycle progression and their apoptosis rate and compared them to those of naïve cells (Table A3). Bortezomib has been demonstrated to cause both G_1_/S arrest as well as G_2_/M arrest, and this mechanism also triggers apoptosis [59,60,61]. Naïve PC-3 cells, following 24 h of treatment with 40 nM of Bortezomib, significantly differed from untreated cells (*p*-value < 0.0001), and exhibited the bibliographically reported G_2_/S arrest (and, to a lesser extent, G_1_/S arrest), which is shown as an accumulation of cells in the second peak (corresponding to G_2_- and M-phase DNA content) as shown in Figure 1a,b. At the same dose, PC-3 RB40 cells indicated only a mild cell cycle distortion (Figure 1d) which did not differ significantly compared to untreated naïve (*p*-value = 0.5310), or untreated PC-3 RB40 cells (*p*-value = 0.7366). PC-3 RB40 cells were shown to maintain (untreated) naïve-like cell cycle progression even after incubation with 40 nM of Bortezomib. The untreated PC-3 RB40 cells were not documented to differ from naïve cells (*p*-value = 0.9095), regarding cell cycle phases’ distribution (Figure 1c). All comparisons were performed with chi-square tests, and they are visualized in Figure 1e.

#### 3.1.3. Apoptosis Assays

**Figure 2:**
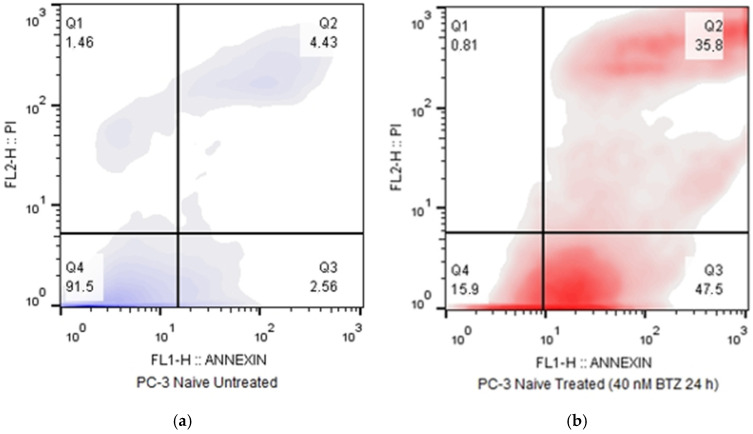

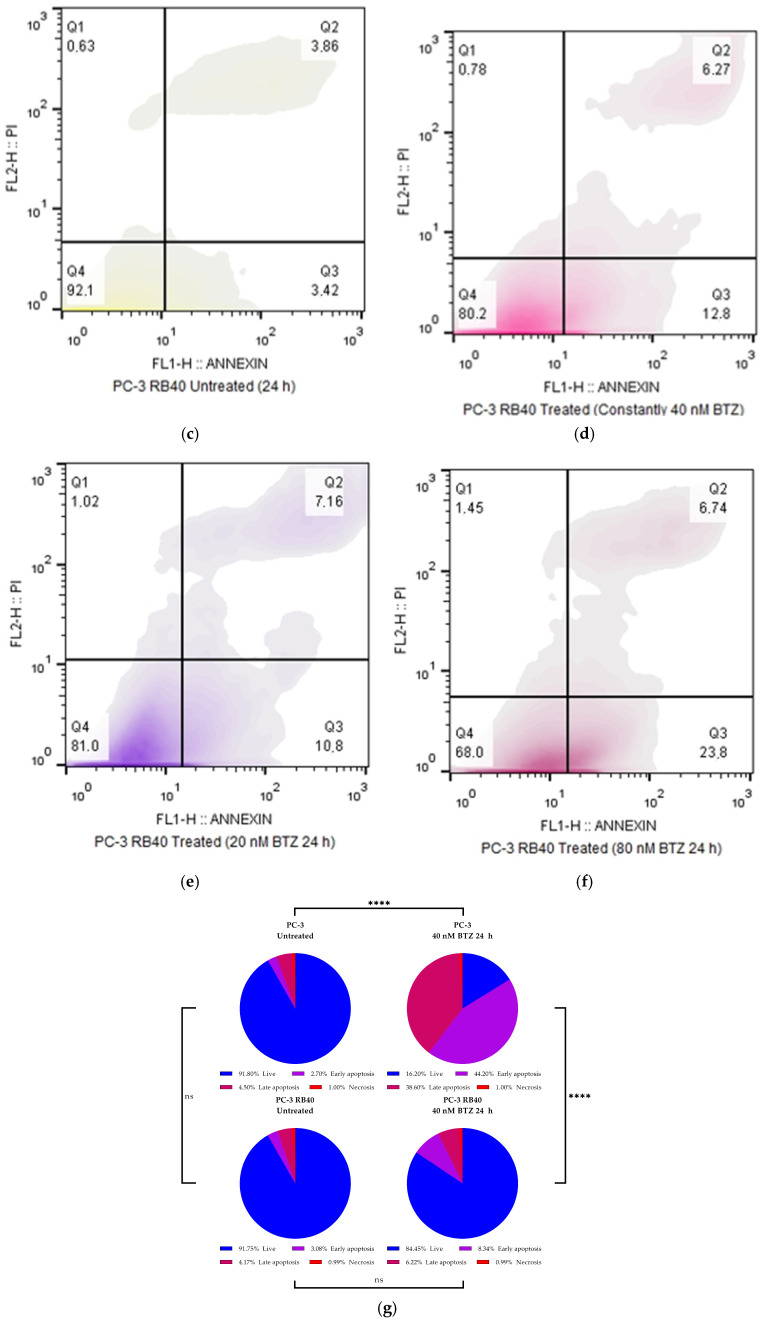
Apoptosis assay of PC-3/PC-3 RB40 cells, following treatment with Bortezomib for 24 h. Representative density plots of the following: (**a**) untreated PC-3 cells (blue coloring)(experimental control of baseline cell apoptosis level); (**b**) PC-3 cells treated with 40 nM of Bortezomib for 24 h (red coloring); (**c**) untreated PC-3 RB40 cells (yellow coloring); (**d**) PC-3 RB40 cells treated with 40 nM of Bortezomib (magenta coloring)(basal culture conditions for this cell line); (**e**) PC-3 RB40 cells treated20 nM of Bortezomib (purple coloring); and (**f**) PC-3 RB40 cells were cultured for 24 h in RPMI 1640 medium supplemented with 10% FBS and 80 nM of Bortezomib (lilac coloring). (**a**–**f**) The four different quartiles correspond to Q1: necrotic cells; Q2: cells in late apoptosis; Q3: cells in early apoptosis; and Q4: live cells. (**g**) Chi-square tests were performed between all samples. The term “ns” corresponds to non-significant and **** corresponds to a *p*-value < 0.0001).

The resistant cells were also assessed for their apoptosis rate and compared to naïve PC-3 cells (Table A4). An Annexin-V/PI kit was used that stains phosphatidylserine residues to indicate their extracellular presence (early apoptotic marker) and the membrane’s integrity, which is a marker of late apoptosis or necrotic/ferroptotic cell death. During resistance development, cells cultured with increasing doses of Bortezomib were initially susceptible to apoptosis induction, despite the constant drug presence. However, following eight weeks of incubation with a stable, high Bortezomib dose of 40 nM, they fully adapted. Apoptosis assessment made this evident, as the apoptotic activity of PC-3 RB40 cells (cultured with Bortezomib) had dropped to levels almost indistinguishable (*p*-value = 0.2422) to those of naïve cells (Figure 2a,c–e), while the treated cell population of naïve cells indicated high levels of apoptotic death (Figure 2b) (*p*-value < 0.0001). Treated PC-3 RB40 cells did not indicate any significant difference from untreated PC-3 RB40 cells (*p*-value = 0.3995). On the contrary, further dose escalation on the resistant clone (up to 80 nM) significantly induced apoptosis (*p*-value = 0.0189). Nevertheless, even this high Bortezomib dose (Figure 2f) did not cause cell death comparable to that of naïve cells (Figure 2b) (*p*-value < 0.0001). All comparisons were performed with chi-square tests, and they are visualized in Figure 2g.

### 3.2. Autophagy Substitutes the Impaired Proteasome-Ubiquitin System in Resistant Cells

#### 3.2.1. Proteasome-Ubiquitin System Assessment

**Figure 3:**
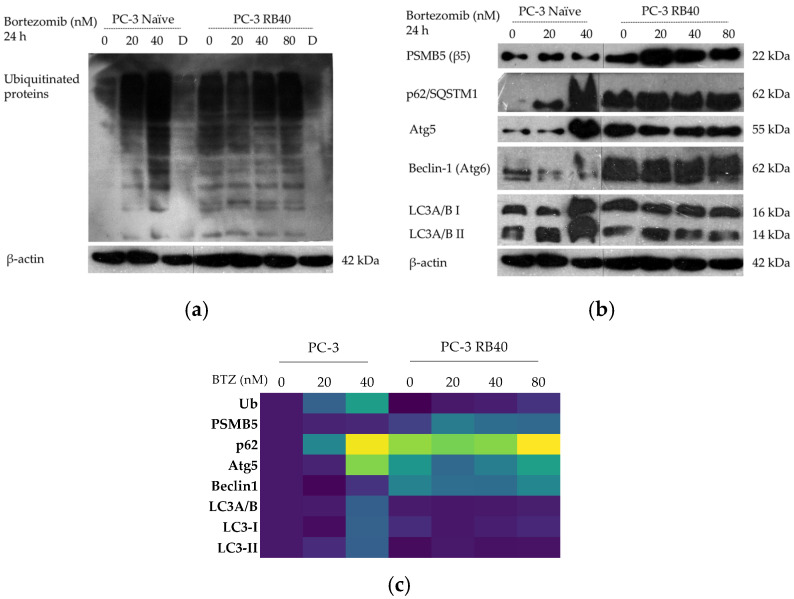
Western analysis of main UPS and autophagy proteins. Cells (naïve PC-3 and PC-3 RB40) were cultured inside 100 mm dishes, and 24 h before confluency, the media were changed, and fresh RPMI 1640 supplemented with 10% with or without the designated Bortezomib doses (20, 40, 80 nM) was added. (**a**) Western blot of ubiquitinated proteins. (**b**) Western blots of PSMB (β5), p62/SQSTM1, Atg, Beclin-1 (Atg6), and LC3A/B I and II. (**c**) Heatmaps depicting changes in protein expression/accumulation, following quantification using the plug-in “Gel Blots” in ImageJ.

Bortezomib interferes with the UPS, leading to the accumulation of polyubiquitinated polypeptides that cannot be degraded through cell mechanisms. Therefore, this accumulation can act as a marker for UPS activity. Western analysis made the drug-induced impairment evident in naïve cells, which was also shown to be dose-dependent (Figure 3a).

Resistant cells exhibited baseline ubiquitination levels, which could mean either de-sensitization to Bortezomib or exploitation of alternative pathways to substitute for the blocked proteasome. Bortezomib binds to the 20S proteasome’s β5 subunit, and in many Bortezomib-resistance cases, mutations or alterations in the subunit expression have been documented [26,27,62]. Additionally, in these experiments, both naïve and resistant cells were incubated with Doxorubicin (Figure 3a), which has been found to activate the UPS system [63]. Indeed, our experiments indicated decreased levels of ubiquitination in PC-3 RB40 cells, which was theorized as a marker of activated proteasome-mediated proteolysis (Figure 3a,c). Moreover, the β5 subunit (or PSMB5) was assessed using Western analysis and found to be significantly elevated in the resistant cells (Figure 3b and Figure S5a,b). This was theorized as a response to the proteasome’s inability to process the polyubiquitinated protein load, instead of a crucial aspect of Bortezomib resistance. Resistant cells were able to withstand overwhelming Bortezomib concentrations, which could not be surpassed by simply overexpressing PSMB5. For this reason, our interest was directed towards the activation of autophagy as an alternative response mechanism.

#### 3.2.2. Autophagy Assays

**Figure 4:**
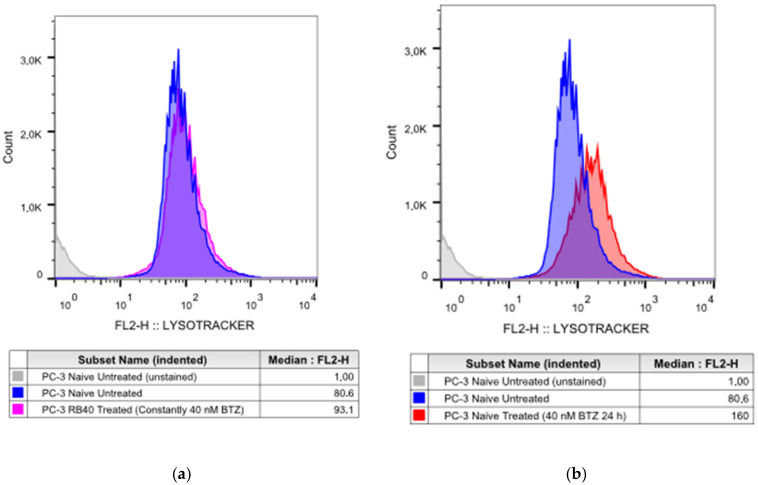

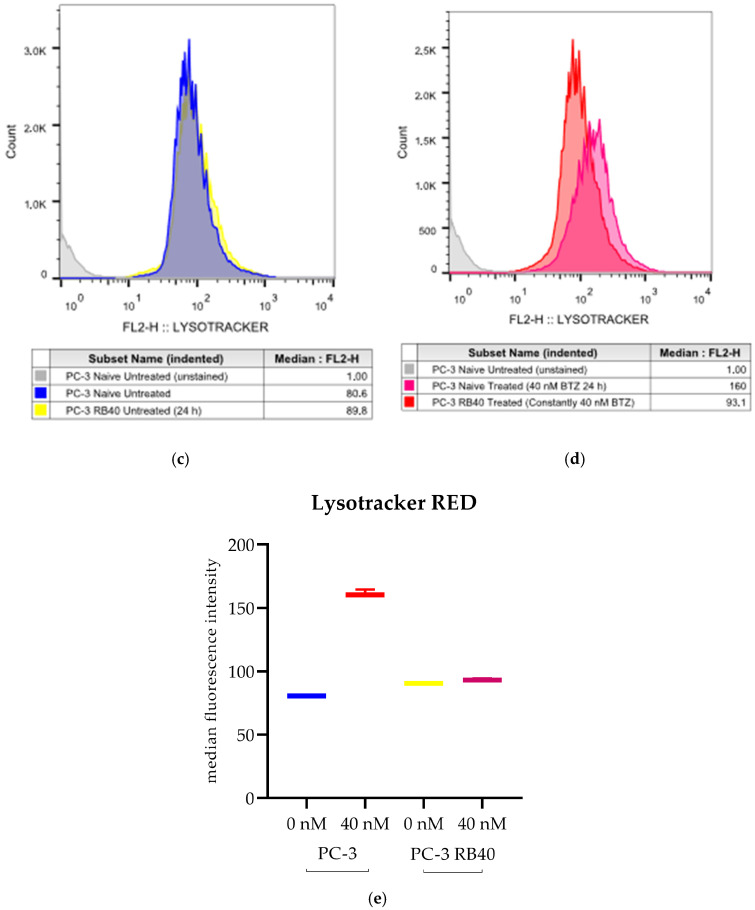
Autophagy assay using LysoTracker™ Red of (**a**) untreated naïve cells (blue histograms); (**b**) naïve PC-3 cells treated with 40 nM of Bortezomib for 24 h (red histograms); (**c**) PC-3 RB40 cells Bortezomib-deprived for 24 h (yellow histogram); and (**d**) PC-3 RB40 cells, continuously cultured with 40 nM of Bortezomib (magenta histograms). (**a**–**d**) All samples were stained with the LIVE/DEAD™ kit and with LysoTracker™ RED. Equal numbers of events were acquired using a FACS Calibur flow cytometer by measuring the fluorescence of the LIVE/DEAD™ stain and LysoTracker™ RED, and the data were analyzed using the FlowJo software (V10). The histograms display the median fluorescence intensity (MFI) of the LysoTracker™ RED channel. The figure presents a representative experiment. The same procedure was replicated three times. (**e**) Box plot including data from all experiments. Whiskers correspond to the minimum and maximum value within each group.

The notion that autophagy can substitute for dysregulated UPS has been documented in several studies on different cancer types, including prostate cancer [11,35,44,64]. To monitor autophagic activity, the lysosomal marker LysoTracker™ Red DND-99 was used. LysoTracker™ has been used as an autophagy marker, even though it does not directly assess autophagic flux but rather stains and thus quantifies acidic proteins [65,66,67]. The acidic protein content of a cell is correlated to the lysosomal load, and therefore, due to the organelles’ role in autophagy, LysoTracker™ can be used to study autophagic activity. Staining with LysoTracker™ revealed that the baseline autophagy flux of PC-3 RB40 cells (baseline conditions for these cells refers to a medium constantly containing 40 nM of Bortezomib) was significantly higher compared to untreated naïve PC-3 cells (*p*-value < 0.0001), (Figure 4a,e). Treatment with Bortezomib significantly increased autophagic flux in naïve cells (*p*-value < 0.0001), (Figure 4b,e). Nonetheless, resistant cells indicated only minor differences between untreated and treated (40 nM Bortezomib, 24 h) cells (*p*-value = 0.0802), (Figure 4c,e). Surprisingly, LysoTracker™ staining in treated PC-3 cells was almost double compared to treated PC-3 RB40 cells, indicating that the resistant clone has fine-tuned autophagy to substitute for the impaired UPS, while non-resistant cells uncontrollably upregulate autophagy and accumulate acidic proteins (*p*-value < 0.0001) (Figure 4d,e). All analyses were performed using one-way ANOVAs and Tukey’s multiple comparisons tests, and the statistical data can be found in Table A5.

Western analyses of key autophagy biomarkers and regulators were performed, focusing on p62/SQSTM1, Atg5, Beclin-1 (Atg6), and LC3A/B (Atg8) (Figure 3b,c) were also used. p62/SQSTM1 is a cargo protein that transfers polyubiquitinated proteins for degradation through autophagy, thus linking the two degradational pathways [68]. It interacts with LC3 II and the polyubiquitinated protein, leading to the protein’s degradation [69]. During autophagy, p62 is degraded as well; therefore, the elevated p62 can indicate either autophagic flux suppression or increased autophagy, as it has been shown that p62 overexpression (as a result of NF-κB activation) serves the induction of autophagy [70]. In the first case, other autophagic markers would be absent due to significant downregulation, while in the second case, increased p62 would be necessary to carry an increased load toward degradation. The role of p62 in Bortezomib resistance has only recently been uncovered, and a link between its expression and PI-resistance has been established [64]. In our experiments, p62 was found to be significantly increased in the naïve cells following Bortezomib administration, in a dose-dependent manner (Figure 3b,c and Figure S5c). In the resistant clone, regardless of the dose of Bortezomib applied, p62 remained relatively stable, with a three-fold greater baseline level than that of naïve cells (Figure S5c). This observation supports the exploitation of autophagy as an alternative proteostatic mechanism; however, other autophagic markers were assessed as well.

Atg5 was found elevated after incubation with Bortezomib in naïve cells and indicated a dose-dependent pattern of accumulation. The baseline accumulation of resistant cells is significantly greater than that of naïve cells. Incubation with 20 nM Bortezomib led to a slight downregulation in its accumulation, while the absence of Bortezomib or exceeding the baseline 40 nM dose led to its further accumulation (Figure 3b and Figure S5d). Atg5 accumulation was considered an important upregulation index, following both Bortezomib treatment and resistance emergence. The cytoprotective nature of autophagy during stress conditions was theorized to be the most plausible explanation [71,72].

A protein of high significance in the study of autophagy is Beclin-1/Atg6, which regulates the autophagy-apoptosis axis through interaction with the Bcl-2 antiapoptotic protein family [73,74]. Beclin-1 accumulation was observed to drop after treatment with a low dose of Bortezomib (20 nM). On the contrary, at the high, toxic dose of 40 nM, its accumulation increased, indicating the induction of autophagy due to severe UPS impairment. This was theorized to be a PI-induced cellular response to stress, which activates autophagy to promote survival under these conditions. In resistant cells, Beclin-1 maintains stable levels that are almost three-fold higher compared to the baseline expression in naïve cells. Sudden drug dose fluctuations seem to disrupt the equilibrium and induce stress effects on resistant cells, as Beclin-1 levels were documented to rise (Figure 3b and Figure S5e).

Finally, LC3A/B accumulation and conversion from LC3A/B I to LC3A/B II were assessed using Western blots (Figure 3b,c). Interpretation of LC3 accumulation comes with difficulties since its expression significantly increases during autophagy (LC3 I levels increase and, through conversion, LC3 II levels increase as well). However, the degradation of LC3 II inside the autophagosomes plays a significant role as well, thus decreasing (detectable) LC3 II. An accumulation of LC3 I can mean autophagic flux suppression, while an accumulation of LC3 II can be interpreted as autophagy induction [75]. Total LC3A/B was estimated as the sum of the two observed bands, one at ~14 kDa (LC3 II) and one at ~16 kDa (LC3 I). Following treatment with Bortezomib, the naïve cells exhibited a dose-dependent pattern of LC3 accumulation, because of the autophagic induction. LC3A/B increased more than two-fold following treatment with 40 nM of Bortezomib, while a clear upward trend was documented at the low dose of 20 nM (Figure 3a and Figure S5f). The two forms of total LC3 were also analyzed separately (Figure 3b,c and Figure S5g,h). In naïve cells, LC3 I decreased by ~40% following treatment with a low dose of Bortezomib (20 nM), while LC3 II increased its accumulation by ~40%. At the high Bortezomib dose, both forms were found to accumulate more than two-fold compared to the untreated group. The ratio of LC3 conversion (LC3A/B II: LC3A/B I) which is commonly used to estimate autophagy flux, exhibited a significant induction following the administration of 20 nM Bortezomib by almost 70% (indicating the UPS impairment), while the high dose returned the ratio to baseline levels (Table A6). Given the significant accumulation of both forms of LC3A/B, we did not theorize this restoration as an autophagy downregulation, but rather an increase in degradation capacity that led to rapid LC3 degradation before its levels exceeded a particular detectable level. In the resistant clone, LC3 A/B fluctuated to slightly higher values compared to naïve cells. The baseline accumulation of total LC3A/B (40 nM of Bortezomib) was slightly lower than that documented in naïve cells. Deviations from this dose (total absence or higher drug concentrations) resulted in slight increases in LC3A/B accumulation, while the administration of a lower drug dose slightly decreased LC3A/B. A separate analysis of LCA/B I indicated that its presence followed the total protein accumulation pattern, while the results differed for LC3A/B II. LC3A/B II in general followed the same pattern as total LC3; however, at the 20 nM bortezomib dose, the resistant cells did not diminish their LC3 II levels but rather increased them. This could mean a rapid acceleration of autophagic flux; however, this would be controversial regarding the lesser interference imposed by a lower Bortezomib dose. Therefore, the elevation of LC3A/B II was interpreted using the accumulation of p62 as an index (since they are believed to directly interact [69]), leading us to the conclusion that when the Bortezomib dose was diminished, the degradation capacity was tuned accordingly by downregulating p62, and as a result, the accumulation of p62 increased. This interpretation also explained why the ratios calculated in resistant cells were lower compared to the control values, while all other autophagy indices assessed (Lysotracker, Atg5, Beclin-1, p62) supported the notion of upregulated autophagy. With autophagy being the sole targeted-degradation mechanism given that the UPS function was compromised, the autophagic degradation rate increased.

### 3.3. Bortezomib-Resistant Cells Significantly Reduce Their Intracellular Reactive Oxygen Species and Downregulate Stress Signaling

#### 3.3.1. Oxidative Stress Level Determination

**Figure 5:**
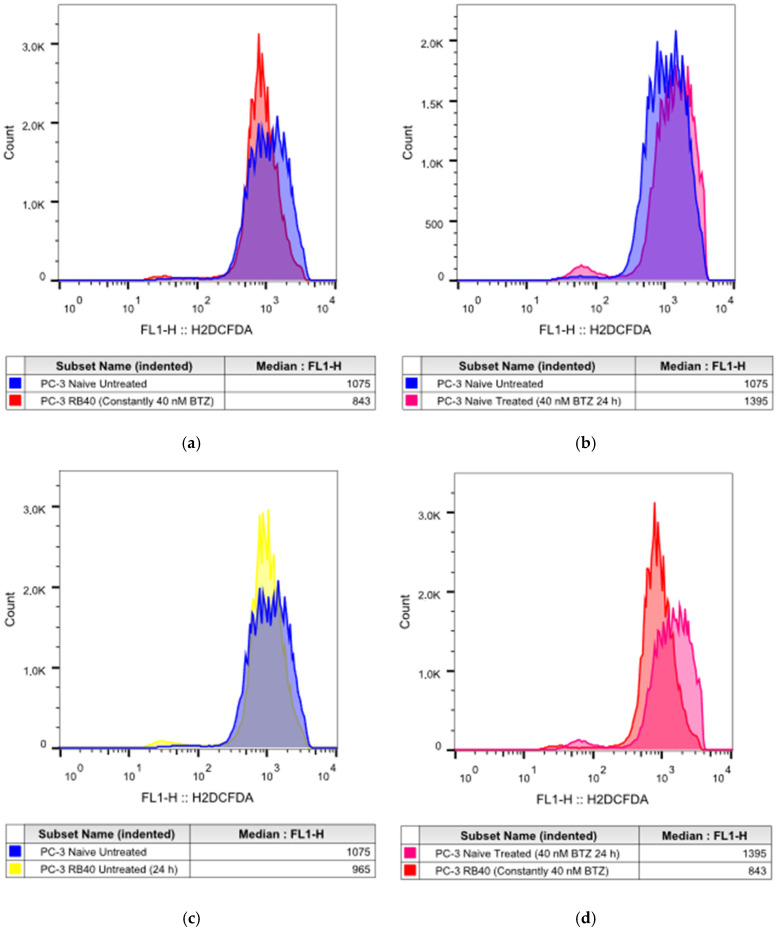

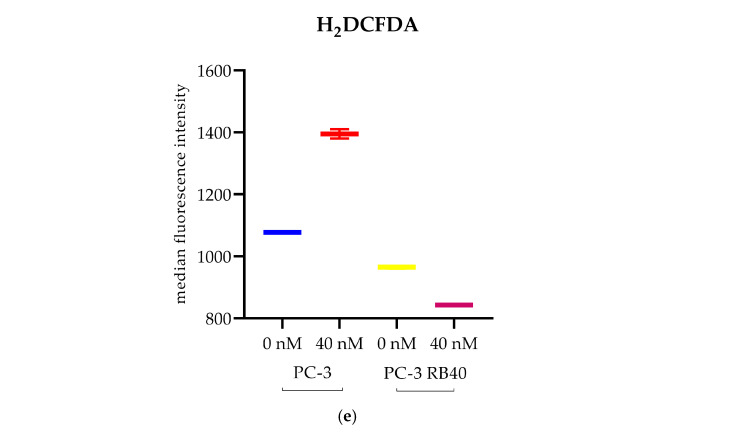
Intracellular reactive oxygen species (ROS) assay using H_2_DCFDA of (**a**) untreated naïve cells (blue histograms); (**b**) naïve PC-3 cells treated with 40 nM of Bortezomib for 24 h (red histograms); (**c**) PC-3 RB40 cells Bortezomib-deprived for 24 h (yellow histogram); and (**d**) PC-3 RB40 cells, continuously cultured with 40 nM of Bortezomib (magenta histograms). (**a**–**d**) All samples were stained with the LIVE/DEAD kit and with H_2_DCFDA. Equal numbers of events were acquired using a FACS Calibur flow cytometer by measuring the fluorescence of the LIVE/DEAD stain and H_2_DCFDA, and the data were analyzed using the FlowJo software. The histograms display the median fluorescence intensity (MFI) of the H_2_DCFDA channel. The figure presents a representative experiment. The same procedure was replicated three times. (**e**) Box plot including data from all experiments. Whiskers correspond to the minimum and maximum value within each group.

Given the importance of proteostasis in cell metabolism (through amino acid recycling, damaged protein degradation, and the role of ubiquitination in the regulation of certain pathways), a direct link between proteasome function, autophagic flux, and intracellular oxidative stress was speculated. Therefore, the cells were assessed using H_2_DCFDA to monitor the generation of intracellular reactive oxygen species (Figure 5) [53,54].

Baseline ROS levels of PC-3 RB40 resistant cells (constantly cultured with 40 nM Bortezomib) were significantly lower compared to both treated (*p*-value < 0.0001) and untreated PC-3 cells (*p*-value < 0.0001) (Figure 5a,c,e). This observation was made again by our research team in Zafeiropoulou et al. 2024 [11]. Herein, PI-resistant PC-3 cells demonstrated similar results as we observed the same phenomenon. Treatment with Bortezomib always elevates ROS levels (Figure 5b) in naïve cells, while the resistant clone is more stable regarding changes in its ROS levels (Figure 5d). Drug deprivation from PC-3 RB40 cells elevated their ROS levels during the first 24 h (*p*-value < 0.0001). All analyses were performed using one-way ANOVAs and Tukey’s multiple comparisons tests, and the statistical data can be found in Table A7. Specific time-course experiments showed that oxidative stress increases in both a dose- and a time-dependent manner in naïve cells, while resistant cells always had significantly lower levels (Table A8) (Figure S10). The overall ROS levels of PC-3 RB40 cells remain significantly lower compared to naïve cells, regardless of treatment or duration (*p*-value < 0.0001). Details on the time-course experiments on ROS accumulation are presented in Appendix E and Supplement S3 Section.

**Figure 6:**
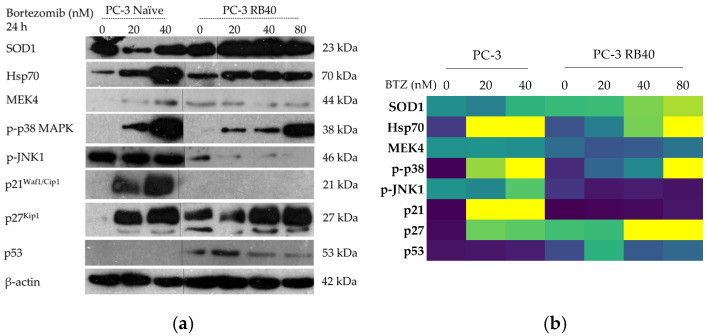
Western analysis of stress markers and cell cycle regulators. Cells (naïve PC-3 and PC-3 RB40) were cultured inside 100 mm dishes, and 24 h before confluency, the media were changed, and fresh RPMI 1640 supplemented with 10% with or without the designated Bortezomib doses (20, 40, 80 nM) was added. (**a**) Representative Western blots of SOD1, Hsp70, MEK4, p-p38 (MAPK11), p-JNK1 (MAPK8), p21^wif1/cip1^, p27^kip1^, and p53. (**b**) Heatmaps depicting changes in protein expression/accumulation, following quantification using the plug-in “Gel Blots” in ImageJ.

The lower ROS levels detected in RB40 cells were quite controversial given the impaired UPS. This led us to the conclusion that autophagy is responsible for recycling oxidatively damaged parts of the cells that normally undergo K48 labeling and proteasomal degradation. Moreover, the antioxidant enzyme superoxide dismutase 1 (SOD1) was also assessed due to its value as a marker [76]. Increased SOD1 would indicate a constant need for superoxide radical degradation, as its synthesis is regulated by redox signaling, and would mean an increased antioxidant capacity of the cell. The RB40-resistant clone exhibited increased SOD1 levels, almost doubling its accumulation as shown in the blots, compared to the naïve clone, in which baseline SOD1 expression was lower (Figure 6a,b and Figure S6a,b). Treatment with Bortezomib in both clones further increased the accumulation of SOD1, highlighting a correlation between redox signaling and Bortezomib that has not been fully described or uncovered.

#### 3.3.2. Stress Signaling Pathways

The stress levels inside resistant cells were also assessed, focusing on Hsp family proteins, p38 (MAPK11), and JNK1/SAPK1 (MAPK8) signaling. The molecular chaperon Hsp70 assists UPS-mediated degradation and its expression in stress conditions is elevated. Bortezomib is a known Hsp family inducer [11,77], and this was documented in the naïve clone, in which incubation with Bortezomib increased Hsp70 accumulation in a dose-dependent manner (Figure 6a,b and Figure S6c). Such an induction was not documented in the resistant clone. The PC-3 RB40 cells had baseline Hsp70 levels slightly greater than naïve PC-3 cells. Nevertheless, Hsp70 accumulation only indicated a trend for elevation after administration of 80 nM Bortezomib.

Other stress indicators that become activated after drug-induced stress are the MAPKs; p38 and JNK1 [78,79,80,81,82]. MEK4, which can phosphorylate both MAPKs, was assessed and found to be significantly downregulated in the resistant clone, both during drug absence and in the presence of various doses. Not even the highest Bortezomib concentration administered (80 nM) in RB40 cells was able to raise MEK4 accumulation to naïve-like levels, while in naïve cells, Bortezomib treatment resulted in MEK4 increases (Figure 6a,b and Figure S6d). Furthermore, p38 was found to be significantly phosphorylated after Bortezomib treatment in naïve cells following a dose–response manner, while the magnitude of the phenomenon was documented in the resistant cells and was several times smaller (Figure 6a,b and Figure S6e). In general, the resistant cells maintained low but detectable levels of p-p38 MAPK. However, it is noteworthy that the phosphorylation observed in the naïve clone was far greater than that seen during the treatment of RB40 cells with 80 nM of Bortezomib (Figure S6e). The other major stress-related MAPK, JNK1/SAPK1, was assessed and found to follow a similar downregulation pattern in the resistant cells. In naïve cells, upon treatment with 20 nM of Bortezomib, p-JNK1 initially decreased. On the contrary, dose elevation to 40 nM led to an increase of about 50% compared to the untreated clone. The resistant clone was documented to have decreased p-JNK1 phosphorylation by about 90%, a characteristic quite uniform at different doses and even drug absence (Figure 6a,b and Figure S6f). These results indicated that Bortezomib, despite its action that renders PSMB5 non-functional upon binding, cannot successfully induce the signal transduction in stress-related pathways in the resistant cells, as they maintain low levels of the kinases (or the phosphorylated form of them) that can lead to apoptosis.

#### 3.3.3. Cell Cycle Regulators (p21, p27) and p53

One of the main effects of Bortezomib is the cell cycle arrest in the G_1_/S and G_2_/M phases, which is mediated through the accumulation of cell cycle regulators [83]. Other studies have highlighted the accumulation of p21 and p27 after Bortezomib treatment (20–40 nM), which was evident in the naïve clone studied in this study as well. The resistant clone downregulated p21accumulation to baseline levels. Then, p21 peaked again only when the Bortezomib dose escalated to 80 nM (Figure 6a,b and Figure S6g). Regarding p27 accumulation, it was found increased following Bortezomib treatment in naïve cells, and to a lesser point, it was also present in resistant cells (Figure 6a,b and Figure S6h). Alongside these, an important cell cycle regulator is the p53 protein, which acts as a tumor suppressor gene. Over-expression or accumulation of p53 usually inhibits cell cycle progression; however, this was not observed in the resistant cells of this study. Naïve cells accumulated p53 after Bortezomib administration, which was four-fold lower compared to p53 levels observed in RB40 cells (Figure 6a,b and Figure S6i). The presence of p53 could promote DNA repair and protect the genome from the genotoxic effects of Bortezomib; nonetheless, its accumulation did not cause cell cycle arrest.

### 3.4. Resistant Cells Are More Aggressive and Express Epithelial to Mesenchymal Transition (EMT) Markers

Besides the known effects on cell proliferation and apoptosis, Bortezomib has been documented to inhibit cell adhesion and migration. This is believed to be a result of its interference with the turnover times of various molecules, which causes disruptions in adhesome functionality [84,85,86]. Ubiquitin, per se, also serves as a signaling molecule, controlling Wnt signaling through stabilization or selective degradation. Therefore, the consequences of UPS impairment on cell migration, adhesion, and related molecules were carefully studied [87,88,89,90].

#### 3.4.1. Wound Healing Ability Assays

**Figure 7:**
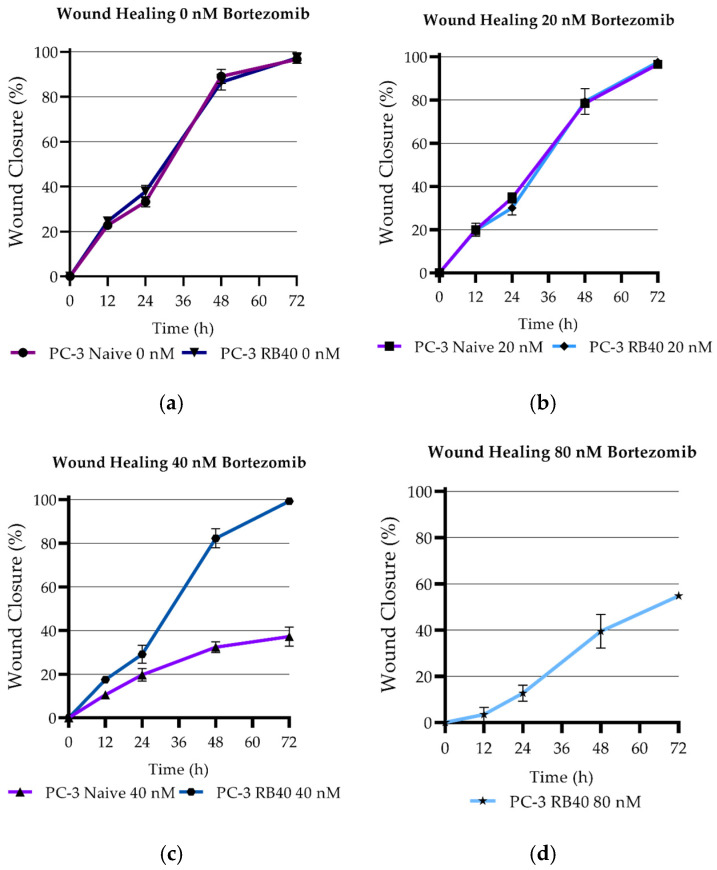
Scratch test/wound healing assay of (**a**) naïve PC-3 and PC-3 RB40 cells untreated for 72h; (**b**) naïve and PC-3 RB40 cells, both treated with 20 nM Bortezomib; (**c**) naïve and PC-3 RB40 cells, both treated with 40 nM Bortezomib; and (**d**) RB40 cells treated with 80 nM Bortezomib. Each wound healing experiment was conducted in triplicate, and the values on the plots are the averages. The error bars correspond to the standard deviation (SD) from the three experiments.

To observe differences in the cells’ ability to successfully heal artificial wounds by dividing and migrating, both naïve and resistant cells (PC-3 RB40) were assessed, using scratch test assays (Table A9). Dose–response experiments showed that treatment with 40 nM Bortezomib significantly inhibited wound healing in naïve cells (*p*-value < 0.0001) (Figure 7a–c). On the other hand, the RB40 clone was not affected by the inhibitor (*p*-value = 0.0948), indicating a significant inhibition only at concentrations greater than 40 nM (*p*-value = 0.0070), (Figure 7c,d).

**Figure A2:**
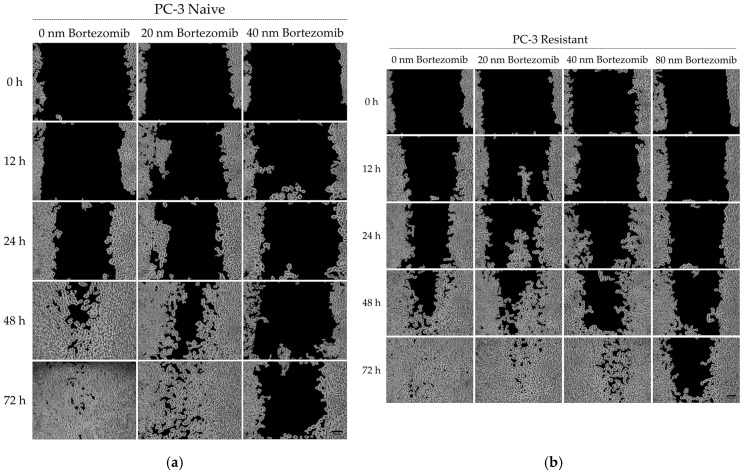
Scratch test/wound healing assay of (**a**) naïve PC-3; and (**b**) PC-3 RB40 cells. Photographs were taken at the key time points of 0, 24, 48, and 72 h using a camera mounted on an inverted microscope at 100× magnification.

The scratch assays lasted 72 h, and at this point, the untreated naïve cells as well as the RB40 clone (untreated and treated with 20 or 40 nM Bortezomib) managed to fully heal the scratches. Full images of a representative wound healing experiment and the corresponding analysis can be found in Appendix F.1 (Figure A2a,b).

#### 3.4.2. Migration Assays

**Figure 8:**
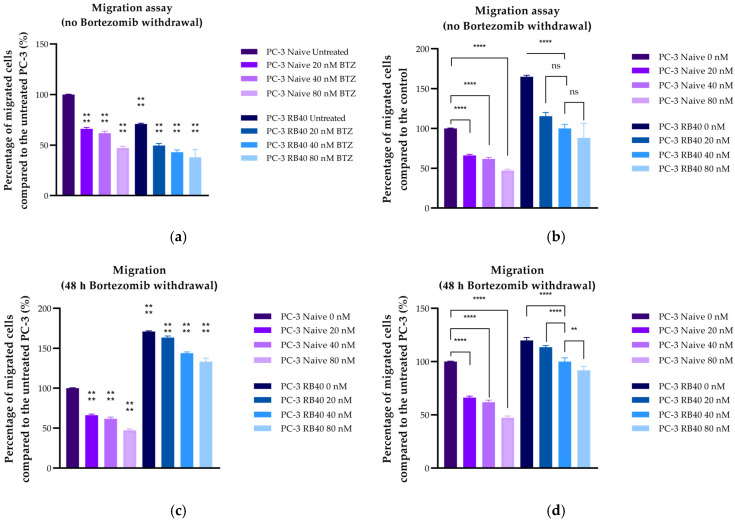
Migration assay using increasing concentrations of Bortezomib (0, 20, 40, and 80 nM for 24 h. (**a**) The percentage of migrated cells was compared to the naïve untreated sample; (**b**) the percentages of total migrated cells, compared to each clone’s baseline conditions (untreated for the naïve cells, and 40 nM Bortezomib for the RB40 cells); (**c**,**d**) the migration experiments were also conducted following a 48 h Bortezomib withdrawal/clearance period of the RB40 clone. The results were analyzed using multiple comparisons of one-way ANOVA in the Prism 8 software. “ns” corresponds to non-significant; ** corresponds to a *p*-value = 0.001; and **** corresponds to a *p*-value < 0.0001). The error bars represent the standard error of the mean (SEM).

The migratory and chemorepellent effects of Bortezomib on both naïve and resistant cells were subsequently assessed using Transwell chambers (Figure 8a–d). Bortezomib exhibited significantly suppressive effects on the cells’ ability to migrate toward the chemoattractant medium, as indicated by experiments where the drug was placed in the insert with a serum-free medium (*p*-value < 0.0001) (Figure 8a,b). The same effect was also observed in resistant cells, whose migratory abilities were severely impaired in the presence of Bortezomib, compared to cells cultured without the inhibitor (*p*-value = 0.0007). Surprisingly, the resistant cell’s ability to migrate even in drug absence was lower than that of naïve cells (*p*-value < 0.0001). However, the ability of untreated RB40 cells to migrate was greater than that of low-dose-treated naïve cells (*p*-value < 0.0001). These two conditions may seem different; however, given that the resistant cells were not given a Bortezomib clearance period from their previous maintenance with 40 nM Bortezomib, the decreased levels of migration could be a consequence of the residual drug.

**Figure A3:**
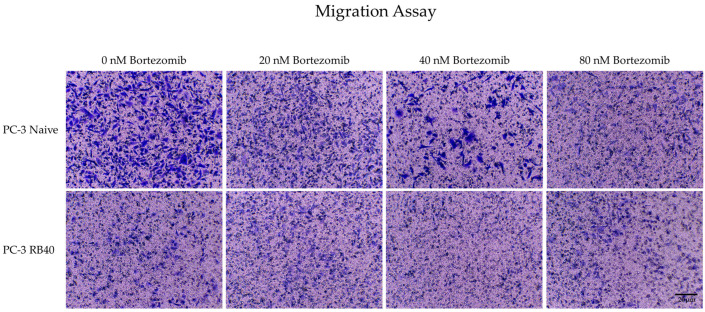
Migration assay using Boyden chambers. Equal numbers of cells were placed in Transwell/Boyden Chambers in serum-free RPMI 1640 medium containing increasing concentrations of Bortezomib (0, 20, 40, and 80 nM), and the inserts were placed in microwells containing FBS-supplemented medium. The cells were left for 24 h to migrate, and afterwards, the cells crossing the filters were fixed and stained with crystal violet. Photographs were taken using a 10× objective and the fixed cells were counted using the multipoint tool by ImageJ. Photographs were taken using a 10× objective, and the fixed cells were counted using the multipoint tool by ImageJ.

Therefore, we repeated the experiments by adding a Bortezomib clearance period of 48 h prior to the assays. The results indicated that the RB40 cell line was significantly more aggressive compared to naïve cells (*p*-value < 0.0001) (Figure 8c,d). Bortezomib (both in drug-deprived and treated-resistant cells) acted in a dose-dependent manner. Nonetheless, in the case of drug-deprived cells, even 80 nM of Bortezomib did not diminish the cell’s migratory potential to naïve levels (*p*-value = 0.2856). Full images of representative migration assays can be found in Appendix F.2 (Figure A3).

#### 3.4.3. Chemotaxis Assays

**Figure 9:**
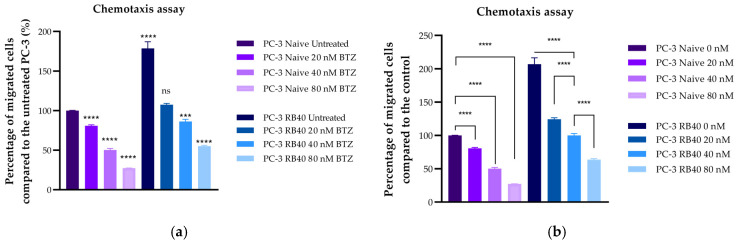
Chemotaxis Assay. Equal numbers of cells were placed in Transwell/Boyden Chambers in serum-free RPMI 1640 medium, and the inserts were placed in microwells with FBS-supplemented medium containing increasing concentrations of Bortezomib (0, 20, 40, and 80 nM) for 24 h. (**a**) The percentage of migrated cells was compared to the naïve untreated sample; and (**b**) the percentages of total migrated cells, compared to each clone’s baseline conditions (untreated for the naïve cells, and 40 nM Bortezomib for the RB40 cells). The results were analyzed using multiple comparisons of one-way ANOVA in the Prism 8 software (“ns” corresponds to non-significant, *** corresponds to a *p*-value = 0.0001; and **** corresponds to a *p*-value < 0.0001). The error bars represent the standard error of the mean (SEM).

**Figure A4:**
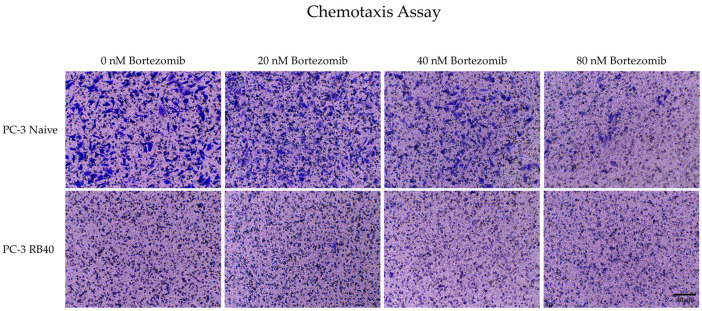
Chemotaxis assay using Boyden chambers. Equal numbers of cells were placed in Transwell/Boyden Chambers in serum-free RPMI 1640 medium, and the inserts were placed in microwells with FBS-supplemented medium containing increasing concentrations of Bortezomib (0, 20, 40, and 80 nM). The cells were left for 24 h to migrate, and afterwards, the cells crossing the filters were fixed and stained with crystal violet. Photographs were taken using a 10× objective, and the fixed cells were counted using the multipoint tool by ImageJ.

Bortezomib was also examined as a chemoattractant/chemorepellent agent by adding it to the lower compartment (microplate well) along with serum at a 10% concentration (Figure 9a,b). It was observed to successfully repel naïve PC-3 cells and mask the chemoattractant medium’s presence. However, resistant cells were not affected by the drug’s presence at the same doses. Notably, the baseline cell motility levels for both cell clones (in treated naïve cells and resistant cells treated with 40 nM) were almost identical, indicating that the resistant cells can bypass the presence of Bortezomib and successfully migrate towards the chemoattractant medium. Full images of representative migration and chemotaxis assays can be found in Appendix F.3 (Figure A4).

#### 3.4.4. Cell Adhesion and Migration Signaling Pathways

**Figure 10:**
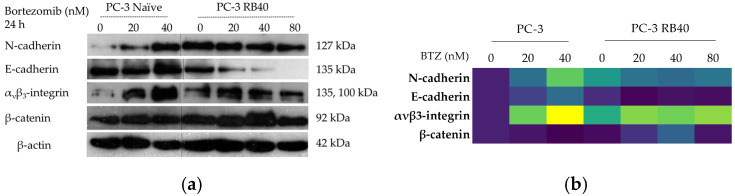
Western analysis of EMT markers. Cells (naïve PC-3 and PC-3 RB40) were cultured inside 100 mm dishes, and 24 h before confluency, the media were changed, and fresh RPMI 1640 supplemented with 10% with or without the designated Bortezomib doses (20, 40, 80 nM) was added. (**a**) Representative Western blots of N-Cadherin, E-cadherin, α_ν_β_3_-integrin, and β-catenin. (**b**) Heatmaps depicting changes in protein expression/accumulation, following quantification using the plug-in “Gel Blots” in ImageJ.

The resistant cells’ ability to defy the anti-migratory effects of Bortezomib regarding cell motility and migration led to the conclusion that proteins related to those functions could have been affected. Therefore, basic cadherins, α_ν_β_3_-integrin, and β-catenin were assayed using Western analysis. N-cadherin and E-cadherin are two of the most important calcium-dependent cell adhesion molecules, both participating in a phenomenon called cadherin switch. During cadherin switch, N-cadherin is upregulated and E-cadherin is downregulated, both driving an aggressive phenotype [91,92]. The resistant clone emerged, significantly increasing the accumulation of N-cadherin compared to the baseline expression observed in naïve cells (Figure 10a,b and Figure S7a,b). Treatment with Bortezomib increased N-cadherin accumulation both in naïve and resistant cells; however, the resistant cells had a more uniform pattern of expression, regardless of the drug’s presence. The opposite phenomenon was documented regarding E-cadherin. Following treatment with 20 nM of Bortezomib, E-cadherin indicated reduced accumulation; however, a higher dose of Bortezomib (40 nM) led to a significant decrease in its presence (Figure 10a,b and Figure S7c). Cadherin switch was evident in the resistant clone, since E-cadherin had significantly lowered accumulation levels compared to the (naïve) untreated cells. Additionally, E-cadherin accumulation did not fluctuate in this clone, regardless of the inhibitor dose, showing an abolishment of ubiquitin regulation (Figure S7c).

The accumulation of α_ν_β_3_-integrin was also assessed and indicated a dose-dependent accumulation in naïve cells that reached a six-fold increase compared to the control sample (Figure 5a,b and Figure S7d). Integrin levels in resistant cells remained higher (three-fold), even when the drug was absent, and during Bortezomib treatment (20–80 nM), the protein levels were not affected. Furthermore, α_ν_β_3_-integrin is one of the most studied adhesion molecules in prostate cancer, having been reported as essential for extracellular matrix adhesion during invasion and metastasis [93], and not only interacts with the actin cytoskeleton and the related scaffold proteins, but also regulates survival and drives metastasis-related genes by clustering and acting as recruitment areas where phosphorylation of FAK can take place. These signals are finally transmitted inside the nucleus through the ERK1/2 pathway, which in our experiments was found to be activated, as will be discussed subsequently. Besides ERK1/2 signaling, α_ν_β_3_-integrin also regulates MMP activity through PI3K signaling [93]. Given that PI3K-Akt signaling was found to be upregulated, as will be discussed later, the role of α_ν_β_3_-integrin in the resistant cells’ aggressive phenotype regarding migration was adequately explained.

Finally, the transcription regulator β-catenin also serves as a connecting link between the UPS and adhesion. β-catenin is a molecule that has been repeatedly correlated with cancer invasiveness and EMT [94]. The resistant clones exhibited increased β-catenin accumulation, an observation in accordance with the protein’s role as an oncogene [95]. Following treatment with Bortezomib, the naïve cells decreased the accumulation of the protein; however, in resistant cells, the baseline levels (at 40 nM of Bortezomib) increased by 50% compared to the untreated naïve cells. Deviation from this drug concentration was found to affect β-catenin expression negatively; however, the total accumulation ranged between values greater than those observed in the naïve clone (Figure 5a,b and Figure S7e).

### 3.5. Resistant Cells Activate Proliferation-Related Signaling Through STAT3, NF-κβ, and cJun and Downregulate STAT1

#### 3.5.1. JAK-STAT Signaling Pathway

**Figure 11:**
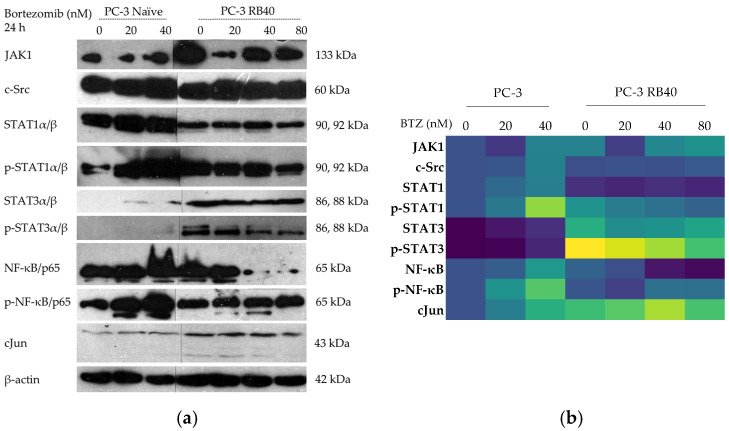
Western analysis of JAK-STAT, NF-κβ, and cJun pathways’ main proteins. Cells (naïve PC-3 and PC-3 RB40) were cultured inside 100 mm dishes, and 24 h before confluency, the media were changed, and fresh RPMI 1640 supplemented with 10% with or without the designated Bortezomib doses (20, 40, 80 nM) was added. (**a**) Representative Western blots of JAK1, c-Src, STAT1, p-STAT1, STAT3, p-STAT3, NF-κΒ, NF-κΒ, cJun. (**b**) Heatmaps depicting changes in protein expression/accumulation, following quantification using the plug-in “Gel Blots” in ImageJ.

STATs, cJun, and ΝF-κβ, are known to mediate rapid-acting actions in cancer cells, existing constantly in a deactivated state and participating in the gene transcription immediately upon phosphorylation. JAKs constitute a kinase family that transmits signals from cytokines and growth factors to STATs. JAK1 was assessed and revealed to increase its accumulation at high Bortezomib doses in both naïve and resistant cells (Figure 11a,b and Figure S8a,b,k). The lowest JAK1 accumulation was documented both in naïve and resistant cells following treatment with 20 nM of Bortezomib. The absence of Bortezomib led to a double JAK1 accumulation in the PC-3 RB40 cells, with almost identical accumulation levels documented at the 40 and 80 nM doses. Src kinases are non-receptor tyrosine kinases able to phosphorylate STATs and practically function as oncogenes. To study STATs activation, besides JAK1, the levels of the kinase c-Src were also assessed and found to be similar between naïve and resistant cells (Figure 11a,b and Figure S8c). The sole exception was a peak in c-Src accumulation observed in naïve cells during treatment with 40 nM of Bortezomib. However, this effect was not observed in the resistant cells.

STATs are a group of transcription factors that are very important during cytokine signaling and are detected activated in many cancer types. STAT1 and STAT3 have opposing biological actions regarding the regulation of gene expression. STAT1 induces pro-apoptotic and anti-proliferative genes, while STAT3 has pro-proliferative, anti-apoptotic, and pro-angiogenetic actions. Loss or downregulation of STAT1 is correlated with a poor prognosis [96], while induction of STAT3 activity also promotes the aggressiveness of the tumor [97]. Regarding STAT1, incubation with Bortezomib led to a significant increase in its accumulation inside naïve cells that followed a dose-dependent pattern, acting as a precursor of apoptosis (Figure 11a,b and Figure S8d). In the resistant cells, STAT1 was significantly downregulated (more than 50%). The phosphorylation levels of STAT1 rapidly arose in naïve cells following treatment with Bortezomib in a dose-dependent manner and reached a three-fold change in its accumulation following treatment with 40 nM of Bortezomib (Figure 11a,b and Figure S8e). In the resistant cells, the baseline levels of phosphorylated STAT1 were upregulated by 50–80%, compared to naïve cells; however, the p-STAT1 levels never reached as high as those observed in treated naïve cells. On the contrary, STAT3 was found to be significantly upregulated in the resistant clone compared to naïve cells (in which STAT3 was not found at detectable levels) (Figure 11a,b and Figure S8f). After treatment with Bortezomib, the naïve clone accumulated STAT3; however, this phenomenon was 1000-fold lower than that observed in resistant cells, where the overexpression of STAT3 was evident and stable throughout different Bortezomib doses. Regarding STAT3 phosphorylation, the naïve cells mildly phosphorylated STAT3 after Bortezomib administration (compared to the untreated cells, where phosphorylation was undetectable), while the resistant cells constantly maintained fluctuating portions of phosphorylated STAT3 (Figure 11a,b and Figure S8g). During drug deprivation (for 24 h prior to the analysis), the resistant cells exhibited the highest levels of STAT3 phosphorylation, while the lowest values were observed at the basal conditions of 40 nM Bortezomib. Dose elevation increased the amount of phosphorylated STAT3, and so did the lower doses. Therefore, we concluded that PC-3 RB40 cells had adapted to the 40 nM bortezomib presence, leading to a reduced need for STAT3 signaling compared to fluctuations in this dose, which should be considered as stress-inducing conditions (both increases and decreases in the dose). In general, once compared to the naïve clone, the levels of phosphorylation in the PC-3 RB40 cells were two- to six-fold higher, highlighting the importance of STAT3 and its phosphorylation in achieving drug tolerance.

#### 3.5.2. NF-κΒ and cJun Activation

Nf-κB is a well-established transcription factor in Bortezomib resistance, being one of the older studied [98,99,100]. In naïve cells, treatment with Bortezomib induced both the accumulation of Nf-κB and phosphorylation in a dose-dependent manner (Figure 11a,b and Figure S8h). In PC-3 RB40 cells, the accumulation in the absence of Bortezomib was comparable to that of naïve cells (although slightly lower); however, in the presence of Bortezomib, its accumulation rose to high levels. Regarding phosphorylation, in the resistant cells, during incubation with 40 nM (baseline conditions for these cells) and 80 nM Bortezomib, Nf-κB exhibited maximum activation, while complete drug withdrawal or dose reductions led to decreases (Figure 11a,b and Figure S8i). In general, the activated fraction of Nf-κB in the resistant cells was significantly lower than that of naïve cells; however, the total Nf-κB levels were circa two times higher compared to those of the naïve clone.

cJun is a transcription factor activated in stress conditions as a result of exposure to UV radiation, reactive oxygen species elevation, and/or drug-induced stress. Following Bortezomib incubation, cancer cells have been shown to accumulate cJun, which is believed to mediate apoptosis [82]. Upon incubation with Bortezomib, the naïve cells accumulated cJun in a dose-dependent manner. Notably, the accumulation in the resistant clone was far greater, and cJun was maintained at relatively uniform levels regardless of Bortezomib dose (even during complete absence) (Figure 11a,b and Figure S8j).

### 3.6. Resistant Cells Activate PI3K-Akt and MAPK/ERK Pathways, and Upregulate Elk1-Mediated Gene Transcription

#### 3.6.1. PI3K-Akt Pathway

**Figure 12:**
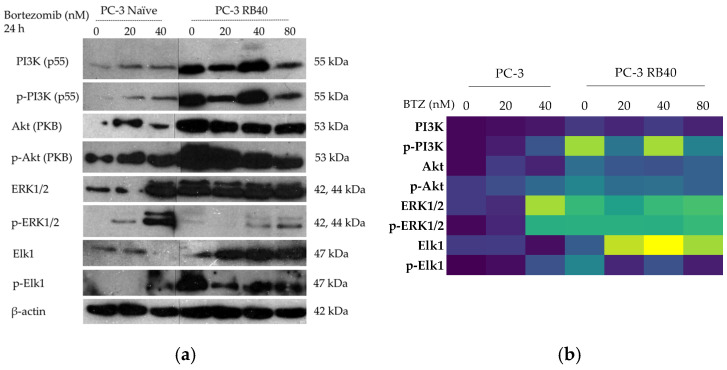
Western analysis of the PI3K-Akt and ERK1/2-Elk1 pathways. Cells (naïve PC-3 and PC-3 RB40) were cultured inside 100 mm dishes, and 24 h before confluency, the media were changed, and fresh RPMI 1640 supplemented with 10% with or without the designated Bortezomib doses (20, 40, 80 nM) was added. (**a**) Representative Western blots of PI3K (p85/55), p-PI3K (p-p85/55), Akt (PKB), p-Akt (p-PKB), ERK1/2 (MAPK3/1), p-ERK1/2 (p-MAPK3/1), Elk1, p-Elk1. (**b**) Heatmaps depicting changes in protein expression/accumulation, following quantification using the plug-in “Gel Blots” in ImageJ.

Besides STATs and NF-κΒ signaling, the main signaling pathways involved in Bortezomib resistance are the Ras-Raf-MEK-MAPK, and the PI3K-Akt pathways. Both control multiple cellular functions including viability, proliferation, and the cell metabolism [101,102,103,104]. The main kinases were analyzed using Western analysis following 24 h of Bortezomib treatment with increasing doses. The cells were assessed at the 24 h key point, as it was found to be the interval in which Bortezomib actions were most evident, based on both other publications and preliminary results of our laboratory. First, the activity of the PI3K/Akt pathway was assessed by monitoring the two kinases’ accumulation and phosphorylation. This pathway regulates and promotes cell survival, induces autophagy as an anti-apoptotic mechanism, and transmits signals that come from upstream kinases, adhesion molecules, and other receptors [105]. PI3K (p85/55) was more abundant in naïve cells, following a dose-dependent pattern of accumulation (Figure 12a,b and Figure S9a,b,j). The resistant cells exhibited increased PI3K levels at baseline levels (40 nM of Bortezomib), which were relatively stable. A reduction was observed in the low dose of 20 nM; however, during drug absence or high Bortezomib concentrations, the accumulation of PI3K was amplified. The phosphorylation patterns were analogous to total accumulation (Figure 12a,b and Figure S9c). PI3K was activated in resistant cells, demonstrating a peak at the 40 nM dose. This indicated activation of the kinase to mediate the appropriate tuning of survival pathways. Downstream of PI3K, the protein kinase B (PKB), or Akt, was also studied.

In prostate cancer, Akt activation is a marker of poor prognosis [106], and the PC-3 cell line was selected as a model of an already dysregulated Akt activity. This happens because the phosphatase PTEN that suppresses Akt activation is absent in PC-3 cells due to a double deletion [107]. Normally, the presence of PTEN causes cell cycle arrest and subsequently apoptosis; nevertheless, the deletion of the tumor-suppressor gene *PTEN* renders PC-3 highly aggressive and more eager to develop drug resistance [108]. Akt was found to be significantly upregulated in resistant cells, fortifying the notion that signals from multiple kinases indeed reach Akt and are transmitted through it (Figure 12a,b and Figure S9d). Following treatment with Bortezomib, the naïve cells initially increase their Akt accumulation (20 nM of Bortezomib), while greater doses reduce the initial augmentation. In the resistant clone, the baseline levels are higher than those observed in naïve cells, and any deviation (positive or negative) from the concentration that the cells are adapted to (40 nM) causes Akt levels to increase. This was interpreted as the cells’ response to the stress imposed by the new drug concentration that dysregulates the achieved equilibrium. We also assessed Akt activation (phosphorylation), which also validated the increased activity of the pathway (Figure 12a,b and Figure S9e). Upon treatment with Bortezomib, naïve cells increased the Akt phosphorylation in a dose–response manner. Resistant cells exhibited a double accumulation of p-Akt compared to the naïve cells, and its accumulation slightly decreased as the drug dose increased. Since Akt is a pivotal molecule and substrate of many intracellular kinases, it becomes evident that it acts as a key signal transducer (with the second being the pair of ERK1/2) in pro-survival signaling. Akt mediates intracellular communication regarding the proteostatic capacity, regulates gene expression, and controls cell cycle progression (by overcoming the G_1_/S and G_2_/M arrest that can be caused by Bortezomib).

#### 3.6.2. ERK1/2-Elk1 Signaling Pathway

Regarding the Ras-Raf-MEK-MAPK pathway, the accumulation of ERK1/2 (MAPK3/1) was assessed. Being the foremost downstream kinases that can enter the nucleus and alter gene expression in favor of cell survival, proliferation, and regulation of apoptosis, their role is crucial for cell survival and are reportedly key contributors to aggressive phenotypes. The accumulation of ERK1/2 exhibited a five-fold increase in naïve cells after treatment with 40 nM Bortezomib, while lower doses (20 nM) did not alter ERK1/2 levels (Figure 12a,b and Figure S9f). In PC-3 RB40 cells, the baseline levels of ERK1/2 were four-fold higher compared to the control. Fluctuations of minor significance were observed, with a decrease in ERK1/2 accumulation after administration of 20 nM Bortezomib and a slight elevation upon treatment with 80 nM of the drug. The phosphorylation pattern differed from that of the total protein accumulation. The resistant cells were documented with a ten-fold change in ERK1/2 phosphorylation (Figure 12a,b and Figure S9g). On the other hand, naïve cells increased their p-ERK levels 90 times upon treatment with a high dose of Bortezomib (40 nM), while the low dose of 20 nM increased the phosphorylation levels only by six times. In PC-3 RB40 cells, the 80 nM Bortezomib dose increased the (already high) phosphorylation levels, doubling the amount observed in baseline conditions (40 nM).

Elk-1 is a transcription factor whose role in Bortezomib resistance has not been thoroughly studied [109]. Upon treatment with Bortezomib, naïve PC-3 cells downregulated the accumulation of total Elk1 at the 40 nM dose, while Elk1 accumulation at the 20 nM dose remained relatively similar to untreated cells (Figure 12a,b and Figure S9h). Regarding the transcription factor’s phosphorylation, in naïve cells, the low dose of Bortezomib (20 nM) led to a 15-fold induction, while the high dose (40 nM) increased the abundance of the phosphorylated fraction even more (Figure 12a,b and Figure S9i). In resistant cells, Elk1 was observed in significantly greater quantities. In the absence of Bortezomib, the cells accumulated almost twice the amount of protein found in naïve cells, while incubation with Bortezomib significantly increased Elk1 presence. Elk1 accumulation peaked at the baseline dose of 40 nM, while fluctuations in Bortezomib dose led to decreases in its abundance. The ratio of phosphorylated to total Elk1 remained stable during treatment with various Bortezomib doses, with peaks being observed in the absence of the drug and the baseline dose of 40 nM.

## 4. Discussion

**Figure 13:**
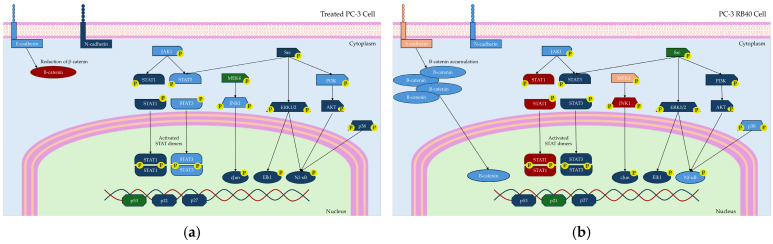
Main signaling pathway activation in (**a**) treated naïve PC-3, and (**b**) Bortezomib-resistant PC-3 RB40 Cells. The green color is used whenever the activation levels (phosphorylation/accumulation) remain similar compared to the baseline conditions (untreated naïve PC-3 cells). The red color is used to annotate downregulation: light red is used for mild changes and dark red for pronounced decreases. The blue color is used to annotate upregulation: light blue is used for mild changes and dark blue for pronounced increases. The diagrams are based on the western blot data gathered from the assays. By using arrows, all known (major) phosphorylation routes are indicated. The localization of each protein is also annotated, as well as events of dimerization, transport inside the nucleus, and DNA binding (for transcription factors and cell cycle regulators).

In this study, a Bortezomib-resistant prostate cancer cell line was created, and a broad-spectrum signaling investigation was performed by examining main signal transduction pathways, transcription factors, and stress levels. Our model, the PC-3 cell line, has already been used as a Bortezomib-resistant cell line [10,110], as it can acquire resistance to proteasome inhibitors after prolonged exposure. Given the limited efficacy of Bortezomib against solid tumors (including PCa), an attempt to gather evidence on signaling modulations as a way to improve our targeting against drug-resistant tumors and expand our biomarker repertoire. The resulting cell line was assessed for the restoration of its basic biological actions like cell viability, cell proliferation rate, the ability to surpass cell cycle arrest, and changes regarding the cell’s motility and metastatic potential. In addition, the resistant cells were documented to exhibit several EMT characteristics, with the most evident being cadherin switch and β-catenin upregulation. This observation explains the increased migratory potential of resistant cells, as well as the reinforced signal transduction through pathways correlated to cell adhesion signaling (MAPK, Akt, and Wnt signaling). The resistant cells were able to downregulate the accumulation of p21, override the inhibitory actions of p27, and avoid the cell cycle arrest caused by p53. All three proteins are regulated through ubiquitination, and their turnover rate is heavily impaired in treated naïve cells, leading to significant cell cycle arrest in the G_1_ phase. However, the resistant cells were able to bypass those checkpoints and not exit the cell cycle, an effect that was considered the consequence of viability pathways’ activation. More specifically, the PI3K-Akt pathway is the main survival pathway of the cells (able to overcome cytostatic signals), and in the resistant clone, it was found to be significantly activated, thus explaining successful cell cycle progression (Figure 13).

The activation of Akt can be catalyzed by PI3K, which was found to be both overexpressed and activated in the resistant clone, as well as through crosstalk with other kinases. Akt per se is known to be able to override cell cycle arrest signals as well as enhance cytoprotective functions like autophagy [111,112,113]. The same actions are believed to be mediated by the NF-κB pathway, which was also found activated in the resistant cells [114]. NF-κB signaling is one of the most typical signaling modulation mechanisms, found in Bortezomib-resistant cells. It was initially observed in hematological malignancies where NF-κB is inherently active due to its participation in inflammatory reactions [37,98,100,115]. In our case, the PCa Bortezomib-resistant cell line PC-3 RB40 exhibited significant NF-κB activation and accumulation compared to the initial lower-level expression and activation of the pathway.

Our results indicate that the resistant clone created kept raising its tolerance against the drug the longer it was cultured in a Bortezomib-rich medium; however, no multidrug-resistant phenotype emerged as no cross-resistance to anthracyclines or paclitaxel was observed. Key milestones of Bortezomib resistance in the PC-3 RB40 cells were the upregulation of PSMB5 synthesis, the utilization of autophagy as the main proteolytic pathway, and the suppression of oxidative stress levels to levels inferior to those observed in non-resistant cells. This observation was also made by our research using a Bortezomib-resistant DU-145 cell line [11]. The resistant clone was able to downregulate the levels of polyubiquitinated proteins, an observation also made by using hepatocellular carcinoma and prostate cancer cell lines [10,28]. In contrast to Yerlikaya and Okur (2019), in which a Bortezomib-resistant cell line had also been created, we observed the mature from of PSMB5 overexpressed, and no accumulation of its precursor, indicting a different pathway of resistance [10]. Recently, the role of p62 in proteasome inhibitor resistance has gained interest, given the protein’s role as a connection link between ubiquitin-proteasome degradation and ubiquitin-dependent microautophagy [35,64,68]. Resistant cells were found to overexpress p62, confirming data from previous studies that all the autophagy markers assessed indicated a strong indication of the pathway during resistance [11,35,64,116]. Autophagy modulation as an alternative proteolytic mechanism could explain the cells’ ability to control the turnover of several proteins. Normally, various kinases and cell cycle inhibitors are regulated by the UPS since they would otherwise accumulate and lead the cell to apoptotic death. An important role in this process is attributed to Beclin-1, which was also overexpressed in resistant cells. Aside from the regulation of autophagy, it has been found to directly interact with the Bcl-2 anti-apoptotic protein family, thus acting cytoprotectively [44,73,74]. The modulation of signaling molecules with known cytoprotective roles was evident in the resistant clone, mainly focusing on Hsp70 and the stress-related MAPKs p38 and JNK1/SAPK [78,79,81,110]. All three proteins are known to be induced by stress conditions, an observation also evident in naïve cells following treatment with Bortezomib (Figure 11a) [23,80,82]. The accumulation of MEK4 was also observed to be significantly downregulated in the resistant cells, and given the role of MEK4 in phosphorylating both MAPKs [117,118], it could potentially act as a target for cell sensitization to Bortezomib (Figure 13a). The downregulation of key stress markers directed us to oxidative stress levels, which were found to be suppressed in resistant cells. Oxidative stress is believed to rise inside a cell as a result of metabolism modulation, the accumulation of free radicals due to antioxidant defense failure, and specific chemotherapies that can dysregulate these cell functions. Despite Bortezomib being a known oxidative-stress-inducing molecule [21], this was not observed in PC-3 RB40 cells, indicating changes regarding metabolism- and antioxidant-related protein expression. The fortification of the resistant cells’ antioxidant defense systems was verified by assessing SOD1 levels, which were found to be overexpressed in the resistant clones. The reactive oxygen species inside the resistant cells, as measured using H_2_DCFDA, were lower even than those of untreated naïve cells, indicating the new redox equilibrium the resistant cells maintain independently of drug presence or absence. This had also been observed in the DU-145 cell line in a previous publication by our research team [11].

Given the importance of the signal transduction pathways in regulating these parameters, we focused on the main signaling pathways and examined the accumulation and phosphorylation of the main kinases that participate in survival, proliferation, and anti-apoptotic gene expression, namely, the JAK-STAT, Ras-Raf-MEK-ERK, and PI3K-Akt pathways (Figure 13). JAK kinases, mainly JAK1 and JAK2, have been found to participate in the pathogenesis of multiple myeloma, a malignancy susceptible to Bortezomib that can develop resistance as well [119]. JAK silencing has been found to increase cell susceptibility to NK cell-induced cell death in multiple myeloma because both kinases transmit survival signals to the nucleus through the phosphorylation of STATs [120]. Indeed, the resistant cells exhibited an increased accumulation of JAK1, indicating a way to multiply pro-survival signals (Figure 13b). JAK proteins are also known activators of the Ras-Raf-MEK-ERK and PI3K-Akt pathways, and the overexpression of JAK we detected in the resistant clone could contribute to these pathways’ activation [121,122,123,124]. The transcription factors STAT1 and STAT3, downstream of JAK1, were also assessed to monitor the signal transduction route. STAT1, with known pro-apoptotic roles [42,96], was found to be downregulated in the resistant clone both in terms of total STAT1 as well as phosphorylated STAT1. The opposite effect was observed regarding STAT3, which was significantly upregulated in resistant cells. Vangala et al. (2014) documented a correlation between STAT3 phosphorylation and PSMB5 protein levels, indicating STAT3 as a transcription factor that controls proteasome function [102]. STAT3 inhibition downregulated the expression of proteasome subunits, thus increasing the pro-apoptotic effects of Bortezomib. Yuan et al. (2023) also proposed STAT3 inhibition as a way to overcome Bortezomib resistance [39]. Both studies in multiple myeloma reported high STAT3 expression and phosphorylation, which was also observed in the resistant PC-3 RB40 (Figure 13b). This result was also verified by Zafeiropoulou et al. (2024), where the same phenomenon was documented in a Bortezomib-resistant DU-145 prostate cancer cell line [11]. STAT3 has also been reported to be activated by Src in human pancreatic adenocarcinoma cells (independently from JAK activation). This mechanism could shed light on the fact that naïve PC-3 enhanced c-Src accumulation following incubation with a high Bortezomib dose [125]. Hence, this could be an amplification mechanism of the *PSMB5* gene expression to produce more subunits to replace PI-bound proteasomes. Since c-Src was not found to be significantly overexpressed in the resistant cells, this could indicate that the JAK-independent activation could be a significant parameter regarding the (naïve) cell’s primary response to Bortezomib. However, the resistant cells could also be exploiting other ways to achieve STAT3 phosphorylation and overexpression.

Another major role in Bortezomib resistance is played by the ERK1/2 kinases. Being the foremost downstream molecules of the Ras-Raf-MEK-ERK pathway, they capture and transmit to the nucleus signals from several upstream kinases as well as from other pathways due to crosstalk between them [117]. ERK1/2 phosphorylation has already been proposed as a molecular target in myelodysplastic syndromes, where the MEK inhibitors U0126 and PD98059 successfully re-sensitized Bortezomib-resistant cells to the drug [101]. In our experiments, we did not inhibit the signal transduction; however, by assessing the pathway activity, we observed that ERK1/2 was both upregulated and over-phosphorylated in the resistant clones (Figure 13b). Survival mediated by ERK1/2 in Bortezomib resistance has also been shown in other studies, and prostate cancer is one of them [11,126]. ERK1/2 are believed to regulate many cellular processes, from the transcription of survival-related genes to autophagy and even apoptosis. ERK1/2 could also play a crucial role in restoring the cells’ ability to migrate more effectively, given the kinases’ role in cell-adhesion-related signaling. Even though a functional UPS is needed to degrade inhibitors and maintain the equilibrium between pro-survival/pro-migratory and pro-apoptotic signals, over-expressed and over-phosphorylated MAPKs could counterbalance the loss of UPS. Multiplying signals from external stimuli could be a major contributor to the resulting aggressiveness, as this is supported also by the resistant cells’ response during chemotaxis experiments. Resistant cells were able to bypass the chemorepellent effects Bortezomib has on naïve cells, and successfully migrate towards the FBS chambers, highlighting the strong positive signals received from extracellular growth factors. An important downstream molecule of the ERK1/2 kinases is the transcription factor Elk1, a molecule whose role in Bortezomib resistance has only been poorly studied so far.

Few pieces of evidence regarding Elk1 in proteasome inhibitor resistance have been published to date; however, the interaction between ERK1/2-Elk1 and the proteasome has already been proven [127]. A study about Carfilzomib sensitivity in mantle cell lymphoma, a hematological malignancy for which proteasome inhibitors are an approved therapy, investigated the role of Elk1 in proteasome capacity and inhibitor resistance [109]. Elk1 was found to control the proteasome assembling process, regulated by POMP (proteasome maturation protein) expression, which is a molecular chaperone regulating the biosynthesis of proteasome subunits. In that study, the activation of Elk1 through the c-Met/MAPKs led to increased POMP transcription, which in turn increased the assembly of the proteasome and increased both proteasomal activity as well as resistance against Carfilzomib [109]. In our study, the MAPKs were also found to be activated, and the most interesting fact is that Elk1 was found to be significantly overexpressed and phosphorylated in the resistant PC-3 (RB40) cell line (Figure 13b). Elk1 has recently been discovered to play important roles in the progression of many cancer types, including the aggressiveness of pancreatic and prostate cancer [128,129,130]. Modulation of Elk1 activity seems to have anti-metastatic effects, as shown by Lai et al. (2023), where Asiatic acid was administered in PC-3 cells, and by interfering with the protein interaction between Elk1 and MZF-1, migration was negatively affected [131]. Elk1 seems to be an important mediator of resistance by assisting migration towards growth factors and possibly reinforcing the cells’ ability to survive and proliferate, as has also been shown in breast cancer models [132,133,134]. Additionally, Elk1 has been reported to promote survival through autophagy regulation in colorectal cancer cells, a mechanism that could also be present here [135]. The findings about Elk1 were in complete accordance with the observed phenotype of resistant cells; not only were upstream kinases upregulated, but also the transcription factors’ contribution in aggressiveness was visible via the migration assays. Finally, another interesting finding regarding stress-response signaling pathways was the increased accumulation of cJun in the resistant clones. cJun is the last molecule in the JNK signaling cascade and is overexpressed in aggressive cancer types. cJun overexpression has also been linked to constitutively phosphorylated ERK1/2, a fact that was also observed in the PC-3 RB40 cell clone [136]. Given the oncogenic functions of cJun, the suppression of cell cycle regulators like p21, and the proliferative and angiogenic effects of its action, it seems to be a key molecule in resistant cell survival. Both Elk1 and cJun are known to respond to stress conditions, and their stable presence in resistant cells possibly indicates that their suppression mechanisms could have been downregulated (or aborted). Their role in prostate cancer, especially in the case of Elk1, has not been fully uncovered; however, it is known that Elk1 and AR signaling have significant interplay [137]. Given the fact that the cells are immune to external androgen-deprivation therapy, targeting Elk1 as an intracellular component of AR-dependent growth signaling could re-sensitize them by bereaving them of pro-growth signals. Additionally, it is important to mention that Elk1, cJun, and other transcription factors could have even greater translational interest from a biomarker-oriented point of view. Early indices of resistance would assist the clinicians to select the right therapeutic scheme, and non-invasive procedures could further strengthen this notion. Liquid biopsy has recently offered novel biomarkers of high accuracy and significance that can enhance early disease diagnosis and predict prognosis [138]. Monitoring resistance-specific markers and coupling them with liquid biopsy techniques could offer really robust, cost-effective, and, more importantly, less invasive tools against several cancer types. Therefore, the role, expression patterns, and interaction networks of the aforementioned transcription factor should be further studied, and our understanding expanded, not only in Bortezomib resistance, but also in drug-resistant cells in general.

## 5. Conclusions

The model of our study, the PC-3 RB40 cell line, developed resistance to Bortezomib, following exposure to gradually increasing doses of the drug for an extended period. Our experiments demonstrated induction of autophagy, downregulation of oxidative stress levels, and increased signal transduction through the ERK1/2, Akt, and JAK kinases that could be the result of significant crosstalk between the main pathways. Following an investigation of various transcription factors downstream of ERK1/2 and Akt, we discovered that STAT1 was significantly downregulated in the resistant clone and STAT3 was both overexpressed and phosphorylated, thus implying a significant reduction in pro-apoptotic signaling and an induction of pro-survival signals. Additionally, alongside the activation of the Νf-κB pathway, which is already extensively studied in cases of Bortezomib resistance, our study elucidated Elk1 and cJun as potential pharmaceutical targets, given their elevated presence in the resistant clone. Elk1 may be an important mediator of PI resistance, regulating the resistant cell’s cell cycle progression, angiogenesis, and migration, thus assisting survival in hostile environments. With the recent attention the MAPK-Elk1 axis has attracted and the evidence supporting STAT3 and cJun as potential therapeutic targets, we propose the three transcription factors as molecules of a significant role in the emergence of Bortezomib resistance, the targeting of which could truly improve the management of prostate cancer cases by expanding our drug repertoire and disease understanding. Our findings are based on a PI-resistant prostate cancer cell line; however, this does not mean that they are exclusively applicable to prostate cancer. PC-3 cells are known for their stemness, the expression of EMT markers, and the aggressiveness of typical advanced-stage tumor cells, thus allowing extrapolation. Further research is warranted to dissect the molecular pathways that underlie the studied phenomena, and attention should be directed towards the crosstalk between the main survival/proliferation pathways (MAPKs, JAK-STAT, Akt). Targeting of autophagy, ERK1/2 signaling, cJun, and Elk1 could provide insight on drug resistance, not only in PCa but in cases of PI-resistance in general. Even though Bortezomib as a therapy for PCa was assessed in the past and resistance led to a reduced interest, the idea was not abolished since its application as a therapy against mCRPC is still being investigated in clinical trials. Understanding how resistance emerges and develops could encourage further clinical trials and assist clinicians and pharmacologists in compiling novel targeted therapies. Therefore, future studies should expand our research by assessing whether PI therapy could be a part of regimens that would effectively block the aforementioned transcription factors, kinases, or cell functions. It is noteworthy that novel approaches or repurposing of current drugs (by adjusting combinations and doses) could enhance our ability to treat resistant tumors and improve the quality of life of thousands of patients.

## Figures and Tables

**Table 1:** IC_50_ values of PC-3 and PC-3 RB40 cells following treatment with Bortezomib, Carfilzomib, Doxorubicin, or Paclitaxel for 72 h. Each value represents the average of three replicates. The data analysis was performed in Prism 8 using the built-in tools for IC_50_ determination. Comparison between IC_50_ pairs (*n* = 2) was performed with Fisher’s exact tests (F-test) following Shapiro–Wilks normality tests.

Drug	Weeks	IC50, Mean (nM)		IC50 Ratio (Resistant: Naive)	F-Test	
		Naïve PC-3	PC-3 RB40		F (Dfn, Dfd)	*p*-Value
BTZ	4	15.07	21.87	1.45	31.36 (1, 40)	**<0.0001**
	12	16.44	25.47	1.55	21.59 (1, 40)	**<0.0001**
	20	16.16	49.80	3.08	172.2 (1, 40)	**<0.0001**
	28	15.07	51.46	3.41	216.4 (1, 40)	**<0.0001**
	32	15.70	54.64	3.48	704.1 (1, 40)	**<0.0001**
CFZ	4	12.94	14.12	1.09	3.280 (4, 40)	**0.0204**
	32	13.74	26.96	1.96	436.2 (1, 40)	**<0.0001**
DOXO	4	940.2	939.7	0.99	0.00454 (1, 40)	0.9466
	32	941.6	942.6	1.00	0.02971 (1,40)	0.8640
PTX	4	19.00	19.06	1.00	0.03635 (1, 40)	0.8498
	32	19.10	19.18	1.00	0.3044 (1, 40)	0.5842

Notes: BTZ = Bortezomib; CFZ = Carfilzomib; DOXO = Doxorubicin; PTX = Paclitaxel; IC_50_ = half-maximal inhibitory concentration; F-test = Fisher’s exact test; Df = Degrees of freedom.

## Data Availability

The data used for this paper can be found within the paper and its Supporting Files.

## Supplementary Materials

The following supporting information can be downloaded at: https://www.mdpi.com/article/10.3390/cimb47050352/s1.

## Appendix A

### Appendix A.1. Reagents and Consumables

**Table A1:** A complete list of all consumables and reagents.

Item	Manufacturer	Identification Number
*Cell culture reagents*		
RPMI 1640	Biowest (Nuaillé, France)	L0498
FBS	Biowest (Nuaillé, France)	S1810
Penicillin-Streptomycin	Biowest (Nuaillé, France)	L0022
Trypsin	Biowest (Nuaillé, France)	L0932
PBS, sterile filtered	Biowest (Nuaillé, France)	L0616
Bortezomib	Janssen-Cilag International NV (Beerse, Belgium)	5413868123456
Carfilzomib	Amgen Europe B. V. (Breda, The Netherlands)	8715131010515
Doxorubicin	Pfizer Hellas (Athens, Greece)	5415062125588
Paclitaxel	Ratiopharm GmbH (Ulm, Germany)	5053051002986
*Cell culture consumables*		
Cell culture dishes (10 mm, TC treated)	Greiner Bio-One (Kremsmünster, Austria)	664160
Cryovials	Greiner Bio-One (Kremsmünster, Austria)	122263
6-well plates (TC treated)	Greiner Bio-One (Kremsmünster, Austria)	657160
24-well plates (TC-treated)	Greiner Bio-One (Kremsmünster, Austria)	662160
48-well plates (TC-treated)	Greiner Bio-One (Kremsmünster, Austria)	677180
Transwell chambers (PET filter, 8 μm pore diameter)	Greiner Bio-One (Kremsmünster, Austria)	662638
Centrifuge tubes (15 mL)	Greiner Bio-One (Kremsmünster, Austria)	188271
Centrifuge tubes (50 mL)	Greiner Bio-One (Kremsmünster, Austria)	227270
Reaction tubes (1.5 mL)	Greiner Bio-One (Kremsmünster, Austria)	616201
Cell scrapers	Greiner Bio-One (Kremsmünster, Austria)	541070
*Flow cytometry reagents*		
FITC Annexin V	BD Biosciences (Franklin Lakes, NJ, USA)	556420
Propidium Iodide	BD Biosciences (Franklin Lakes, NJ, USA)	556463
LysoTracker™ Red DND-99	Invitrogen™ (Thermo Fisher Scientific) (Waltham, MA, USA)	L7528
H2DCFDA	Invitrogen™ (Thermo Fisher Scientific) (Waltham, MA, USA)	D399
LIVE/DEAD Fixable Far Red Dead Cell Stain Kit	Invitrogen™ (Thermo Fisher Scientific) (Waltham, MA, USA)	L34973
*SDS-PAGE and Western blot reagents*		
SDS	Sigma-Aldrich (Merck) (Darmstadt, Germany)	L3771
Glycine	Sigma-Aldrich (Merck) (Darmstadt, Germany)	G8898
Tris (Trizma Base)	Sigma-Aldrich (Merck) (Darmstadt, Germany)	93352
Acrylamide/Bis-acrylamide (29:1)	Sigma-Aldrich (Merck) (Darmstadt, Germany)	A3574
RIPA Lysis and Extraction Buffer	ThermoScientific™ (Thermo Fisher Scientific) (Waltham, MA, USA)	89901
PVDF Membrane (Immobilon^®^-P, 0.45 μm)	Merck Millipore (Merck) (Burlington, MA, USA)	IPVH00010
Tween^®^ 20	Sigma-Aldrich (Merck) (Darmstadt, Germany)	P1379
SuperSignal™ West Femto Maximum Sensitivity Substrate	ThermoScientific™ (Thermo Fisher Scientific) (Waltham, MA, USA)	34096
Coomassie Brilliant blue R 250	Sigma-Aldrich (Merck) (Darmstadt, Germany)	1125530025

### Appendix A.2. Antibodies

**Table A2:** A complete list of all antibodies used for Western analysis.

Target Protein	Antibody	Company, Catalog Number
N-cadherin	N-Cadherin (D4R1H) XP^®^ Rabbit mAb	CST #13116
E-cadherin	E-Cadherin (24E10) Rabbit mAb	CST #3195
α_ν_β_3_-integrin	Integrin αV/β3/CD51/CD61 (23C6) Mouse mIgG_1_ κ chain	sc-7312
β-catenin	β-Catenin (D10A8) XP^®^ Rabbit mAb	CST #8480
β-actin	Β-Actin (8H10D10) Mouse mAb	CST #3700
Ubiquitin (Ub)	Ubiquitin (P4D1) Mouse mIgG_1_ κ chain	sc-8017
PSMB5	20S Proteasome β5 Antibody (A-10) Mouse mIgG_2a_ κ chain	sc-393931
LC3A/B	LC3A/B (D3U4C) XP^®^ Rabbit mAb	CST #12741
p62/SQSTM1	SQSTM1/p62 Rabbit Ab	CST #5114
Atg5	Atg5 (D5F5U) Rabbit mAb	CST #12994
Beclin-1 (Atg6)	Beclin-1 (D40C5) Rabbit mAb	CST #3495
Hsp70	HSP70 Antibody Rabbit Ab	CST #4872
MEK4	MEK-4 (G-7) Mouse mIgG_1_ κ chain	sc-376838
p-JNK1 ^1^	p-JNK1 (Thr183, Tyr185) Rabbit Ab	# PA5-117403
p-p38 MAPK	Phospho-p38 MAPK (Thr180/Tyr182) (D3F9) XP^®^ Rabbit mAb	CST #4511
SOD1	Superoxide Dismutase 1/SOD1 (24) Mouse mIgG_1_ κ chain	sc-101523
JAK1	Jak1 Rabbit IgG	CST #3332
ERK1/2 MAPKs	p44/42 MAPK (Erk1/2) Rabbit IgG	CST #9102
p-ERK1/2	Phospho-p44/42 (Erk1/2) (Thr202/Tyr204) (D13.14.4E) XP^®^ Rabbit mAb	CST #4370
PI3K p85/p55 ^1^	PI3K p85/p55 Rabbit Recombinant Polyclonal Antibody (6HCLC)	# 710400
p-PI3K p85/p-55	Phospho-PI3 Kinase p85 (Tyr458)/p55 (Tyr199) Rabbit Ab	CST #4228
Akt	Akt Rabbit Ab	CST #9272
p-Akt	Phospho-Akt (Ser473) Rabbit Ab	CST #9271
c-Src	Src Rabbit IgG	CST #2108
p21	p21 Waf1/Cip1 (12D1) Rabbit mAb	CST #2947
p27	p27 Kip1 (D69C12) XP^®^ Rabbit mAb	CST #3686
p53	p53 (1C12) Mouse mAb	CST #2524
NF-κB	NF-κB p65 (D14E12) XP^®^ Rabbit mAb	CST #8242
p-NF-κB	Phospho-NF-κB p65 (Ser536) (93H1) Rabbit mAb	CST #3033
STAT1	Stat1 Rabbit IgG	CST #9172
p-STAT1	Phospho-Stat1 (Tyr701) (58D6) Rabbit mAb	CST #9167
STAT3	Stat3 (D3Z2G) Rabbit mAb	CST#12640
p-STAT3	Phospho-Stat3 (Tyr705) (D3A7) XP^®^ Rabbit mAb	CST#9145
Elk1	Elk-1 Antibody	CST #9182
p-ELK1	Phospho-Elk-1 (Ser383) Antibody	CST #9181
cJun	c-Jun (60A8) Rabbit mAb	CST #9165
Rabbit IgG	Anti-Rabbit IgG, HRP-linked	CST #7074
Mouse IgG	Anti-Mouse IgG HRP-linked	CST #7076

^1^ These antibodies were purchased from Thermo Fisher Scientific (Invitrogen™, Waltham, MA, USA).

## Appendix C

### Appendix C.1. Cell Cycle Analysis

**Table A3:** Comparisons between different samples regarding cell-cycle phase distribution using the chi-square test. Statistical significance is set at 0.05. Statistically significant findings are highlighted using a bold *p*-value.

Samples	Chi-Square (Df)	*p*-Value
Untreated PC-3 vs. PC-3 treated with 40 nM BTZ	18.65 (2)	**<0.0001**
Untreated PC-3 RB40 vs. PC-3 RB40 treated with 40 nM BTZ	0.6114 (2)	0.7366
Untreated PC-3 vs. untreated PC-3 RB40 BTZ	0.1898 (2)	0.9095
PC-3 treated with 40 nM BTZ vs. PC-3 RB40 treated with 40 nM BTZ	11.52 (2)	**0.0031**
Untreated PC-3 vs. PC-3 RB40 treated with 40 nM BTZ	1.266 (2)	0.5310

Notes: BTZ = Bortezomib; Df = Degrees of Freedom.

### Appendix C.2. Apoptosis Analysis

**Table A4:** Comparisons between different samples regarding viability status using the chi-square test. Statistical significance is set at 0.05. Statistically significant findings are highlighted using a bold *p*-value.

Samples	Chi-Square (Df)	*p*-Value
Untreated PC-3 vs. PC-3 treated with 40 nM BTZ	118.4 (3)	**<0.0001**
Untreated PC-3 RB40 vs. PC-3 RB40 treated with 40 nM BTZ	2.950 (3)	0.3995
Untreated PC-3 vs. untreated PC-3 RB40 BTZ	0.1853 (3)	0.9799
PC-3 treated with 40 nM vs. PC-3 RB40 treated with 40 nM BTA	95.33 (3)	**<0.0001**
PC-3 RB40 treated with 40 nM BTZ vs. PC-3 RB40 treated with 80 nM BTZ	9.966 (3)	**0.0189**
Untreated PC-3 vs. PC-3 RB40 treated with 40 nM BTZ	4.185 (3)	0.2422

Notes: BTZ = Bortezomib; Df = Degrees of Freedom.

## Appendix D

### Appendix D.1. Lysotracker RED

**Table A5:** Comparisons between different samples regarding Lysotracker staining MFI using one-way ANOVA and Tukey’s multiple comparisons. Statistical significance is set at 0.05. Statistically significant findings are highlighted using a bold *p*-value.

ANOVA Table	SS	MS	Df	F (Dfn, Dfd)	*p*-Value
Samples (columns)	12,439	4146	3	F (3, 8) = 2087	**<0.0001**
Replicate experiments (rows)	15.89	1.987	8		
Total	12,455	4146	11		
**Samples**			**Df**	**Adjusted *p*-value**	
Untreated PC-3 vs. PC-3 treated with 40 nM BTZ			8	**<0.0001**	
Untreated PC-3 RB40 vs. PC-3 RB40 treated with 40 nM BTZ			8	0.0802	
Untreated PC-3 vs. Untreated PC-3 RB40			8	**0.0001**	
PC-3 treated with 40 nM vs. untreated PC-3 RB40			8	**<0.0001**	
PC-3 treated with 40 nM BTZ vs. PC-3 RB40 treated with 40 nM BTZ			8	**<0.0001**	
Untreated PC-3 vs. PC-3 RB40 treated with 40 nM BTZ			8	**<0.0001**	

Notes: BTZ = Bortezomib; Df = Degrees of Freedom; SS = Sum of Squares; MS = Mean Squares.

### Appendix D.2. LC3 Analysis

LC3 is an autophagy marker which can provide significant information about autophagy activation, flux, and phase. Both total LC3A/B as well as the ratio between LC3A/B I and LC3A/B II can be extracted from Western blot data and were herein calculated using information from quantified blots.

**Table A6:** LC3A/B percentages. Quantification of the scanned blots was performed using the plug-in “Gel Blots” in ImageJ after conversion of the scanned blots to grayscale images. The data were retrieved from triplicate experiments and the normalization was performed using β-actin. Each value represents the average percentage of the target protein compared to the respective control. Each experiment was replicated three times, and the annotated error is the standard error of the mean (SEM).

	Naïve PC-3			PC-3 RB40			
**Bortezomib (nM)**	**0**	**20**	**40**	**0**	**20**	**40**	**80**
*Normalized Signal (β-actin) Compared to the Control*							
LC3A/B	100 ± 0.49	105.88 ± 0.05	244.54 ± 2.04	104.16 ± 0.20	95.32 ± 0.25	96.68 ± 0.20	107.40 ± 0.07
LC3A/B I	100 ± 1.15	78.06 ± 0.01	248.06 ± 3.98	136.79 ± 0.08	92.93 ± 0.60	110.45 ± 0.33	127.34 ± 0.05
LC3A/B II	100 ± 0.17	131.9 ± 0.12	241.24 ± 0.24	73.64 ± 0.29	97.56 ± 0.14	83.8 ± 0.07	88.75 ± 0.08
*Normalized Signal (β-actin)*							
LC3A/B I	1.87 ± 0.00	1.46 ± 0.00	4.63 ± 0.07	2.55 ± 0.00	1.73 ± 0.01	2.06 ± 0.01	2.38 ± 0.00
LC3A/B II	2 ± 0.00	2.63 ± 0.00	4.81 ± 0.00	1.47 ± 0.00	1.95 ± 0.00	1.67 ± 0.00	1.77 ± 0.00
Ratio	1.07	1.80	1.04	0.58	1.13	0.81	0.74

## Appendix E

### Appendix E.1. Oxidative Stress Analysis (24 H Assay)

**Table A7:** Comparisons between different samples regarding H_2_DCFDA staining MFI using one-way ANOVA and Tukey’s multiple comparisons. Statistical significance is set at 0.05. Statistically significant findings are highlighted using a bold *p*-value.

ANOVA Table	SS	MS	Df	F (Dfn, Dfd)	*p*-Value
Samples (columns)	504,835	168,278	3	F (3, 8) = 2785	**<0.0001**
Replicate experiments (rows)	483.3	60.42	8		
Total	505,319				
**Samples**			**Df**	**Adjusted *p*-value**	
Untreated PC-3 vs. PC-3 treated with 40 nM			8	**<0.0001**	
Untreated PC-3 RB40 vs. PC-3 RB40 treated with 40 nM			8	**<0.0001**	
Untreated PC-3 vs. Untreated PC-3 RB40			8	**<0.0001**	
PC-3 treated with 40 nM vs. untreated PC-3 RB40			8	**<0.0001**	
PC-3 treated with 40 nM vs. PC-3 RB40 treated with 40 nM			8	**<0.0001**	
Untreated PC-3 vs. PC-3 RB40 treated with 40 nM			8	**<0.0001**	

Notes: BTZ = Bortezomib; Df = Degrees of Freedom; SS = Sum of Squares; MS = Mean Squares.

### Appendix E.2. Extensive Reactive Oxygen Species Assays

**Table A8:** ROS levels percentages. The data were retrieved from triplicate flow cytometry experiments. Each value represents the average percentage of the target protein compared to the respective control. Each experiment was replicated three times, and the annotated error is the standard error of the mean (SEM).

	Incubation Time (h)	12	24	36
Cell line	Bortezomib (nM)	% Compared to Untreated PC-3 Cells		
Naïve PC-3	0	100 ± 1.41	100.75 ± 1.02	101.03 ± 1.21
	40	107.78 ± 2.35	130.74 ± 2.51	198.97 ± 1.37
PC-3 RB40	0	82.01 ± 0.69	90.44 ± 0.83	81.07 ± 1.16
	40	78.63 ± 1.19	79.01 ± 0.36	79.19 ± 0.59
	80	80.88 ± 1.97	92.31 ± 1.52	103.09 ± 2.09

## Appendix F

Cell migration was assessed using wound healing assays and Boyden chambers. Given the visual aspect of these experiments, representative images are presented herein while in the article corpus, quantitative data is the preferred form.

### Appendix F.1. Wound Healing

#### Appendix F.1.1. Wound Healing Images

For the scratch tests/wound healing assays, cells were cultured in 6-well plates until confluency, and then scratches were made using sterile P200 pipette tips. Both clones were treated with Bortezomib (PC-3 RB40: 20, 40, and 80 nM of Bortezomib; naïve PC-3: 20 and 40 nM of Bortezomib, the high dose of 80 nM was cytotoxic to naïve cells and was not used), and photographs were taken at the key time points of 0, 24, 48, and 72 h using a camera mounted on an inverted microscope at 100× magnification. The photographs were converted into grayscale images and analyzed using an ImageJ plug-in to determine the healing rates. Data from the triplicate experiment were used and subsequently plotted in Prism 8 to create the figures appearing in the article corpus.

#### Appendix F.1.2. Wound Healing Statistics

Data regarding the healing rates were collected in Prism 8 and analyzed using two-way ANOVA. Comparisons were performed in pairs shown in Table A4.

**Table A9:** Two-way ANOVA analysis to detect differences in wound healing rates. Statistical significance is set at 0.05. Statistically significant findings are highlighted using a bold *p*-value.

Samples	F (Dfn, Dfd)	*p*-Value
Untreated PC-3 vs. PC-3 treated with 20 nM BTZ	2.455 (1, 6)	0.1682
Untreated PC-3 vs. PC-3 treated with 40 nM BTZ	141.9 (1, 6)	**<0.0001**
Untreated PC-3 RB40 vs. PC-3 RB40 treated with 40 nM BTZ	4.748 (1, 6)	0.0948
Untreated PC-3 RB40 vs. PC-3 RB40 treated with 80 nM BTZ	92.21 (1, 6)	**0.0007**
Untreated PC-3 vs. untreated PC-3 RB40 BTZ	0.2185 (1, 6)	0.6598
Untreated PC-3 vs. PC-3 RB40 treated with 40 nM BTZ	2.604 (1, 6)	0.1675
PC-3 Treated with 20 nM BTZ vs. PC-3 RB40 treated with 20 nM BTZ	0.06341 (1, 6)	0.8112
PC-3 treated with 40 nM BTZ vs. PC-3 RB40 treated with 40 nM BTZ	103.7 (1, 6)	**0.0002**
PC-3 treated with 40 nM BTZ vs. PC-3 RB40 treated with 80 nM BTZ	0.4375 (1, 6)	0.5376

Notes: BTZ = Bortezomib; Df = Degrees of Freedom.
