# Supplementary material for: The Transcription Axes ERK-Elk1, JNK-cJun, and JAK-STAT Promote Autophagy Activation and Proteasome Inhibitor Resistance in Prostate Cancer Cells
Source: Curr Issues Mol Biol. 2025 May 12;47(5):352. doi: 10.3390/cimb47050352 (PMC12110616; doi:10.3390/cimb47050352)
Supplement: Supplementary file 1 [file cimb-47-00352-s001.zip › cimb-3614166-supplementary.pdf]

current issues in molecular biology

MDPI

Supplementary Information

The Transcription Axes ERK-Elk1, JNK-cJun, and JAK-STAT Promote Autophagy Activation and Proteasome Inhibitor Resistance in Prostate Cancer Cells

Georgios Kalampounias [1], Kalliopi Zafeiropoulou [1,2,†], Theodosia Androutsopoulou [1,†], Spyridon Alexis [2], Argiris Symeonidis [2] and Panagiotis Katsoris [1,*]

[1] Laboratory of Cell Biology, Division of Genetics, Cell and Developmental Biology, Department of Biology, School of Natural Sciences, University of Patras, 26504 Patras, Greece; gkalampounias@ac.upatras.gr (G.K.); kzafeirop@upatras.gr (K.Z.); tandroutsopoulou@ac.upatras.gr (T.A.)
[2] Hematology Division, Faculty of Medicine, School of Health Sciences, University of Patras, 26504 Patras, Greece; spirosal1@hotmail.com (S.A.); argiris.symeonidis@yahoo.gr (A.S.)
* Correspondence: katsopan@upatras.gr
† These authors contributed equally to this work.

Supplement

S.1. Viability/Proliferation Curves and IC_{50} Determination

Supplement S.1.1. Viability/Proliferation Curves

Assessment of the effects of Bortezomib, Carfilzomib, Doxorubicin and Docetaxel on the Proliferation and viability of naïve PC-3 and PC-3 RB40 cells was performed by estimating the cell number following 3 days of incubation with the drugs. Equal numbers of cells were cultured for 72 h in 24-well microplates with various concentrations of the drugs were used to determine the concentration at which a 50% inhibition was evident (IC_{50}). The resistant cells were constantly maintained in medium containing Bortezomib, and they assessed at various time points (4, 12, 20, 28, and 32 weeks) to estimate the resistance level. Cross-resistance to Carfilzomib was also tested, as well as resistance to different chemotherapeutics like Doxorubicin and Paclitaxel. The number of live cells was determined by staining them with crystal violet and spectrophotometrically determining the amount of bound stain. Standard curves were subsequently used to correlate O.D. to cell number and the results are presented in scatter plots.

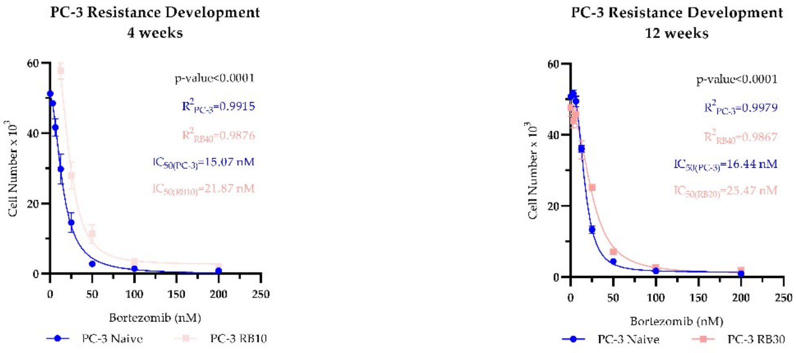

(a) (b)

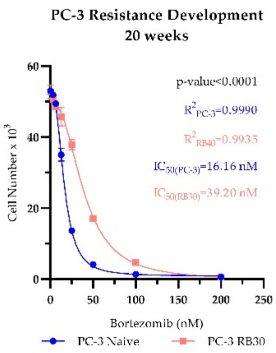

(**c**)

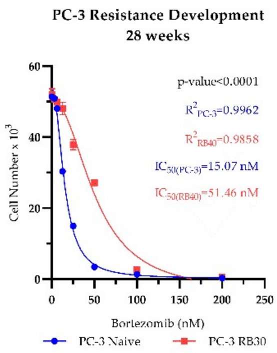

(**d**)

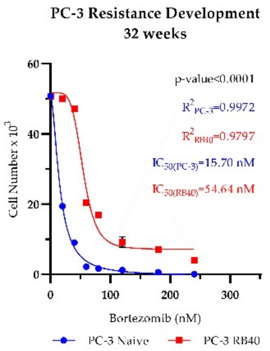

(**e**)

Figure S1. Viability/Proliferation assays of cell treated for 72 h with Bortezomib (0 – 240 nM) following: (**a**) 4 weeks of resistance acquirement; (**b**) 12 weeks of resistance acquirement; (**c**) 20 weeks of resistance acquirement; (**d**) 28 weeks of resistance acquirement; (**e**) 32 weeks of resistance acquirement. Each dot represents the average of three experimental values, and the error bars represent the standard error of the mean (SEM). The fitting lines were graphed in Prism 8 using the built-in model for IC_{50} determination. Blue lines represent naïve PC-3 cells and red lines correspond to PC-3 RB40 cells, both treated with Bortezomib. Each plot represents one experiment.

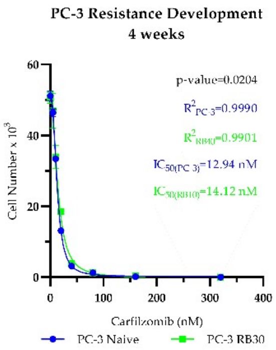

(**a**)

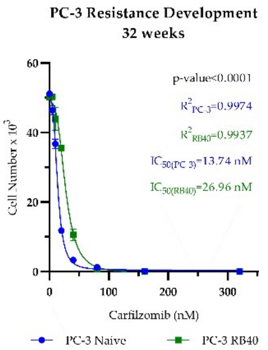

(**b**)

Figure S2. Viability/Proliferation assays of cell treated for 72 h with Carfilzomib (0 – 325 nM) following: (**a**) 4 weeks of resistance acquirement; (**b**) 32 weeks of resistance acquirement. Each dot represents the average of three experimental values, and the error bars represent the standard error of the mean (SEM). The fitting lines were graphed in Prism 8 using the built-in model for IC_{50} determination. Blue lines represent naïve PC-3 cells and green lines correspond to PC-3 RB40 cells, both treated with Carfilzomib. Each plot represents one experiment.

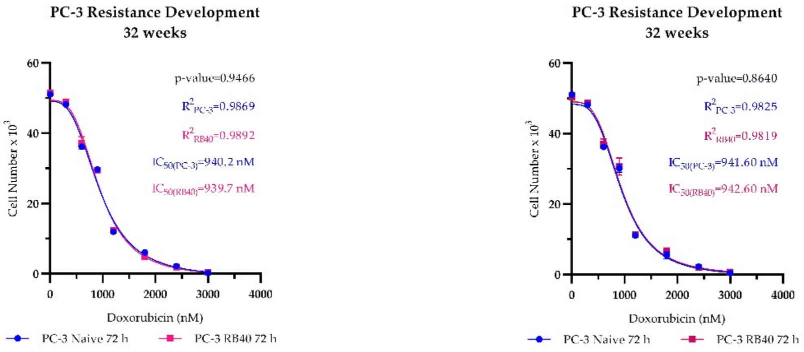

(**a**) (**b**)

Figure S3. Viability/Proliferation assays of cell treated for 72 h with Doxorubicin (0.3 – 3 nM) following: (**a**) 4 weeks of resistance acquirement; (**b**) 32 weeks of resistance acquirement. Each dot represents the average of three experimental values, and the error bars represent the standard error of the mean (SEM). The fitting lines were graphed in Prism 8 using the built-in model for IC_{50} determination. Blue lines represent naïve PC-3 cells and magenta lines correspond to PC-3 RB40 cells, both treated with Doxorubicin. Each plot represents one experiment.

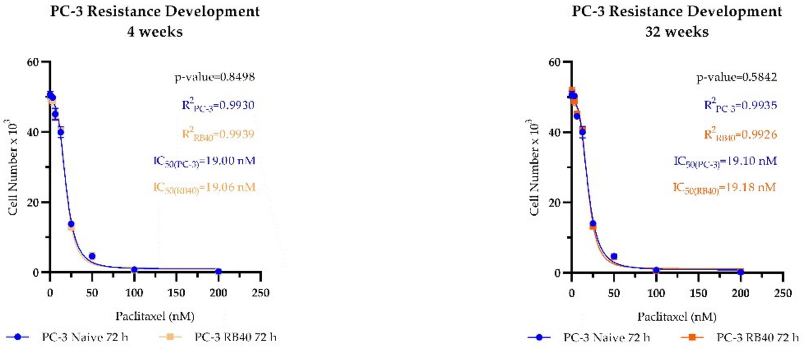

(**a**) (**b**)

Figure S4. Viability/Proliferation assays of cell treated for 72 h with Paclitaxel (0 – 240 nM) following: (**a**) 4 weeks of resistance acquirement; (**b**) 32 weeks of resistance acquirement. Each dot represents the average of three experimental values, and the error bars represent the standard error of the mean (SEM). The fitting lines were graphed in Prism 8 using the built-in model for IC_{50} determination. Blue lines represent naïve PC-3 cells and orange line correspond to cells, both treated with Paclitaxel. Each plot represents one experiment.

Supplement S.1.2. Viability Data Normality

Table S1. Shapiro-Wilks test to determine normality of viability data.

		Naïve PC-3			PC-3 RB40		
Week	*Drug*	*IC_{50}*	*R^2*	*Shapiro-Wilks p-value*	*IC_{50}*	*R^2*	*Shapiro-Wilks p-value*
	BTZ	15.07	0.9915	0.8056	21.87	0.9876	0.5992
4	*CFZ*	12.94	0.9990	0.8073	14.12	0.9901	0.0559
	DOXO	940.2	0.9869	0.1607	939.7	0.9892	0.4566
	PTX	19.00	0.9939	0.1914	19.06	0.9930	0.5600
12	*BTZ*	16.44	0.9979	0.6607	25.47	0.9867	0.6615

20	*BTZ*	16.16	0.9990	0.0714	49.80	0.9935	0.0592
28	*BTZ*	15.07	0.9962	0.6972	51.46	0.9858	0.2629
32	*BTZ*	15.70	0.9972	0.1820	54.64	0.9797	0.1151
	CFZ	13.74	0.9974	0.5290	26.96	0.9937	0.0603
	DOXO	941.6	0.9825	0.2230	942.6	0.9819	0.1376
	PTX	19.10	0.9935	0.1616	19.18	0.9926	0.1507

Notes: IC_{50}=half-maximum inhibitory concentration; R^2=Coefficient of Determination; BTZ= Bortezomib; CFZ=Carfilzomib; DOXO=doxorubicin; PTX=paclitaxel.

Supplement S.1.1. Goodness of Fit for the IC_{50} Model and Comparisons

Table S2. IC_{50} determination model and comparison of fits.

		**Naïve PC-3 IC_{50} (nM)**			**PC-3 RB40 IC_{50} (nM)**			**Exact F-test**	
Week	***Drug***	***IC_{50}***	***Df***	***R^2***	***IC_{50}***	***Df***	***R^2***	***F (Dfn, Dfd)***	***p-value***
4	*BTZ*	15.07	20	0.9915	21.87	20	0.9876	31.36 (1, 40)	**<0.0001**
	CFZ	12.94	20	0.9990	14.12	20	0.9901	3.280 (4, 40)	**0.0204**
	DOXO	940.2	20	0.9869	939.7	20	0.9892	0.004544 (1, 40)	0.9466
	PTX	19.00	20	0.9939	19.06	20	0.9930	0.03635 (1, 40)	0.8498
12	*BTZ*	16.44	20	0.9979	25.47	20	0.9867	21.59 (4, 40)	**<0.0001**
20	*BTZ*	16.16	20	0.9990	49.80	20	0.9935	172.2 (4, 40)	**<0.0001**
28	*BTZ*	15.07	20	0.9962	51.46	20	0.9858	216.4 (1, 40)	**<0.0001**
32	*BTZ*	15.70	20	0.9972	54.64	20	0.9797	704.1 (1, 40)	**<0.0001**
	CFZ	13.74	20	0.9974	26.96	20	0.9937	436.2 (1, 40)	**<0.0001**
	DOXO	941.6	20	0.9825	942.6	20	0.9819	0.02971 (1, 40)	0.8640
	PTX	19.10	20	0.9935	19.18	20	0.9926	0.3044 (1, 40)	0.5842

Notes: IC_{50}=half-maximum inhibitory concentration; Df=degrees of Freedom; R^2=Coefficient of Determination; BTZ= Bortezomib; CFZ=Carfilzomib; DOXO=doxorubicin; PTX=paclitaxel.

S.2. Western Blot Quantification using ImageJ

Following incubation with specific primary and secondary antibodies, chemiluminescence was developed on autoradiography films, which were subsequently scanned. After conversion to grayscale images, quantification of the scanned blots was followed using the plug-in “Gels” in ImageJ, and the band intensities and bit depth were calculated. Besides visualizing the most evident qualitative changes in protein accumulation, Western blots can provide semi-quantitative data, which were herein used to further support our observations. To compare the protein accumulation between all lanes, some signal saturation may be present in certain lanes. This was inevitable since PC-3 RB40 cells had overexpressed proteins barely detectable in the naïve clone or vice versa. Quantification of such lanes (and, in general, quantification) is not crucial for this study; therefore, we proceeded to present such data as it can be useful to indicate differential dose-dependent responses observed in the two clones. For this reason, presenting the blot data in their unprocessed and unquantified form in the article corpus was preferred, with the sole addition of heatmaps to enhance visualization and readability. Quantitative information is presented in the Appendix and is also cited in the main text. To obtain such data, blots from triplicate experiments were used, and β-actin was used to normalize the signal. Comparisons were performed between lanes of the same film only, and whenever lanes have been cropped out, it is annotated by a dashed line inside the figure. To analyze the results,

multiple comparisons of one-way ANOVA in the Prism 8 software were performed, and the data was plotted as bar charts.

S.2.1. Ubiquitin-Proteasome System (UPS) and Autophagy Proteins

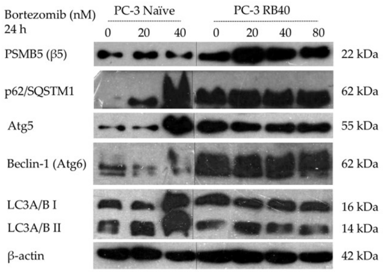

(**a**)

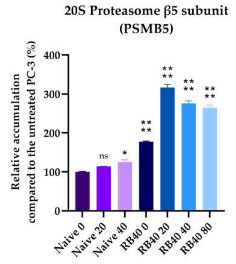

(**b**)

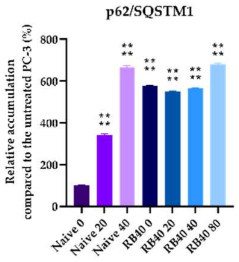

(**c**)

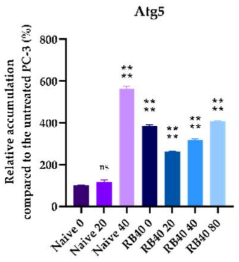

(**d**)

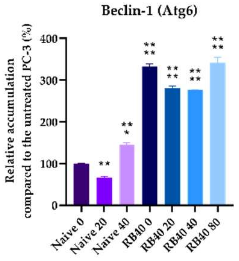

(**e**)

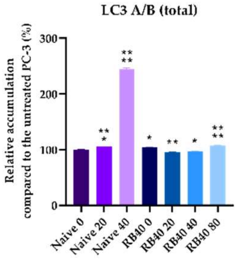

(**f**)

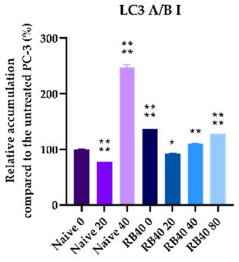

(**g**)

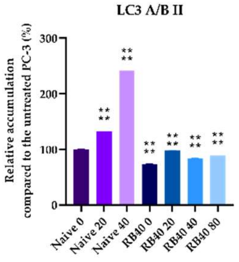

(**h**)

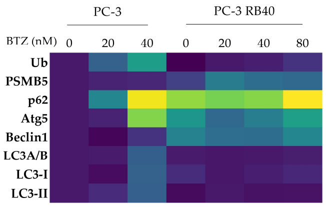

(**i**)

Figure S5. Western analysis and quantification of main UPS and autophagy proteins. (**a**) Representative western blots of PSMB5, p62, Atg5, Beclin-1 (Atg6), LC3A/B I and LC3A/B II. (**b–h**) Quantitative data were obtained from scanned films (of triplicate experiments). Each bar represents the average relative accumulation of the target protein compared to the untreated naïve PC-3 cells (first blot lane). (* corresponds to a p-value = 0.01; ** corresponds to a p-value = 0.001; *** corresponds to a p-value = 0.0001; and **** corresponds to a p-value < 0.0001). The error bars represent the standard error of the mean (SEM). (**i**) Data from the bar charts were used to create heatmaps for enhanced visualization.

S.2.2. Stress Markers and Cell Cycle Regulators

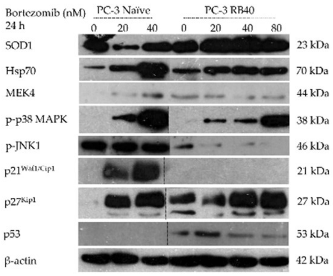

(**a**)

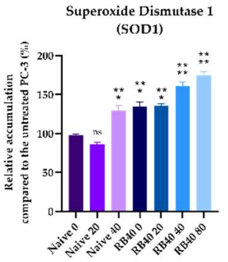

(**b**)

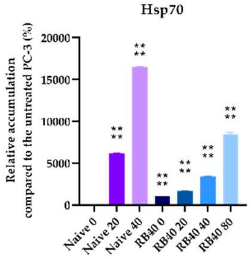

(**c**)

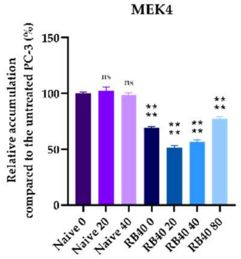

(**d**)

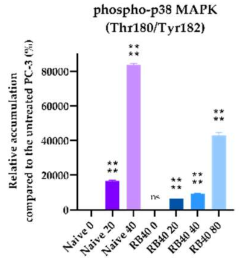

(**e**)

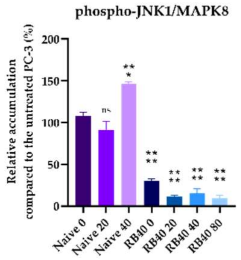

(**f**)

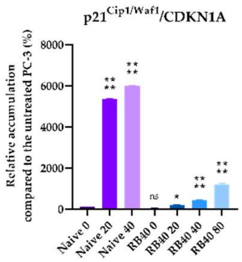

(**g**)

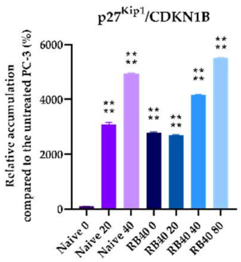

(**h**)

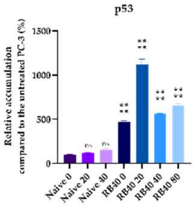

(i)

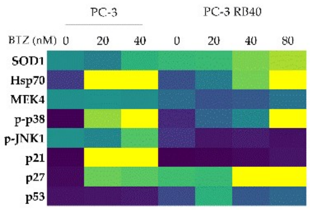

(j)

Figure S6. Western analysis and quantification of stress markers and cell cycle regulators. (**a**) Representative western blots of SOD1, Hsp70, MEK4, p-p38 (p-MAPK11), p-JNK1 (p-MAPK8), $p21^{waf/cip1}$/CDKN1A, $p27^{kip1}$/CDKN1B, and p53. (**b–i**) Quantitative data was obtained from scanned films (of triplicate experiments) Each bar represents the average relative accumulation of the target protein compared to the untreated naïve PC-3 cells (first blot lane). (* corresponds to a p-value = 0.01; *** corresponds to a p-value = 0.0001; and **** corresponds to a p-value < 0.0001). The error bars represent the standard error of the mean (SEM). (**j**) Data from the bar charts were used to create heatmaps for enhanced visualization.

S.2.3. EMT Markers

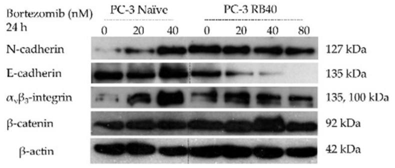

(a)

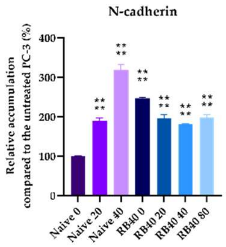

(b)

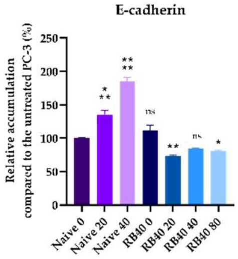

(c)

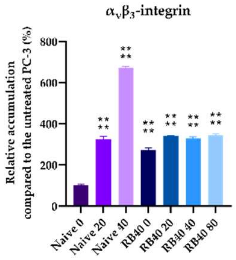

(d)

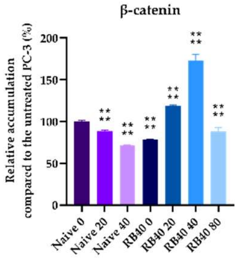

(e)

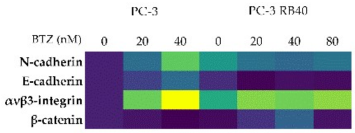

(f)

Figure S7. Western analysis and quantification of main UPS and autophagy proteins. (**a**) Representative western blots of N-cadherin, E-cadherin, and $\alpha_v\beta_3$-integrin. (**b–e**) Quantitative data were obtained from scanned films (of triplicate experiments). Each bar represents the average relative

accumulation of the target protein compared to the untreated naïve PC-3 cells (first blot lane). (* corresponds to a p-value = 0.01; ** corresponds to a p-value = 0.001; *** corresponds to a p-value = 0.0001; and **** corresponds to a p-value < 0.0001). The error bars represent the standard error of the mean (SEM). (**f**) Data from the bar charts were used to create heatmaps for enhanced visualization.

S.2.4. Signaling Through JAK-STAT, NF-κβ, Src, and cJun

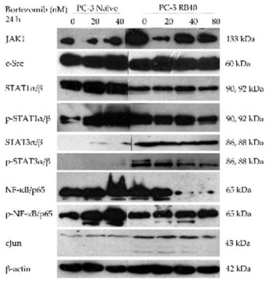

(**a**)

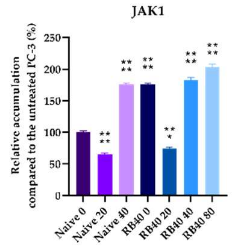

(**b**)

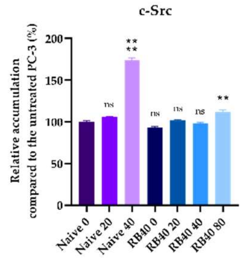

(**c**)

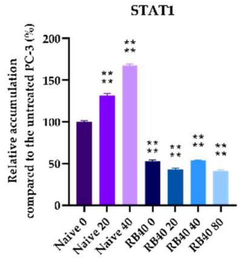

(**d**)

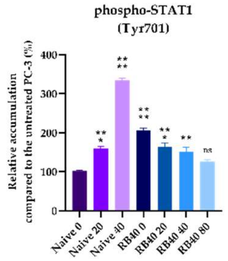

(**e**)

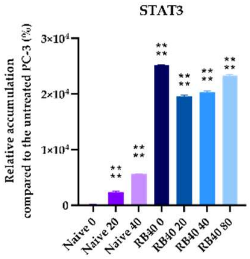

(**f**)

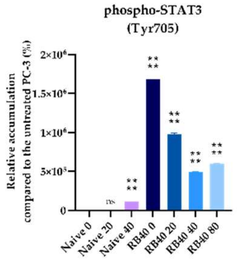

(**g**)

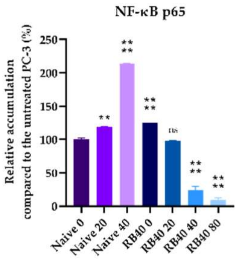

(**h**)

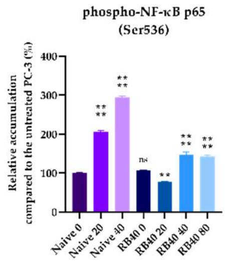

(**i**)

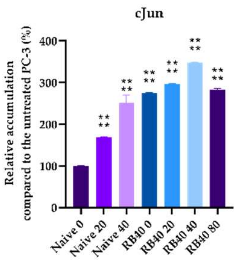

(**j**)

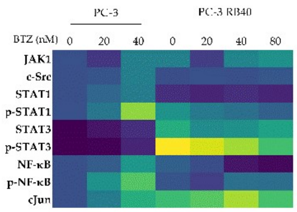

(**k**)

Figure S8. Western analysis and quantification of JAK-STAT, NF-κβ, and cJun pathways' main proteins. (**a**) Representative western blots of JAK1, STAT1, p-STAT1, STAT3, p-STAT3, c-Src, NF-κβ, p-NF-κβ, and cJun. (**b–j**) Quantitative data was obtained from scanned films (of triplicate experiments). Each bar represents the average relative accumulation of the target protein compared to the untreated naïve PC-3 cells (first blot lane). (** corresponds to a p-value =0.001; *** corresponds to a p-value =0.0001; and **** corresponds to a p-value <0.0001). The error bars represent the standard error of the mean (SEM). (**k**) Data from the bar charts were used to create heatmaps for enhanced visualization.

S.2.4. Signaling Through PI3K-Akt and ERK1/2-Elk1

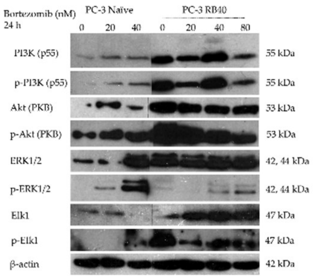

(**a**)

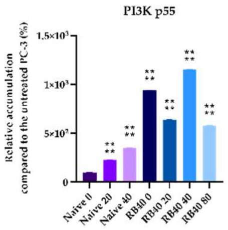

(**b**)

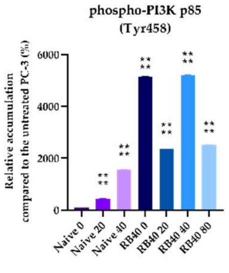

(**c**)

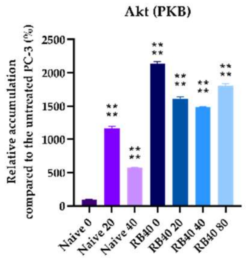

(**d**)

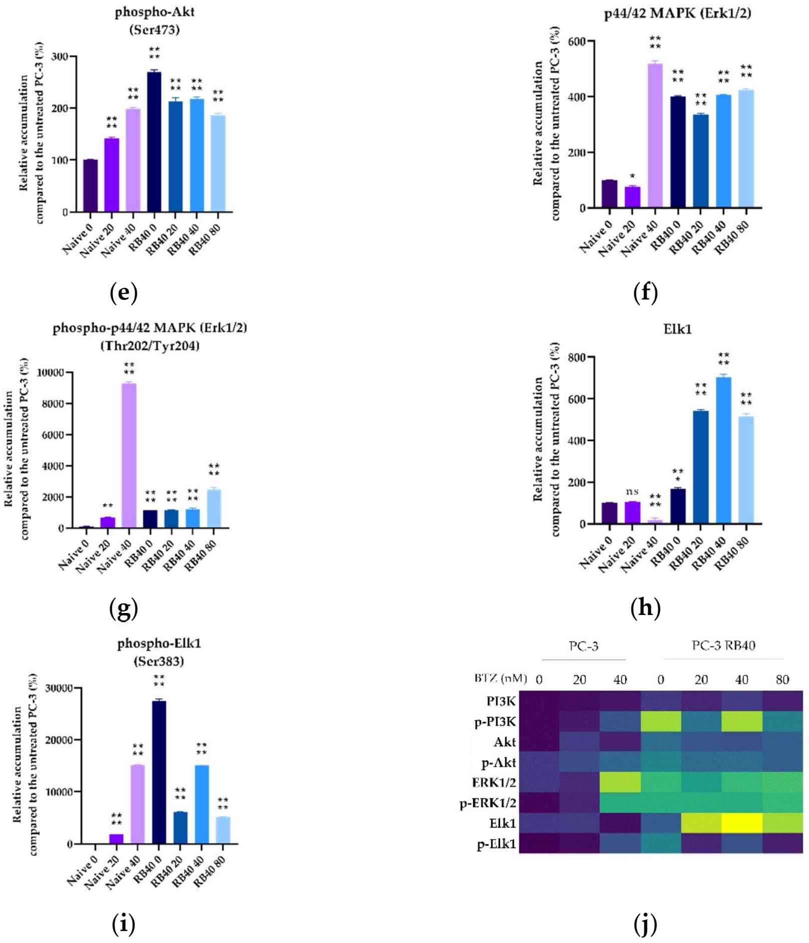

Figure S9. Western analysis and quantification of PI3K-Akt and ERK1/2-Elk1 pathways' main proteins. (**a**) Representative western blots of: PI3K (p85/55), p-PI3K (p-p85/55), Akt (PKB), p-Akt (p-PKB), ERK1/2 (MAPK3/1), p-ERK1/2 (p-MAPK3/1), Elk1, p-Elk1. (**b**–**i**) Quantitative data was obtained from scanned films (of triplicate experiments). Each bar represents the average relative accumulation of the target protein compared to the untreated naïve PC-3 cells (first blot lane). (* corresponds to a p-value = 0.01; ** corresponds to a p-value = 0.001; *** corresponds to a p-value = 0.0001; and **** corresponds to a p-value < 0.0001). The error bars represent the standard error of the mean (SEM). (**j**) Data from the bar charts were used to create heatmaps for enhanced visualization.

S.3. Time-Course and Dose-Response Reactive Oxygen Species Assays

Intracellular oxidative stress was assessed using H_2DCFDA, which stains hydroxyl radicals. Confluent petri dishes were treated with the designated Bortezomib concentrations for 24 h prior analysis and cells were collected by trypsinization. Cells were stained with H_2DCFDA and were subsequently analyzed on a FACSCalibur flow cytometer. To perform time-course experiments, confluent sets of petri dishes were divided and subculture into three identical sets of dishes, until confluency. Bortezomib was added: to the first set of samples 36 h prior analysis, to the second set of samples 24 h prior analysis, and to the last set of samples 12 h prior analysis. Samples of the same set were analyzed contemporaneously and the results of three separate sample sets were used for the analysis. Two-

way ANOVAs were used to analyze ROS level augmentation, to estimate both the effects of time and dosage. The MFI is presented in Figure S10 and the statistical comparisons in Table S3.

Table S3. Two-way ANOVA analysis to detect differences in intracellular oxidative stress levels. Statistical significance is set at 0.05. Statistically significant findings are highlighted using a bold p-value.

Samples	F (Dfn, Dfd)	p-value
Untreated PC-3 vs PC-3 treated with 40 nM	F (2, 8) = 4260	**<0.0001**
Untreated PC-3 vs untreated PC-3 RB40	F (2, 8) = 139.0	**<0.0001**
Untreated PC-3 RB40 vs PC-3 RB40 treated with 40 nM	F (2, 8) = 151.5	**<0.0001**
Untreated PC-3 RB40 vs PC-3 RB40 treated with 80 nM	F (2, 8) = 1544	**<0.0001**
Untreated PC-3 vs PC-3 RB40 treated with 40 nM	F (2, 8) = 1.529	0.2739
Untreated PC-3 vs PC-3 RB40 treated with 80 nM	F (2, 8) = 1512	**<0.0001**
PC-3 treated with 40 nM vs PC-3 RB40 treated with 40 nM	F (2, 8) = 4261	**<0.0001**
PC-3 treated with 40 nM vs PC-3 RB40 treated with 80 nM	F (2, 8) = 2366	**<0.0001**

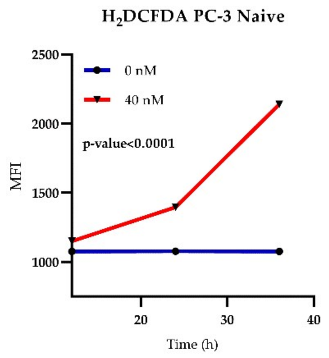

(**a**)

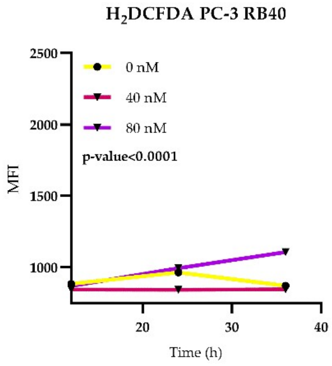

(**b**)

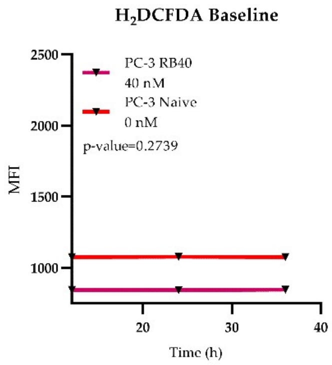

(**c**)

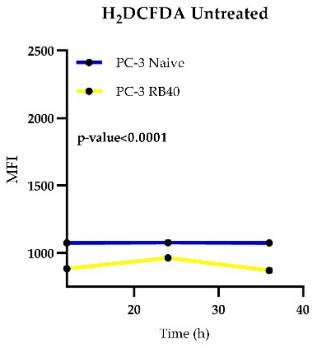

(**d**)

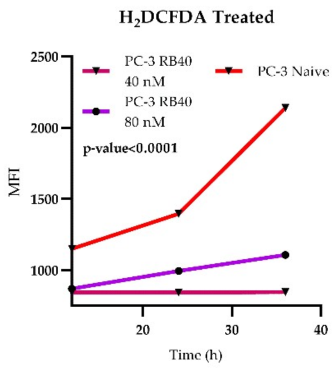

(**e**)

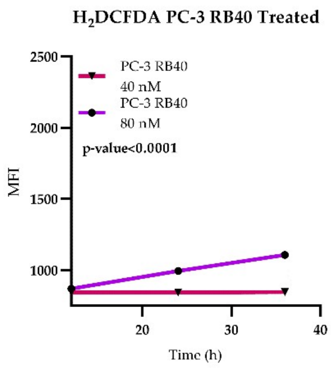

(**f**)

Figure S10. ROS time-course assay of: (**a**) Naïve PC-3 cells; (**b**) PC-3 RB40 cells; (**c**) Cells at their baseline conditions; (**d**) Untreated naïve and PC-3 RB40 cells; and (**e**) Cells of both cell clones treated with Bortezomib; (**f**) PC-3 RB40 cells treated with 80 nM of Bortezomib. Each graph represents three independent experiments. The error bars correspond to the standard deviation (SD) from the three experiments. The annotated p-values are the result of two-way ANOVAs. Statistical significance was set at 0.05.
