# Supplementary material for: The Transcription Axes ERK-Elk1, JNK-cJun, and JAK-STAT Promote Autophagy Activation and Proteasome Inhibitor Resistance in Prostate Cancer Cells
Source: Curr Issues Mol Biol. 2025 May 12;47(5):352. doi: 10.3390/cimb47050352 (PMC12110616; doi:10.3390/cimb47050352)
Supplement: Supplementary file 1 [file cimb-47-00352-s001.zip › Supporting_Raw_Images.pdf]

Western Blots

Supporting Data

Kalampounias G. et al, 2025

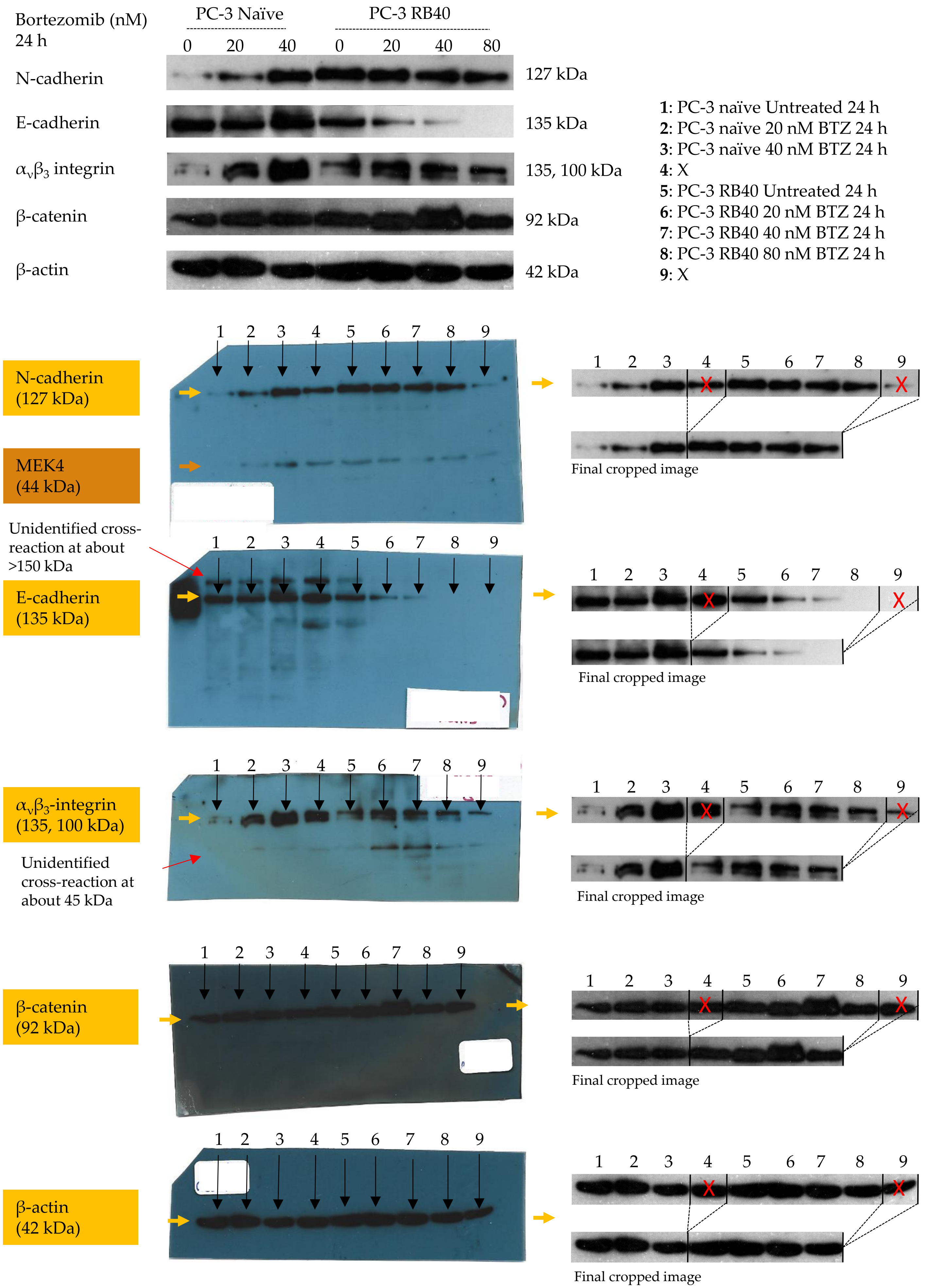

Bortezomib (nM)
24 h
PC-3 Naïve
PC-3 RB40
0 20 40 0 20 40 80
N-cadherin
127 kDa
E-cadherin
135 kDa
$\alpha_v\beta_3$ integrin
135, 100 kDa
β-catenin
92 kDa
β-actin
42 kDa
1: PC-3 naïve Untreated 24 h
2: PC-3 naïve 20 nM BTZ 24 h
3: PC-3 naïve 40 nM BTZ 24 h
4: X
5: PC-3 RB40 Untreated 24 h
6: PC-3 RB40 20 nM BTZ 24 h
7: PC-3 RB40 40 nM BTZ 24 h
8: PC-3 RB40 80 nM BTZ 24 h
9: X
1 2 3 4 5 6 7 8 9
N-cadherin
(127 kDa)
MEK4
(44 kDa)
Final cropped image
Unidentified cross-reaction at about >150 kDa
E-cadherin
(135 kDa)
Final cropped image
$\alpha_v\beta_3$-integrin
(135, 100 kDa)
Unidentified cross-reaction at about 45 kDa
Final cropped image
β-catenin
(92 kDa)
Final cropped image
β-actin
(42 kDa)
Final cropped image

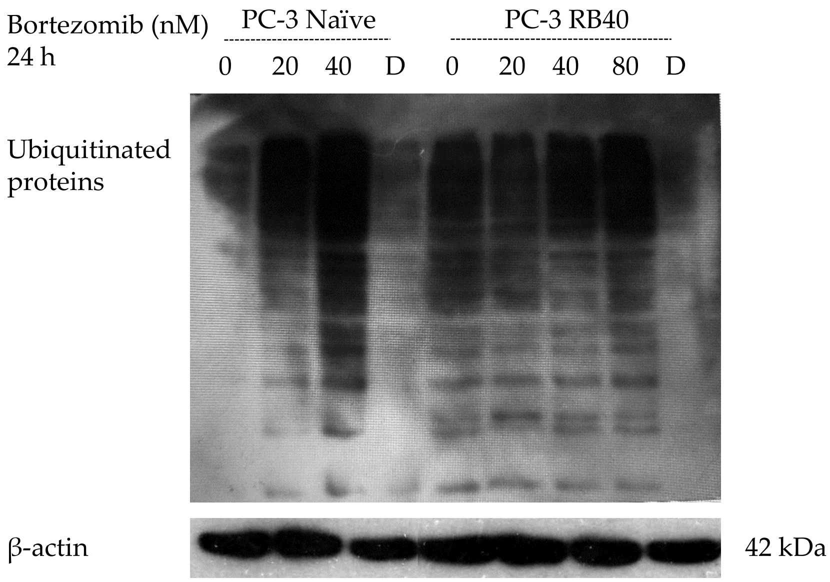

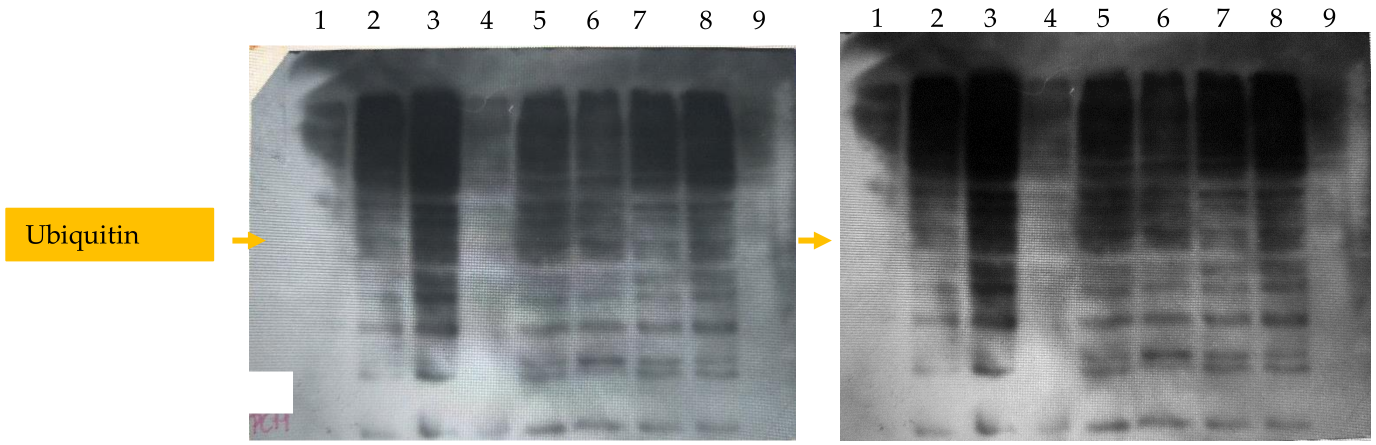

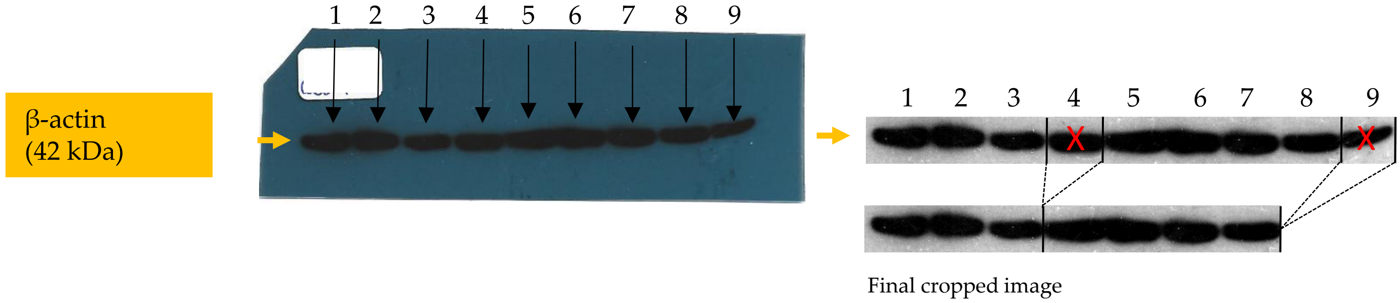

1: PC-3 naïve Untreated 24 h
2: PC-3 naïve 20 nM BTZ 24 h
3: PC-3 naïve 40 nM BTZ 24 h

4: PC-3 naïve Doxorubicin treated
5: PC-3 RB40 Untreated 24 h
6: PC-3 RB40 20 nM BTZ 24 h

7: PC-3 RB40 40 nM BTZ 24 h
8: PC-3 RB40 80 nM BTZ 24 h
9: PC-3 RB40 Doxorubicin treated

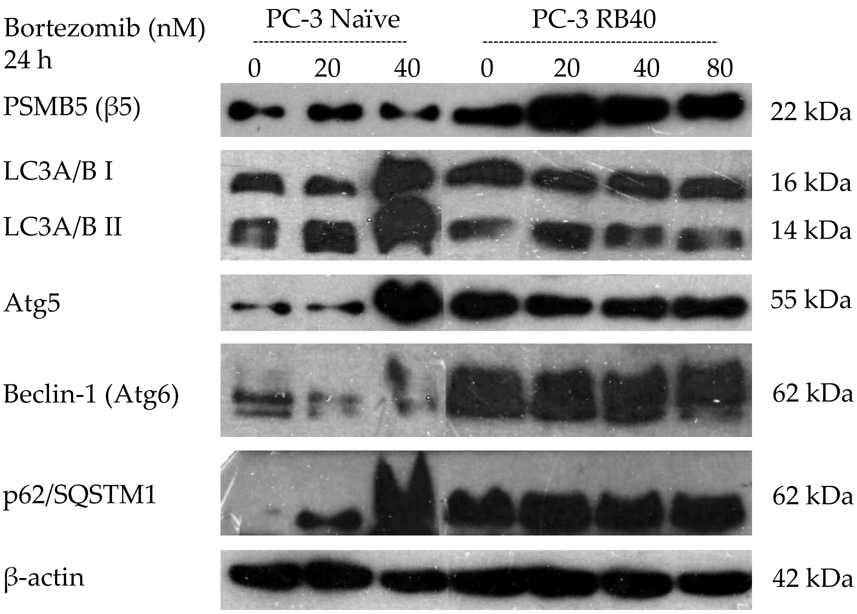

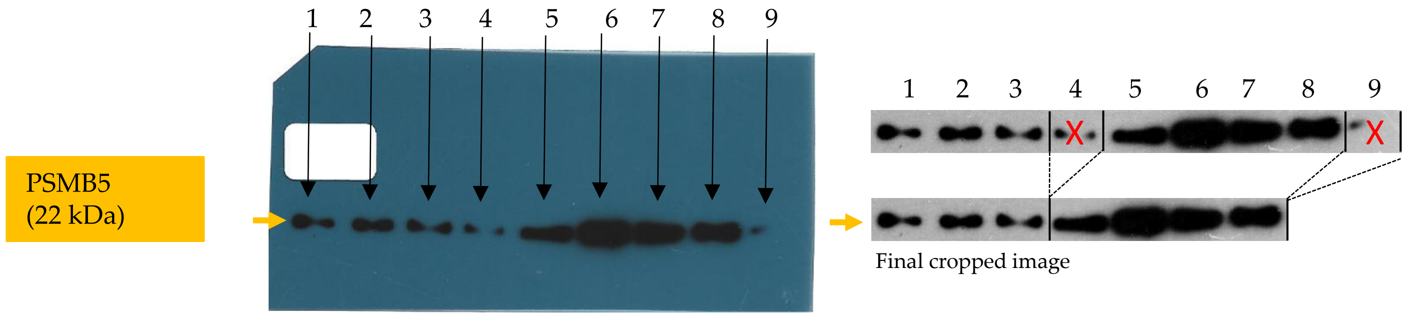

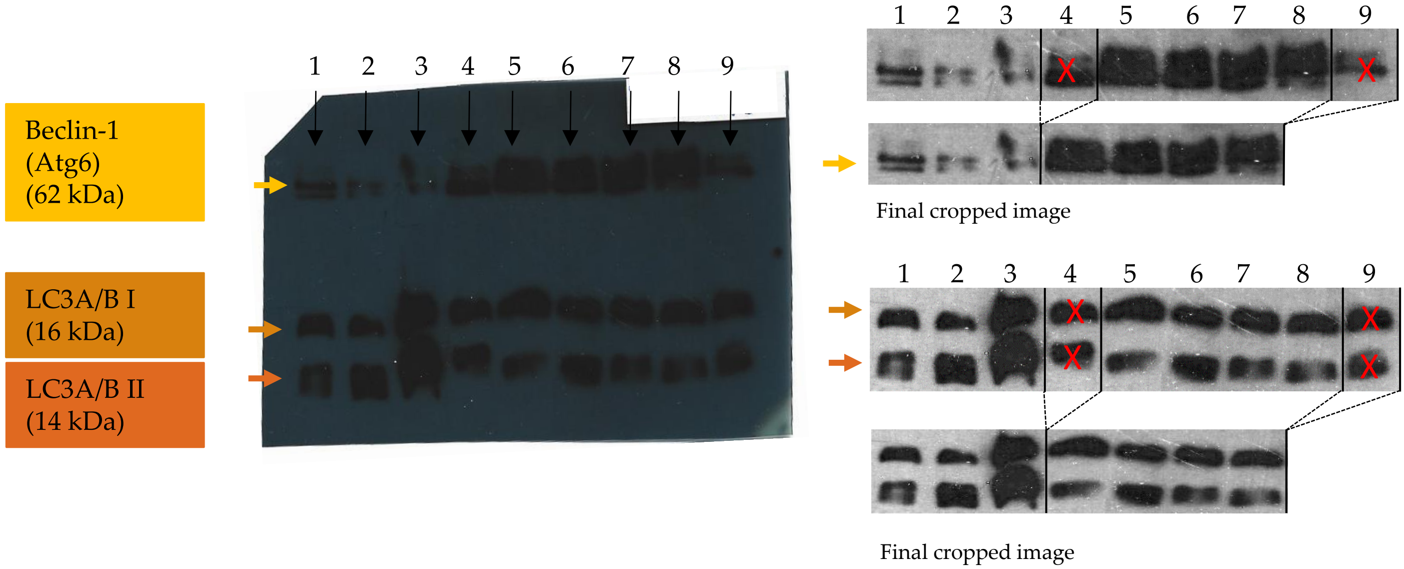

1: PC-3 naïve Untreated 24 h
2: PC-3 naïve 20 nM BTZ 24 h
3: PC-3 naïve 40 nM BTZ 24 h

4: X
5: PC-3 RB40 Untreated 24 h
6: PC-3 RB40 20 nM BTZ 24 h

7: PC-3 RB40 40 nM BTZ 24 h
8: PC-3 RB40 80 nM BTZ 24 h
9: X

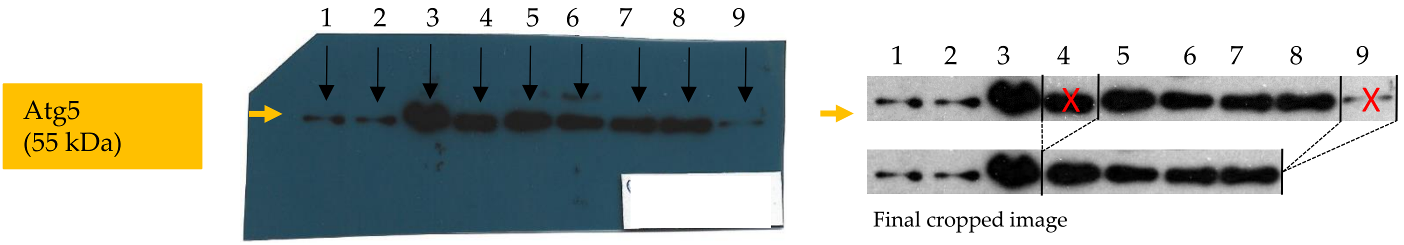

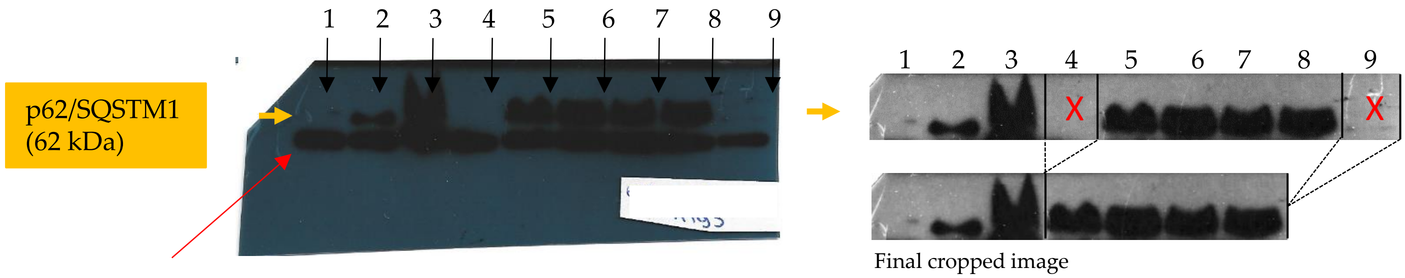

This is Atg5. Since no cross-reactions were detected with p62, the membranes were probed together. p62 needed more time to acquire adequate exposure film, there, Atg5 produces signal of high saturation

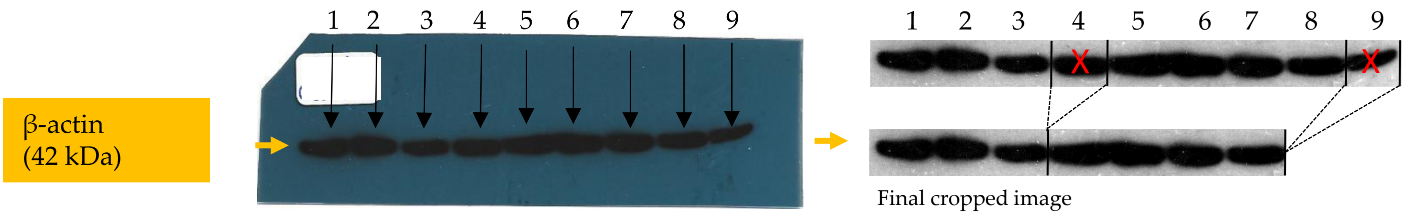

1: PC-3 naïve Untreated 24 h
2: PC-3 naïve 20 nM BTZ 24 h
3: PC-3 naïve 40 nM BTZ 24 h

4: X
5: PC-3 RB40 Untreated 24 h
6: PC-3 RB40 20 nM BTZ 24 h

7: PC-3 RB40 40 nM BTZ 24 h
8: PC-3 RB40 80 nM BTZ 24 h
9: X

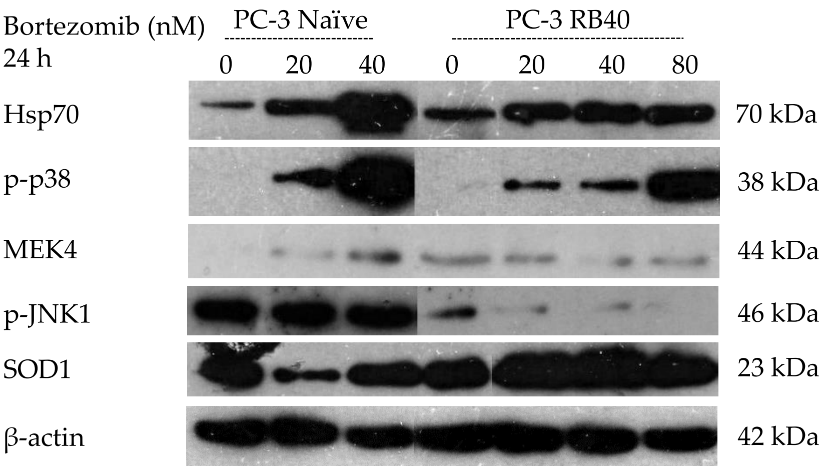

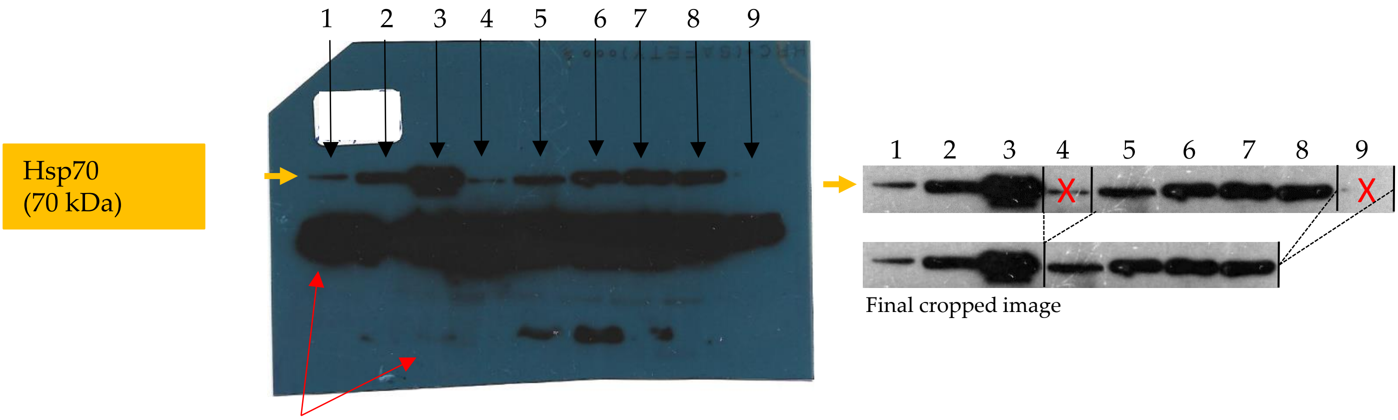

This is residual signal from ERK1/2 and PSMB5. The antibodies had been used alone in previous membranes and since no cross-reaction was detected, membranes were probed with them without striping; Therefore, due to long exposure time to get Hsp70 images, ERK1/2 produced signal of high saturation.

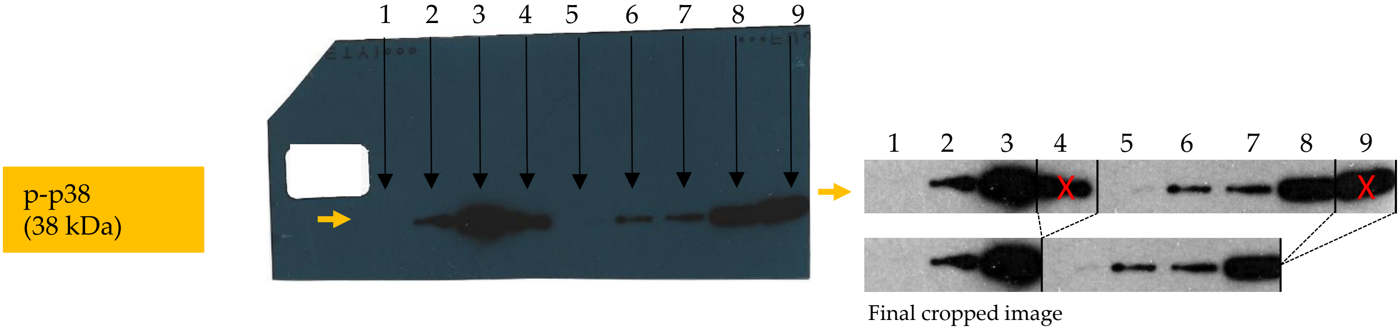

1: PC-3 naïve Untreated 24 h
2: PC-3 naïve 20 nM BTZ 24 h
3: PC-3 naïve 40 nM BTZ 24 h
4: X
5: PC-3 RB40 Untreated 24 h
6: PC-3 RB40 20 nM BTZ 24 h
7: PC-3 RB40 40 nM BTZ 24 h
8: PC-3 RB40 80 nM BTZ 24 h
9: X

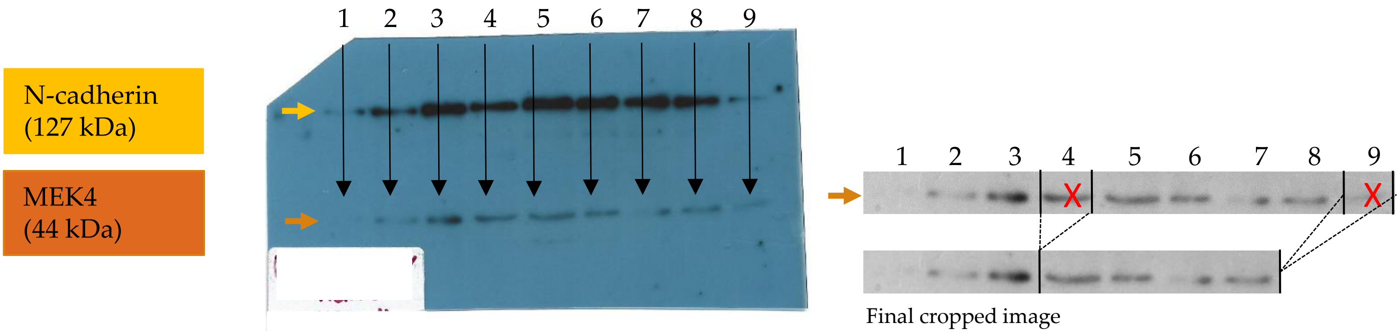

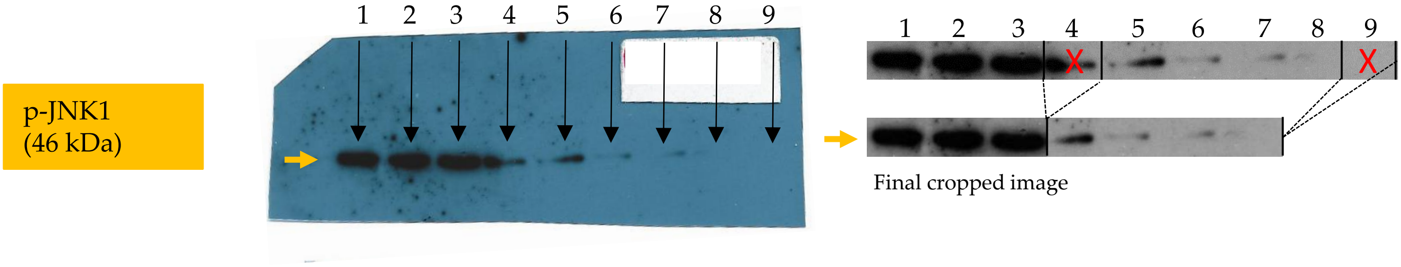

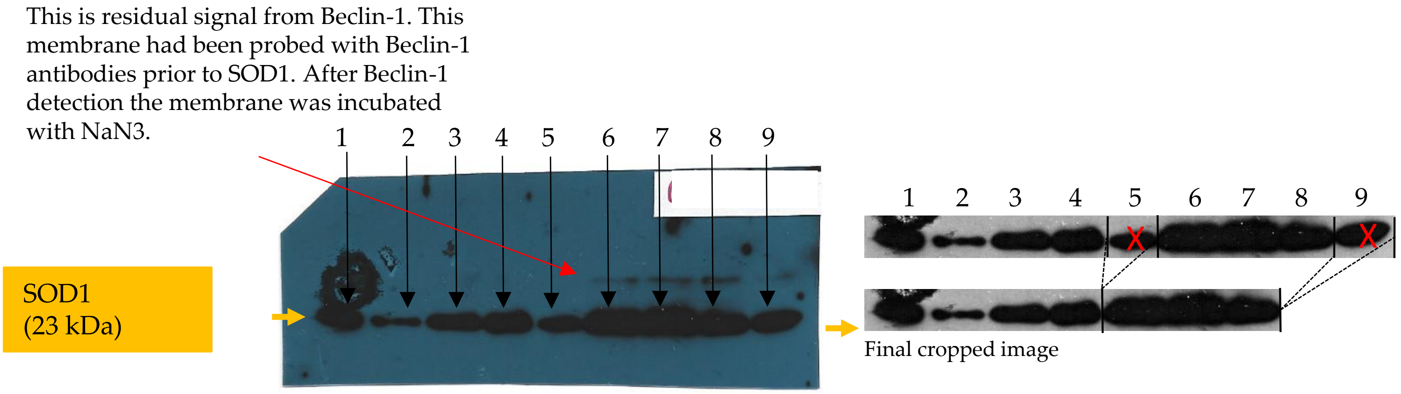

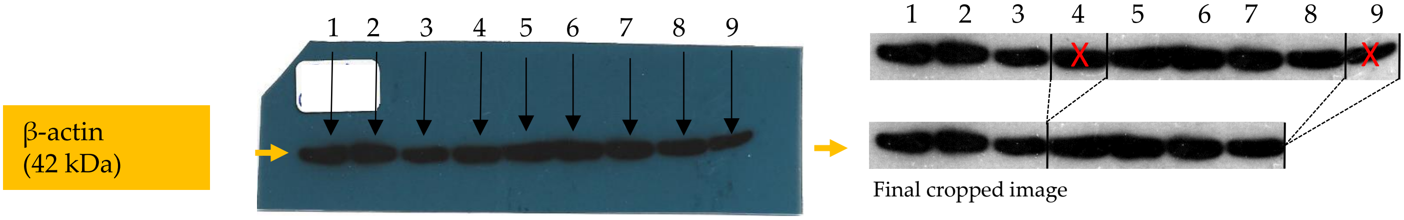

1: PC-3 naïve Untreated 24 h
2: PC-3 naïve 20 nM BTZ 24 h
3: PC-3 naïve 40 nM BTZ 24 h

4: PC-3 RB40 Untreated 24 h
5: X
6: PC-3 RB40 20 nM BTZ 24 h

7: PC-3 RB40 40 nM BTZ 24 h
8: PC-3 RB40 80 nM BTZ 24 h
9: X

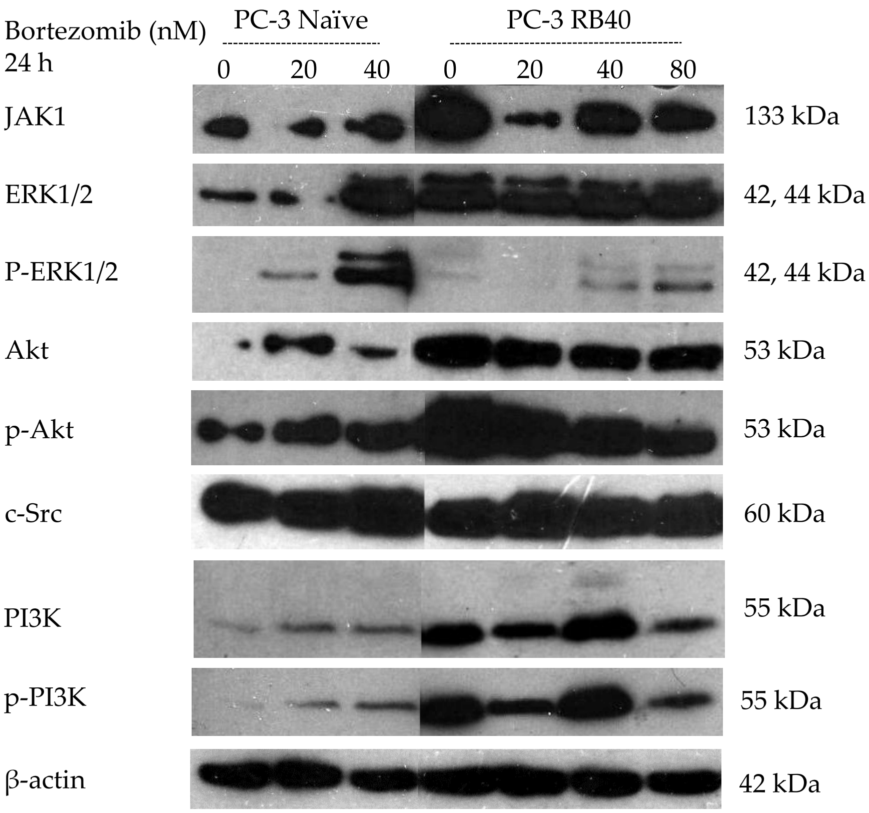

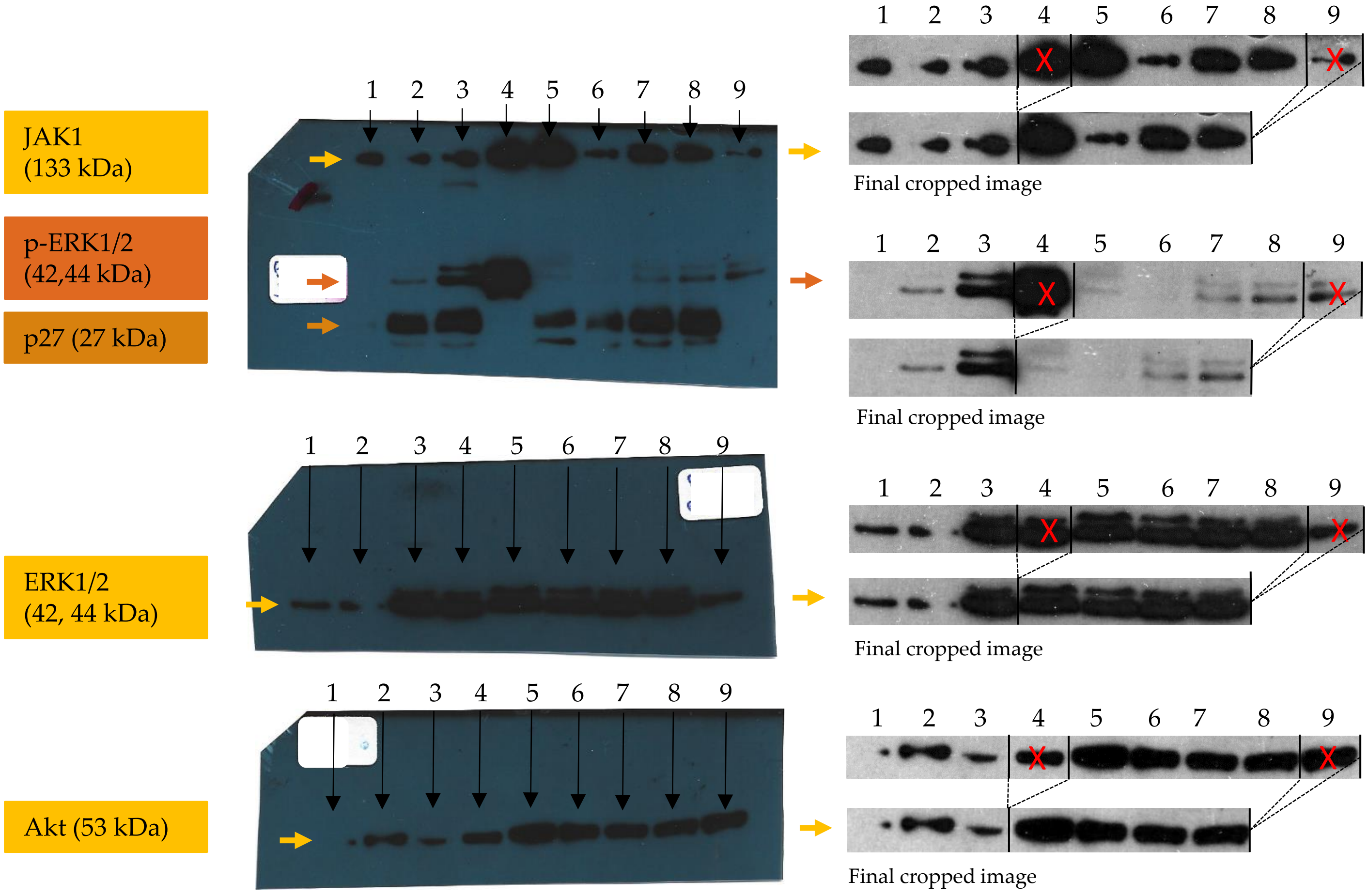

1: PC-3 naïve Untreated 24 h
2: PC-3 naïve 20 nM BTZ 24 h
3: PC-3 naïve 40 nM BTZ 24 h
4: X
5: PC-3 RB40 Untreated 24 h
6: PC-3 RB40 20 nM BTZ 24 h
7: PC-3 RB40 40 nM BTZ 24 h
8: PC-3 RB40 80 nM BTZ 24 h
9: X

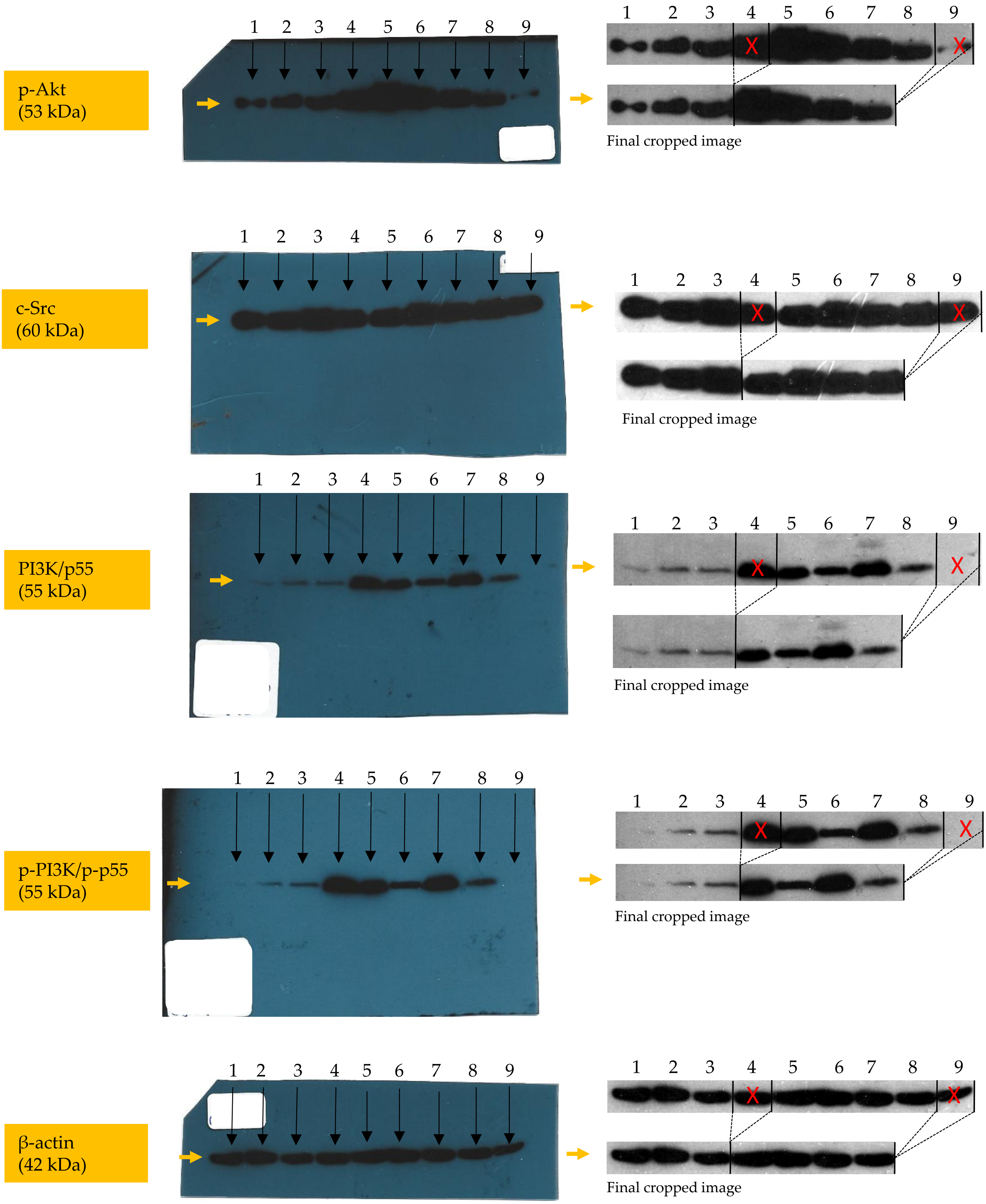

1: PC-3 naïve Untreated 24 h
2: PC-3 naïve 20 nM BTZ 24 h
3: PC-3 naïve 40 nM BTZ 24 h

4: X
5: PC-3 RB40 Untreated 24 h
6: PC-3 RB40 20 nM BTZ 24 h

7: PC-3 RB40 40 nM BTZ 24 h
8: PC-3 RB40 80 nM BTZ 24 h
9: X

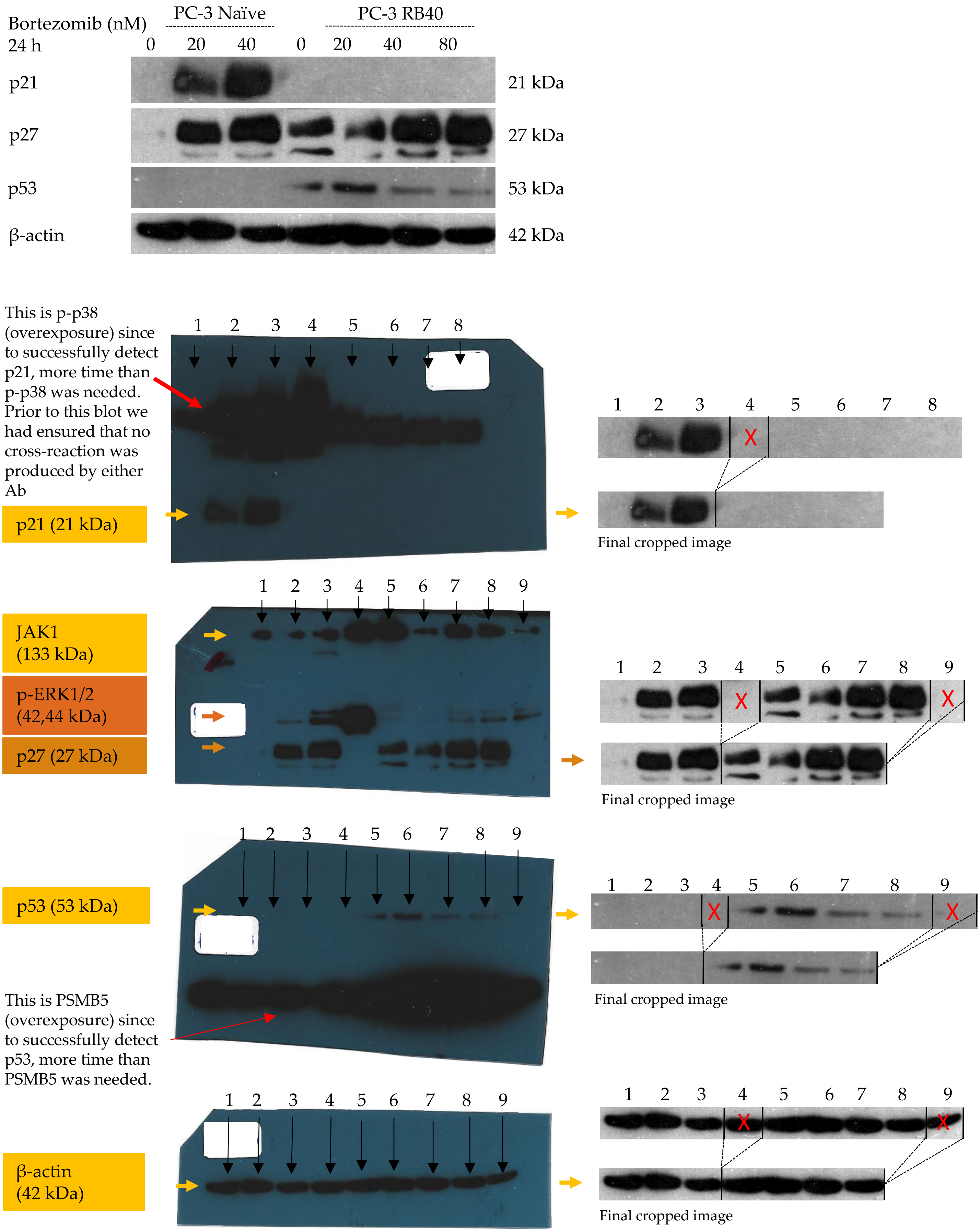

1: PC-3 naïve Untreated 24 h
2: PC-3 naïve 20 nM BTZ 24 h
3: PC-3 naïve 40 nM BTZ 24 h
4: X
5: PC-3 RB40 Untreated 24 h
6: PC-3 RB40 20 nM BTZ 24 h
7: PC-3 RB40 40 nM BTZ 24 h
8: PC-3 RB40 80 nM BTZ 24 h
9: X

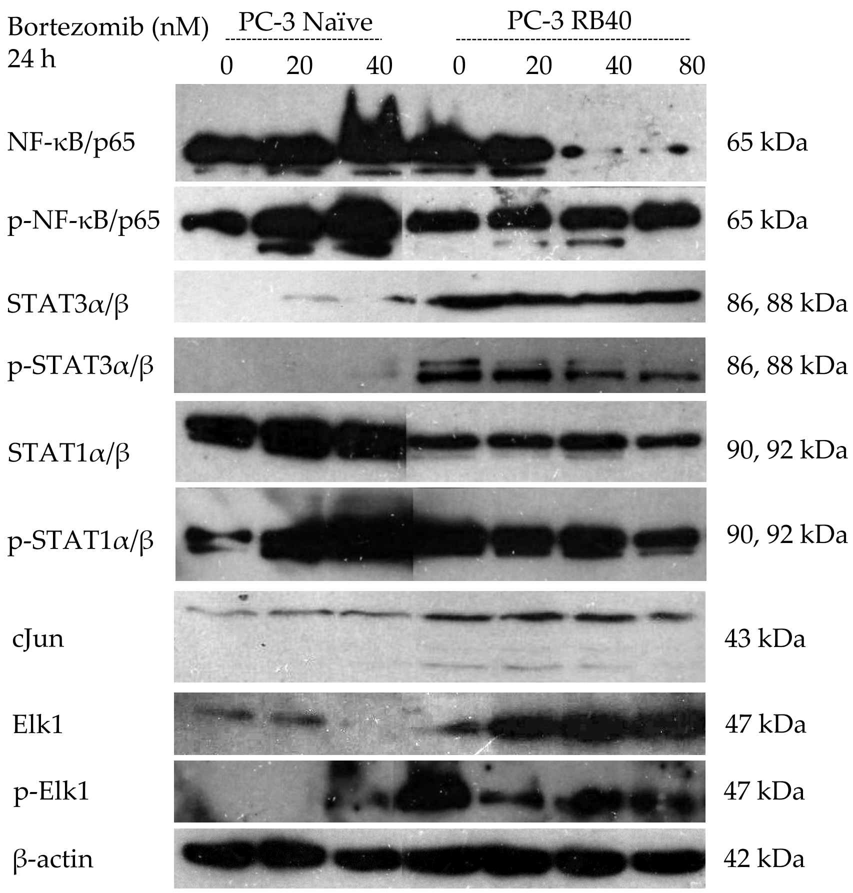

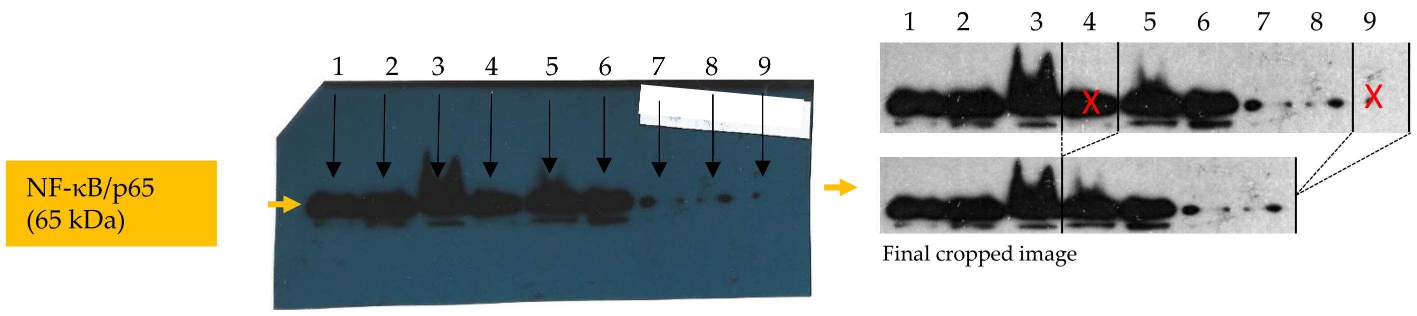

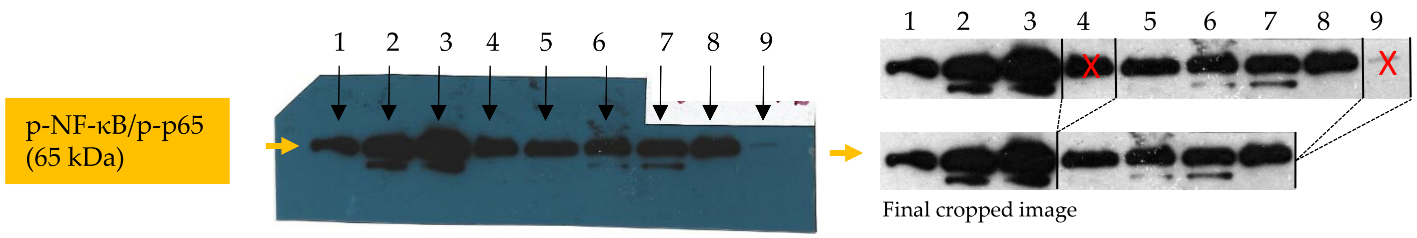

1: PC-3 naïve Untreated 24 h
2: PC-3 naïve 20 nM BTZ 24 h
3: PC-3 naïve 40 nM BTZ 24 h

4: X
5: PC-3 RB40 Untreated 24 h
6: PC-3 RB40 20 nM BTZ 24 h

7: PC-3 RB40 40 nM BTZ 24 h
8: PC-3 RB40 80 nM BTZ 24 h
9: X

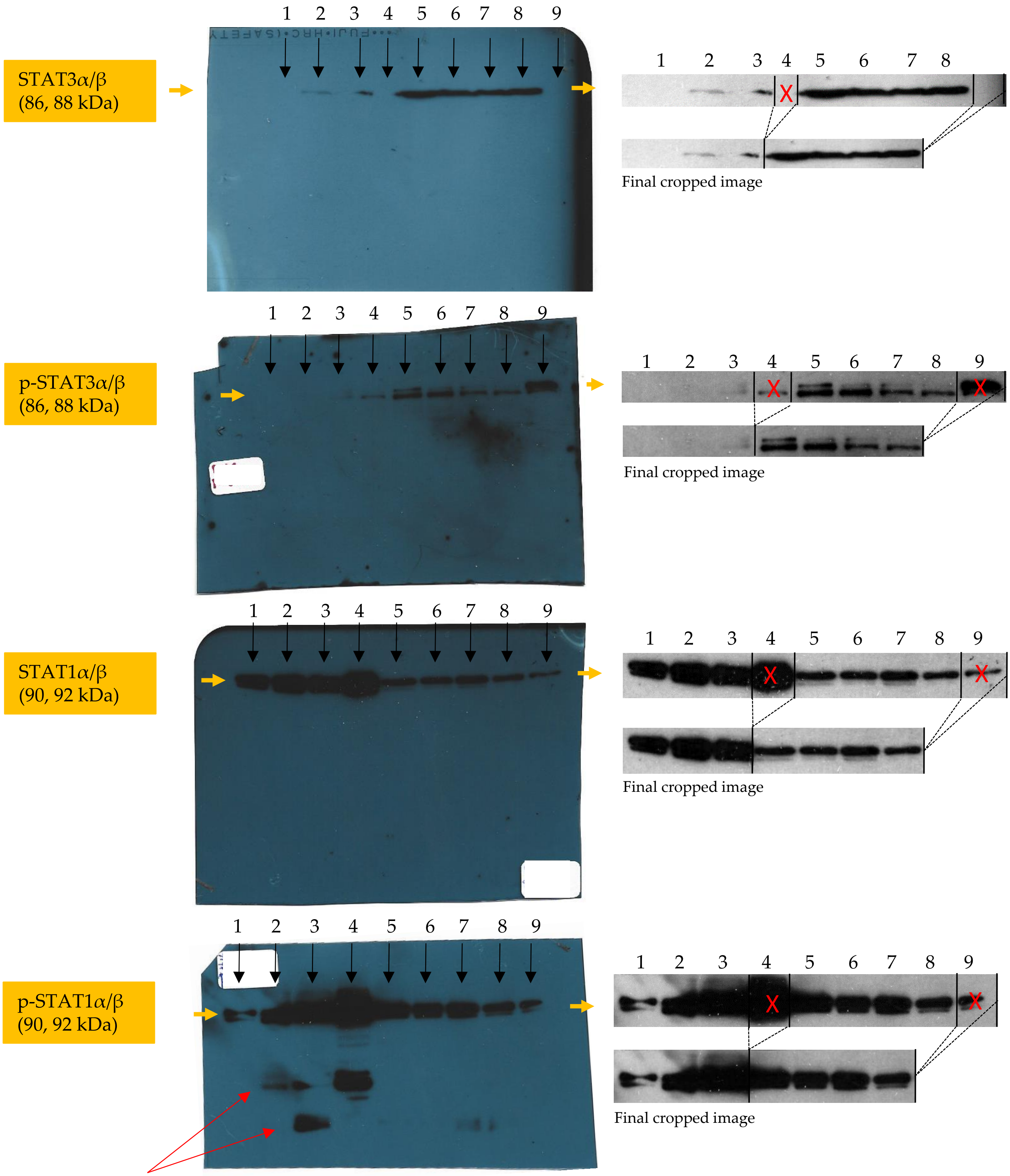

Previous to p-STAT1 incubation the membrane had been probed with Abs against P-ERK1/2 and p27. The membrane was incubated with NaN3 after p-ERK1/2 and p27 detection, and this is some residual signal escaping the NaN3 inactivation

1: PC-3 naïve Untreated 24 h
2: PC-3 naïve 20 nM BTZ 24 h
3: PC-3 naïve 40 nM BTZ 24 h
4: X
5: PC-3 RB40 Untreated 24 h
6: PC-3 RB40 20 nM BTZ 24 h
7: PC-3 RB40 40 nM BTZ 24 h
8: PC-3 RB40 80 nM BTZ 24 h
9: X

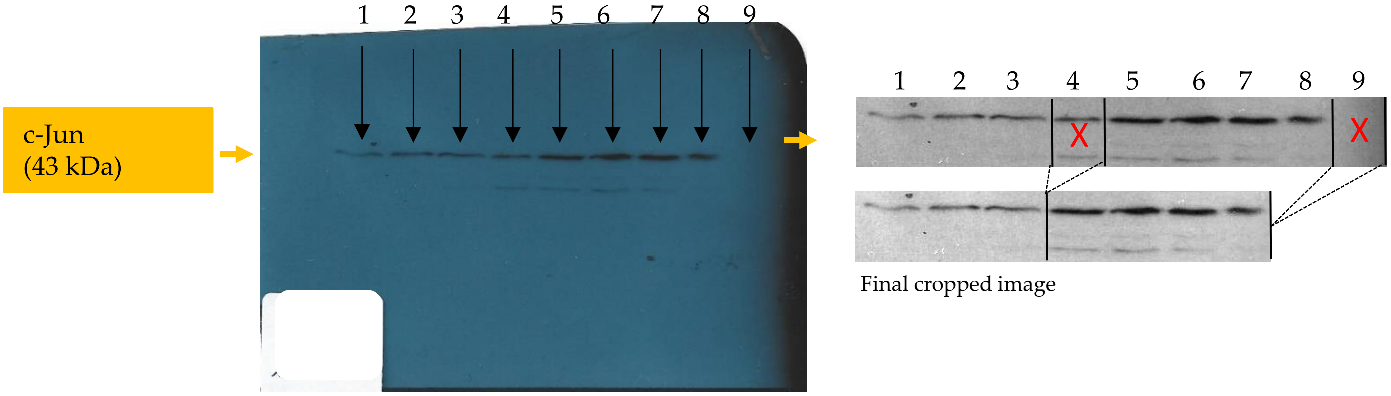

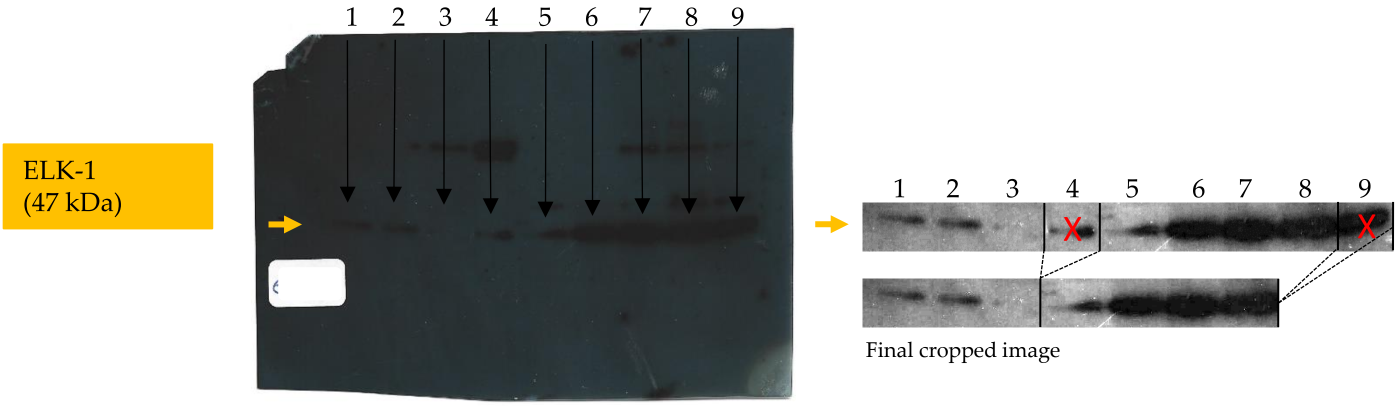

* This membrane had also been incubated with a Phospho- Focal Adhesion Kinase (MW = 125 kDa) antibody. We did not manage to obtain reliable images for our analysis; therefore, we did not include it to our manuscript nor we further continued assaying it.

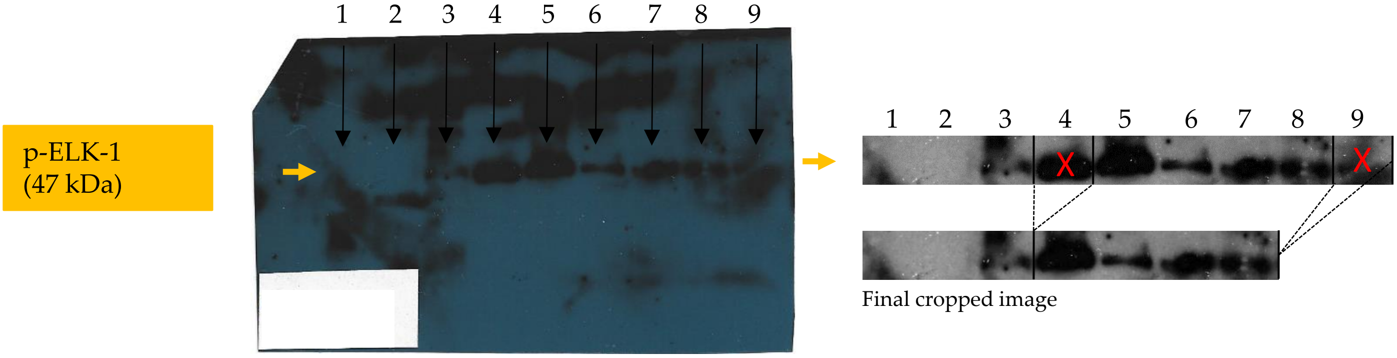

**This membrane had also been incubated with a Focal Adhesion Kinase (MW = 125 kDa) antibody. We did not manage to obtain reliable images for our analysis; therefore, we did not include it to our manuscipt nor we further continued assaying it.

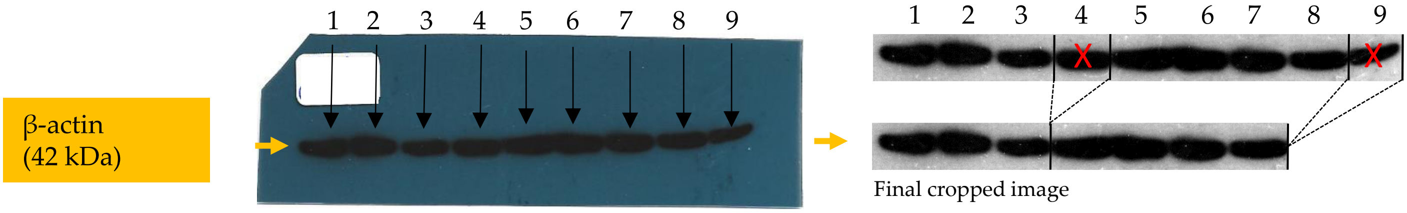

1: PC-3 naïve Untreated 24 h
2: PC-3 naïve 20 nM BTZ 24 h
3: PC-3 naïve 40 nM BTZ 24 h

4: X
5: PC-3 RB40 Untreated 24 h
6: PC-3 RB40 20 nM BTZ 24 h

7: PC-3 RB40 40 nM BTZ 24 h
8: PC-3 RB40 80 nM BTZ 24 h
9: X

Notes

- The films were scanned, and the images were transformed int grayscale pictures using ImageJ.
- The only adjustments made were Brightness and Contrast alterations, which were used to create the images appearing in the figures. For quantification of the signal, no adjustment had been made, as not to change the bit depth and intensity.
- The quantification was performed using the 'gels' tool by ImageJ.
- Some images are cropped, and every time cropping was performed, we present it here using arrows and lines.
- Some images have white tags on them, due to some paper tags attached on the films for identification. Due to privacy issues, we covered them with a white box when present. All tags were away from protein bands.
- During SDS-PAGE molecular weight markers were used; based on their position on the membrane we annotate here the observed molecular weight of each polypeptide detected
- Every cropping, alteration or adjustment performed, did not affect the image's content or meaning.
